# A broad-spectrum anti-fungal effector dictates bacterial-fungal interkingdom interactions

**DOI:** 10.1371/journal.ppat.1013598

**Published:** 2025-10-27

**Authors:** Shuangquan Yan, Yun Zou, Tingting Wu, Yumin Kan, Han Luo, Tong-Tong Pei, Xiaoye Liang, Ying An, Pengfei Meng, Yi Song, Wen-Ming Qin, Changbin Chen, Tao Dong

**Affiliations:** 1 State Key Laboratory of Microbial Metabolism, Joint International Research Laboratory of Metabolic and Developmental Sciences, School of Life Sciences and Biotechnology, Shanghai Jiao Tong University, Shanghai, China; 2 The Center for Microbes, Development, and Health, Key Laboratory of Molecular Virology and Immunology, Unit of Pathogenic Fungal Infection & Host Immunity, Shanghai Institute of Immunity and Infection, Chinese Academy of Sciences, Shanghai, China; 3 National Facility for Protein Science in Shanghai, Shanghai Advanced Research Institute, Chinese Academy of Sciences, Shanghai, China; 4 Department of Immunology and Microbiology, School of Life Sciences, Guangming Advanced Research Institute, Southern University of Science and Technology, Shenzhen, Guangdong, China; 5 Nanjing Advanced Academy of Life and Health, Nanjing, China; Centre National de la Recherche Scientifique, Aix-Marseille Université, FRANCE

## Abstract

Bacteria–fungi interactions play crucial roles in shaping microbial communities across diverse environmental and host-associated niches. While their antagonism through diffusible metabolites is a well-known ecological phenomenon, delivery of bacterial effectors into the nucleus of fungal cells remains rare, and the mechanisms are poorly understood. Here, we identify and characterize a potent anti-fungal nuclease effector, TseN, delivered by the type VI secretion system (T6SS) of *Acidovorax citrulli*. TseN possesses a nuclear localization signal and inhibits multiple fungal species, including emerging human pathogens *Candida auris* and *Cryptococcus neoformans*. Structural and biochemical analyses show that TseN possesses a unique C-terminal DNase domain that defines a new effector class, with its activity neutralized by a cognate immunity protein TsiN. The delivery of TseN requires the upstream-encoded VgrG5, a chaperone Aave_2128, and PAAR5. Transcriptome profiling of co-cultured bacterial-fungal cells demonstrates that the T6SS attack triggers extensive reprogramming in fungal cells, affecting DNA repair, stress response, and filamentation pathways. These responses not only compromise fungal survival but also modulate fungal drug resistance, as evidenced by the synergistic enhancement of azole efficacy against drug-resistant *Candida albicans*. Importantly, *in vivo* experiments confirm that the T6SS, via TseN, can significantly reduce fungal burden on murine skin. Phylogenetic analysis shows TseN homologs are present in a large number of bacterial species. Collectively, our findings highlight a previously underappreciated interkingdom antagonism modulated by a novel effector. The broad-spectrum anti-fungal activities of TseN and its homologs may be explored for therapeutic strategies targeting fungal pathogens in both clinical and environmental settings.

## Author summary

Multidrug-resistant fungal pathogens are an escalating global health concern, yet current options for treatment and for reducing environmental transmission remain limited. In nature, bacteria and fungi frequently compete for resources, providing an important source of new antifungal strategies. Here, we show that the plant-associated bacterium *Acidovorax citrulli* uses a needle-like type VI secretion system (T6SS) to inject a DNase effector, TseN, directly into fungal cells. Once delivered, TseN employs its nucleolar localization sequence to enter the fungal nucleus and damage DNA, thereby suppressing growth across diverse fungal species, including drug-resistant human pathogens. In mouse infection models, TseN-mediated activity reduced fungal burden in skin infections. The widespread distribution of TseN homologs suggests this represents a conserved mechanism of bacterial antagonism against fungi. These findings highlight new opportunities to develop innovative strategies to combat fungal infections in humans, animals, and plants.

## Introduction

Bacteria and fungi frequently co-inhabit complex environments where competition for space and nutrients shapes community composition. Antagonistic interactions between these kingdoms play crucial roles in soil, plant-associated microbiomes, and host environments [1–3]. While bacterial inhibition of fungi has long been documented, such interactions are typically attributed to the secretion of diffusible secondary metabolites, such as antibiotics, siderophores, or volatile compounds [1]. In contrast, the direct delivery of protein effectors into fungal cells remains a rare and poorly understood phenomenon.

Of the known complex interactions between bacteria and fungi [1,4], the mechanisms underlying the type VI secretion system (T6SS)-mediated interactions remain poorly understood. The T6SS is an effective molecular spear-like weapon that Gram-negative bacteria use to deliver effector proteins into neighboring cells, including bacteria and eukaryotes [5,6]. Featuring a long contractile double tubular structure anchored to a transmembrane complex, the T6SS has multiple routes to load toxic effectors to the spear that is subsequently ejected directly into neighboring recipient cells. These effectors exhibit diverse functions, including amidase [7], peptidoglycan hydrolase [8], phospholipase [9,10], nuclease [11–13], and NAD(P)(+) glycohydrolase [13,14]. T6SS has been extensively studied in the context of interbacterial competition and, more recently, as a tool for interactions with fungal competitors. Bacteria with known antifungal T6SSs include *Serratia marcescens* [5], *Klebsiella pneumoniae* [15], *Chryseobacterium gleum* [16], and *Acinetobacter baumannii* [17]. Although the T6SS in *Vibrio cholerae* exhibits strong anti-eukaryotic functions [18–20], it has little effect on yeast survival, highlighting that antifungal functions are not ubiquitous in T6SSs [21]. Additionally, while some T6SS effectors with antifungal activity have been described, none have been shown to enter fungal nuclei directly. The potential of the T6SS as a cross-kingdom weapon in microbial ecology is just beginning to emerge.

Fungal pathogens are increasingly recognized as widespread members of environmental and host microbiomes [3]. Several species, including *Candida auris* and *Cryptococcus neoformans*, have been isolated from plant surfaces, water sources, and soil, where they coexist with bacteria [22,23]. The rise of antifungal resistance and the increased detection of these pathogens in nonclinical reservoirs underscore the importance of understanding how bacterial competitors might naturally suppress their growth and transmission across ecosystems. In 2023, a severe outbreak of multidrug-resistant *C. auris* in the United States, with a high mortality of up to 60%, underscores the urgent need for a concerted effort to reduce environmental transmission and human infection of fungal pathogens [24,25]. Given the ubiquitous presence of bacteria and fungi, exploring their natural interactions may offer novel approaches to control fungal pathogens.

In this study, we identify and characterize TseN, a previously unrecognized T6SS effector from *Acidovorax citrulli*, which exhibits one of the most potent and broad-spectrum T6SS functions against Gram-negative and Gram-positive bacteria as well as fungi [26]. TseN possesses a unique nuclease-like domain and a nuclear localization signal. The structural fold of TseN is distinct from known T6SS effectors or antifungal proteins, and its activity is effective against multiple WHO-priority pathogens including *C. albicans*, *C. auris*, and *C. neoformans*. In mouse infection models, the *A. citrulli* T6SS reduced *C. auris* infection, with TseN playing a key role. Our findings not only uncover a molecular mechanism by which bacteria can target fungal competitors via direct effector delivery, but also suggest a broader ecological and functional role for T6SS in limiting the persistence of fungal pathogens in the environment.

## Results

### TseN is the primary antifungal T6SS effector in *A. citrulli*

**Fig 1:**
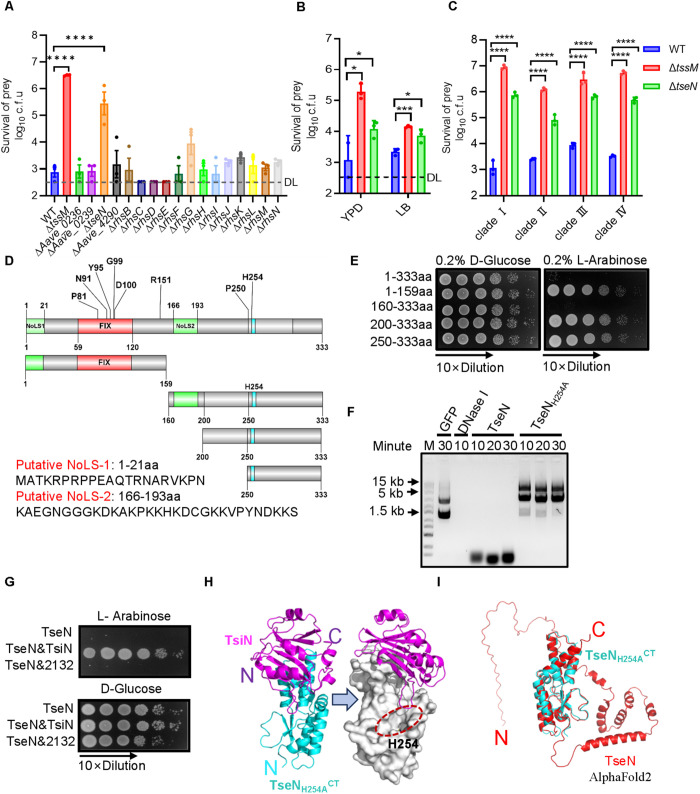
Identification of TseN as the most critical T6SS effector for antifungal activities in *Acidovorax citrulli.* **(A)** Competition assay of wild type (WT), the T6SS-null Δ*tssM* mutant, and effectors mutant against *P. pastoris* on an LB agar plate at 28°C for 3 hours. Survival of prey cells was determined by serial dilutions on selective media. **(B)** Competition assay of wild type (WT), the T6SS-null Δ*tssM* mutant, and Δ*tseN* against *S. cerevisiae* on YPD or LB agar plate at 30°C for 16 hours. Survival of prey cells was determined by serial dilutions on selective media. **(C)** Competition assay of wild type (WT), the T6SS-null Δ*tssM* mutant, and Δ*tseN* against the multiple clades of *C. auris* on a YPD agar plate at 30°C for 8 hours. **(D)** Schematic depicting putative DNase structural domains, NoLS sequences, conserved residues, and truncation mutants in TseN. **(E)** Toxicity of expressing full-length and truncated regions of TseN on pBAD vectors in *E. coli.* The survival of *E. coli* was tested by serial plating on 0.2% arabinose (induction) and 0.2% glucose (repression) plates with 10-fold dilutions. **(F)** DNA degradation by TseN and its mutant TseN_H254A_. Purified 600 ng pUC19 plasmids were treated with GFP, DNase I, TseN, and TseN_H254A_ proteins. DNA was sampled at the indicated time points and examined by electrophoresis on an agarose gel. For each 5 μl reaction, 0.5 μl of 10 × CutSmart buffer and either 100 ng of TseN or TseN_H254A_ were used. Commercial DNase I (1 unit) was used as a positive control, and GFP protein (100 ng) was used as a negative control. **(G)** Toxicity of co-expressing *tseN* with the immunity gene *tsiN* or *Aave_2132* together as indicated in *E. coli*. All constructs were cloned on pBAD vectors, and the survival of *E. coli* was enumerated by serial plating on 0.2% arabinose (induction) and 0.2% glucose (repression) plates. **(H)** The overall structure of TseN_H254A_^CT^ and its immunity TsiN complex at 2.6 Å resolution (R_free_ = 0.2846, R_work_ = 0.2309) and the α2-β1 loop of TsiN is juxtaposed to the catalytic domain of TseN (H254). **(I)** Superposed crystal structure of TseN_H254A_^CT^ (cyan) with TseN (red) predicted by AlphaFold2, the RMSD = 0.922. Error bars indicate the standard deviation of three biological replicates and statistical significance was calculated using a One–way ANOVA for panels A, and others using a two-tailed Student’s *t*-test. *p < 0.05, ***p < 0.001, ****p < 0.0001. DL, detection limit.

To investigate the antifungal mechanism of the T6SS in *A. citrulli*, we performed competition assays using an effector-gene mutant library generated and described in our previous study [26] against *Pichia pastoris* on LB agar plates. By comparing the survival of *P. pastoris* after co-incubation, we found that the T6SS-mediated killing was significantly impaired in the *tseN* mutant (*Aave_2130*) (Figs 1A and S1A). Similarly, competition analysis with several other fungal species, *Saccharomyces cerevisiae* (*S. cerevisiae*) (Figs 1B and S1B), *C. auris* (Figs 1C and S1C), *C. tropicalis* (S1D–S1E Fig)*, C. glabrata* (S1F–S1G Fig), *C. neoformans* (S1H–S1I Fig), and *A. fumigatus* (S1J–S1L Fig) showed significant killing by T6SS, whereas deletion of *tseN* significantly impaired the killing against these fungal species except *A. fumigatus*. Notably, all four major geographical clades of *C. auris*, including the multi-drug-resistant clade Ⅰ, exhibited higher sensitivity to the T6SS killing than *S. cerevisiae*. These results indicate that TseN is the primary antifungal effector of the *A. citrulli* T6SS.

### TseN displays DNase activity

To examine the function of TseN, we performed sequence analysis using Blastp and CD-Search and found no conserved domains with known functions, except for an N-terminal FIX motif [27] that has been found in some T6SS effectors and two putative nucleolar localization sequences (NoLSs) (Fig 1D). Sequence alignment of TseN with its homologs revealed several highly conserved residues (S2 Fig), which were subsequently mutated to alanine to assess their effects (Fig 1D). The toxicity assay showed that only the H254A mutant lost its toxicity, indicating H254 as a crucial residue for toxicity (Figs 1D and S3A–S3B). Next, we constructed a series of truncation mutants and expressed them in *E. coli* T-Fast using an arabinose-inducible pBAD vector. The results show that both full-length TseN and its C-terminal domain (160–333aa) were toxic (Figs 1E and S3C).

The presence of putative NoLS in TseN suggests that it may target the nucleus. To test whether TseN functions as a DNase, we purified TseN and its H254A mutant (S3D Fig) and performed *in vitro* DNA degradation assays. Wild-type TseN but not the H254A mutant degraded plasmid and genomic DNA samples (Figs 1F and S3E). These findings demonstrate that TseN functions as a potent DNase.

### TseN and TsiN form an effector-immunity pair

Given that effectors often exist in pairs with their cognate immunity proteins, we next tested whether the downstream-encoded proteins protect against TseN toxicity. Using the arabinose-inducible pBAD vectors, we expressed *tseN* along with its downstream genes, *tsiN* (Aave_2131) or Aave_2132, in *E. coli*. The results showed that only TsiN effectively neutralized toxicity (Figs 1G and S3F–S3G). To examine the interaction between TseN and TsiN, we employed bacterial two-hybrid assays based on a split adenylate cyclase [28,29]. To mitigate toxicity, we used the nontoxic TseN_H254A_ variant. Results exhibited a clear interaction between TseN_H254A_ and TsiN (S3H Fig).

To gain structural insights into the molecular mechanism of TseN activity and TsiN inhibition, we purified the TseN_H254A_^CT^-TsiN complex (S3I Fig) and resolved the crystal structure at 2.6 Å resolution (PDB ID 9J9W; R_free_ = 0.2846, R_work_ = 0.2309; S1 Table) (Fig 1H). The C-terminal DNase of TseN is well folded, consisting of seven α-helices, one 3_10_-helix, and a three-stranded antiparallel β-sheet (S3J Fig). The putative catalytic pocket, where the H254 residue is located, is concave and centrally positioned within this domain (Fig 1H).

The immunity protein TsiN adopts an α/β fold with four α-helices, one 3_10_-helix, and a seven-stranded antiparallel β-sheet (S3K Fig). The interface between TseN-TsiN is stabilized by 5 salt bridges, 15 hydrogen bonds, and 118 non-covalent contacts (S3L Fig). This interface involves 24 amino acid residues from TsiN and 19 residues from four helical regions (α1, α2, α5, and 3_10_) in the DNase domain. The α2-β1 loop of TsiN is positioned above the DNase catalytic pocket (Fig 1H), likely obstructing TseN access to DNA substrates.

We predicted a full-length TseN structure by AlphaFold2. The N-terminal sequence of TseN is largely disordered, followed by several alpha helices connected by non-structural loops. The predicted structure closely matched the crystallized C-terminal DNase domain (RMSD = 0.922) (Fig 1I), suggesting that TsiN binding induces minimal conformational changes in TseN (Fig 1I). Notably, the absence of similar structures in the PDB database indicates that TseN may represent a novel class of DNase.

### VgrG5, PAAR5, and a chaperone are required for TseN-associated killing

**Fig 2:**
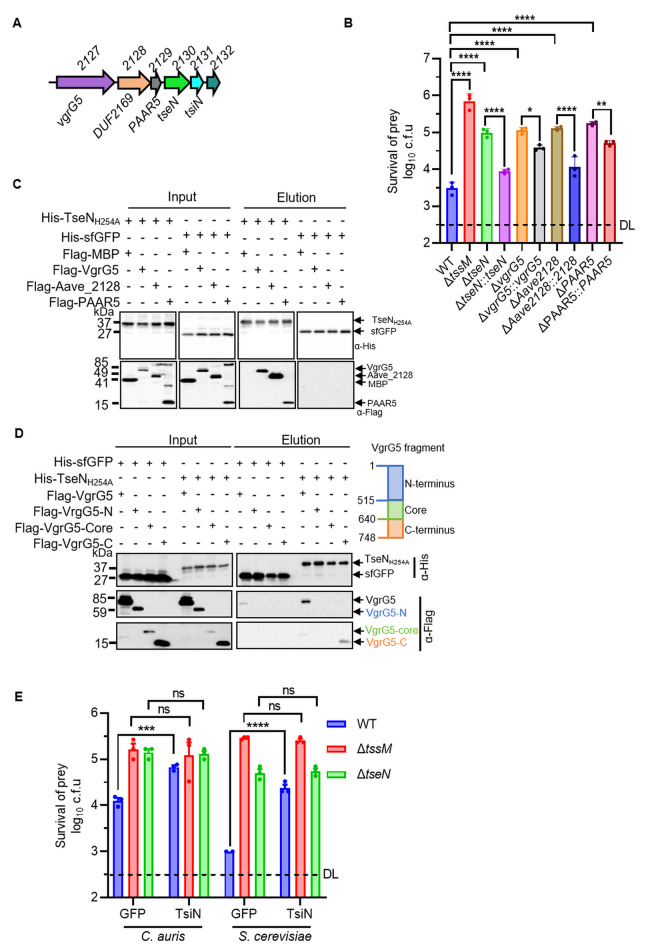
Killing of TseN requires VgrG5, PAAR5 and a chaperone. **(A)** Operon structure of *tseN*. The immunity gene *tsiN*, chaperone *Aave_2128*, and *PAAR5* (*Aave_2129*) are not annotated in the draft genome. (**B**) Competition assay of WT, *ΔtssM*, *ΔtseN*, *ΔvgrG5*, *ΔAave_2128*, *ΔPAAR5* mutants, and their complementary *A. citrulli* strains (*tseN*-, *vgrG5*-, *Aave_2128*-, *PAAR5*-complementary strains) against the prey cell *C. auris*. The prey cells and killer cells were mixed at a ratio of 10 (killer: prey) and spotted onto YPD agar plates and incubated for 3 hours at 30°C. Survival of prey cells was determined by serial dilutions on selective media. **(C)** Pull-down analysis of TseN_H254A_ with VgrG5, Aave_2128, and PAAR5. Pull-down analysis was performed using His-tagged sfGFP (control), TseN_H254A_, and FLAG-tagged MBP (control), VgrG5, Aave_2128, or PAAR5. The use of His-tagged sfGFP serves as a control to confirm that the FLAG-tagged proteins do not interact nonspecifically with the Ni-NTA beads or the His tag. The FLAG-tagged MBP control demonstrates that the FLAG tag alone does not interact nonspecifically with the Ni-NTA resin or His-tagged proteins. **(D)** Pull-down analysis of TseN_H254A_ mutant with full-length/ N-terminus/ Core/ C-terminus of VgrG5. The use of His-tagged sfGFP serves as a control to confirm that the FLAG-tagged proteins do not interact nonspecifically with the Ni-NTA beads or the His tag. **(E)** Competition assay of WT, Δ*tssM,* and Δ*tseN* mutants against *C. auris* YCB885 (or *S. cerevisiae* TN124) expressing the immunity protein TsiN or GFP (control), as indicated. Killer and prey cells were mixed at a ratio of 10 (killer: prey) and spotted onto YPD agar plates with 100 ng/ml aTC for 3 hours (or YP agar plates with 2% D-galactose for 16 hours) at 30°C. Survival of prey cells was determined by serial dilutions on selective media. Error bars indicate the standard deviation of three biological replicates and statistical significance was calculated using a One-way ANOVA analysis, *p < 0.05, **p < 0.01, ***p < 0.001, ****p < 0.0001, ns, not significant. DL, detection limit.

The *tseN* gene cluster also encodes VgrG5 (Aave_2127), a DUF2169-domain chaperone (Aave_2128), and a PAAR-like domain-containing protein (Aave_2129) (Fig 2A). To evaluate the role of these upstream-encoded proteins in TseN functions, single-deletion mutants of *vgrG5*, *Aave_2128*, and *PAAR5* were generated and tested for their ability to compete with *C. auris* cells. The defect of the mutants (Δ*tseN*, Δ*vgrG5*, Δ*Aave_2128*, and Δ*PAAR5*) could be partially complemented using plasmid-borne expression of corresponding genes (Figs 2B and S4A–S4E). Notably, the full-length VgrG5 appeared to be highly unstable when expressed in the *vgrG5*-complementation strain, despite its functional complementation (S4B Fig).

Next, we tested whether there is direct interaction with these proteins using the pull-down assays. The results showed that TseN individually binds to the chaperone Aave_2128, VgrG5, and PAAR5 (Figs 2C and S4F). Further pull-down assays with truncated VgrG5 variants (N-terminus, core, and C-terminus) revealed that TseN primarily interacts with the C-terminus of VgrG5 (Figs 2D and S4G), consistent with observations in other species that the C-terminal tail of VgrG is the effector loading site [30].

To confirm that TseN is translocated into fungal cells, we heterologously expressed the immunity protein TsiN in *C. auris* and *S. cerevisiae* (Figs 2E and S4H), which conferred resistance to *A. citrulli* T6SS-mediated killing. These results suggest that TseN is translocated directly into fungal cells and further support its role as a primary antifungal effector of the T6SS.

### TseN localizes to the nucleus and exhibits cytotoxicity in fungi

**Fig 3:**
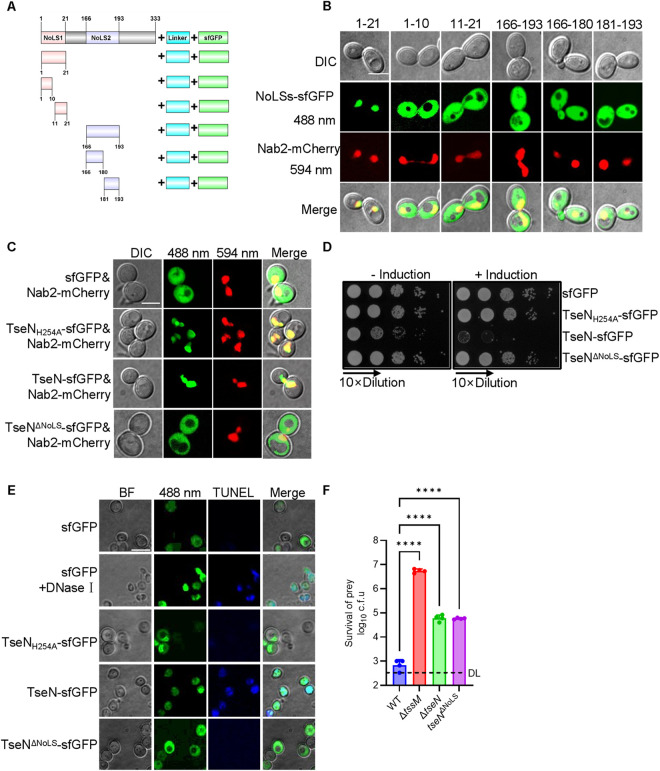
TseN localizes to the nucleus and exhibits cytotoxicity in fungi. **(A)** Construction of the fusion expression of NoLSs and sfGFP in *S. cerevisiae*. Depiction (not to scale) showing fusions of sfGFP tag (green) to putative NoLS1 and its truncated region (orange) or to putative NoLS2 and its truncated region (purple) with a flexible linker (aquamarine) inserted. **(B)** Fluorescence microscopy images show the localization of TseN-NoLSs-sfGFP to the nucleus in *S. cerevisiae* TN124 cells expressing Nab2-mCherry. NoLS stands for nucleolar localization sequences. DIC represents differential interference contrast images, 488nm represents TseN/TseN^ΔNoLS^/TseN_H254A_-sfGFP signals, 594nm represents Nab2-mCherry signals, and Merge represents the overlay of DIC, sfGFP and mCherry signals. The images were obtained at the same magnification with a 12 × 12-μm field. Scale bar, 5 μm. **(C)** Fluorescence microscopy images reveal the co-localization of TseN/TseN^ΔNoLS^/TseN_H254A_-sfGFP with the nuclear localization marker Nab2-mCherry in *S. cerevisiae* TN124. The images were obtained at the same magnification with a 12 × 12-μm field. Scale bar, 5 μm. **(D)** Toxicity of expressing TseN, TseN_H254A_, and TseN^ΔNoLS^ fused with sfGFP in Trp-deficient *S. cerevisiae* TN124 was enumerated with or without induction by D-galactose; the CFU was determined by serial plating on YNB plates. **(E)** Bright-field and confocal microscopy images demonstrating TUNEL-detected DNA breaks induced by the TseN’s DNase activity. Exponentially growing cells expressing sfGFP were fixed and subjected to TUNEL staining, with DNase I treatment serving as the positive control and untreated cells as the negative control. BF: Bright-field images, 488 nm: Combined signals from sfGFP and TseN/ TseN_H254A_/TseN^ΔNoLS^-sfGFP fusions, TUNEL: DNA break staining (640 nm) using the TUNEL assay kit, Merge: Overlay of BF, sfGFP and TUNEL signals. The images were obtained at the same magnification with a 5 × 5-μm field. Scale bar, 2 μm. **(F)** Competition assay of WT, Δ*tssM*, Δ*tseN*, and NoLS-deleted *tseN* mutant (*tseN*^ΔNoLS^) against the prey cell *C. auris* YCB885. Killer and prey cells were mixed at a ratio of 10 (killer: prey) and spotted on YPD agar plates for 3 hours at 30°C. Error bars indicate the standard deviation of four biological replicates and statistical significance was calculated using a One-way ANOVA analysis, ****p < 0.0001. DL, detection limit.

As shown in Fig 1D, two putative NoLS sequences were predicted in TseN. To investigate how TseN, a DNase-type effector, is translocated to the nucleus, we initially determined the target sequence that directs TseN to the nucleus. This was achieved by fusing a C-terminal superfolder GFP (sfGFP) tag to the predicted NoLSs (Fig 3A) and constructing a chromosomal Nab2-mCherry, a nuclear marker in *S. cerevisiae* TN124 [31]. Confocal microscopy results revealed that only the 1–21aa-sfGFP fusion exhibited nuclear localization, while the other constructs showed cytosolic distribution (Fig 3B).

To visualize TseN localization in fungal cells, sfGFP-tagged TseN, TseN_H254A_, and TseN^ΔNoLS (1-21aa)^ were employed. Confocal microscopy showed uniform cellular distribution of sfGFP. In contrast, sfGFP-tagged TseN and TseN_H254A_ fusions (>40 kDa) foci co-localized with Nab2-mCherry (Fig 3C). However, the deletion of NoLS^1-21aa^ (TseN^ΔNoLS^) abolished the co-localization with Nab2-mCherry, indicating that TseN localizes to the nucleus in *S. cerevisiae* cells and depends on NoLS^1-21aa^.

To determine the role of the NoLS in TseN-mediated fungal death, we induced the expression of plasmid-borne TseN-sfGFP, sfGFP only, the nontoxic TseN_H254A_-sfGFP, or TseN^ΔNoLS^-sfGFP in *S. cerevisiae* TN124 strain. Only the wild-type TseN-sfGFP was toxic, suggesting the deletion of NoLS abolished the toxicity of TseN in *S. cerevisiae* TN124. (Fig 3D).

To determine whether TseN causes DNA damage in fungal cells, TUNEL staining [32] was employed, combined with confocal microscopy, which reliably visualized DNA fragmentation. The results showed that endogenously expressed TseN caused DNA damage comparable to the DNase I positive control, whereas no signal was detected with sfGFP, catalytically inactive TseN mutants, or NoLS^1-21aa^ -deleted variants (Fig 3E).

Additionally, we constructed a chromosomal NoLS^1-21aa^-deletion (*tseN*^ΔNoLS^) in *A. citrulli*. Competition assays revealed significantly reduced killing efficiency against *C. auris*, comparable to that of the *tseN* deletion mutant (Figs 3F and S5). These results indicate that the nucleolar localization sequence (NoLS^1-21aa^) of TseN is crucial for its antifungal activity.

### T6SS reduces *C. auris* survival *in vivo*

**Fig 4:**
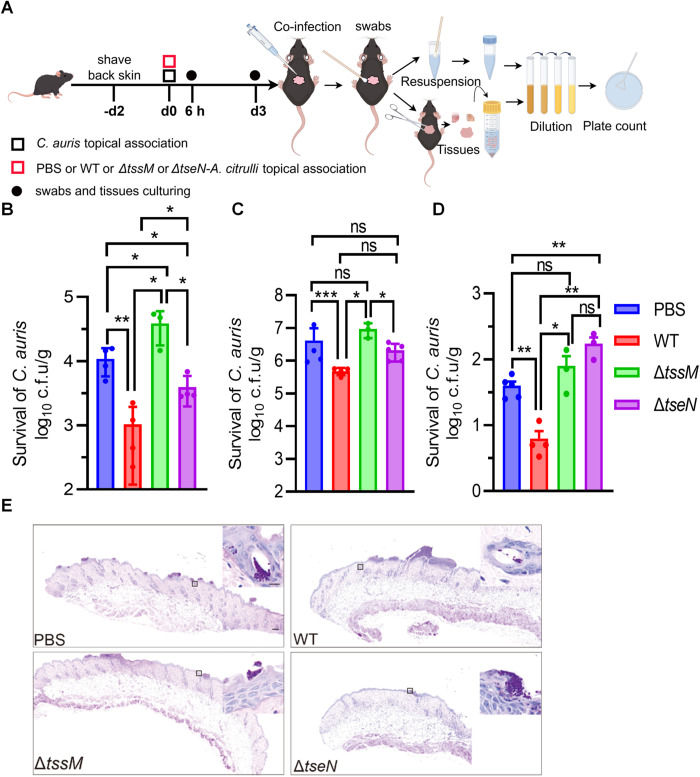
Treatment with *A. citrulli* reduces the survival of *C. auris* in mouse skin infections. **(A)** Schematic representation of skin colonization by *C. auris* (1 × 10^8^ CFUs) with PBS, or *A. citrulli* WT, Δ*tssM*, or Δ*tseN* strains (1 × 10^9^ CFUs) in an *in vivo* assay. Following infection, the viabilities of *C. auris* and *A. citrulli* were assessed at different time points and locations. Specifically, the back-skin surface (**B**) and back-skin tissue (**C**) were sampled after 6 hours, and the ear-skin surface (**D**) was sampled after 3 days. The viable counts were determined by plating on a selective YPD medium for *C. auris* and LB medium for *A. citrulli*. **(E)** Representative histopathological sections of longitudinal dorsal skin from *C. auris*-infected mice inoculated with PBS, or *A. citrulli* WT, Δ*tssM,* or Δ*tseN* strains were collected at 6 hours post-infection and stained with Periodic acid-Schiff (PAS) (scale bar = 100 μm, inset; scale bar = 10 μm, detailed sections). The Magenta color indicates *C. auris* cells in lesion areas. Error bars indicate the standard deviation of at least three biological replicates and statistical significance was calculated using a *t*-test analysis for each group, *p < 0.05, **p < 0.01, ***p < 0.001, ns, not significant.

Given the potent *in vitro* fungicidal activity of the *A. citrulli* T6SS and its effector TseN, we tested the *in vivo* efficacy using a mouse skin infection model. *C. auris* was incubated with wild-type (WT), ∆*tssM*, and ∆*tseN* strains of *A. citrulli* on mouse-injured back skin for 6 hours and ear skin for 3 days, with phosphate-buffered saline (PBS) serving as a control (Fig 4A). Fungal burden was quantified via skin swabbing and tissue homogenization. At 6 hours post-infection in both skin surface and tissue samples, *C. auris* counts were significantly lower in the WT group than the PBS control or the ∆*tssM* group, suggesting that the *A. citrulli* T6SS is effective *in vivo* (Fig 4B–4C). The ∆*tseN* mutant exhibited a significantly attenuated effect relative to the WT (Fig 4B–4C). After 3 days, the WT group exhibited significant clearance of *C. auris*, with fungal abundance reduced by at least 90% relative to the control samples (Fig 4D). These results were corroborated with periodic acid–Schiff (PAS) staining that highlights fungal cells in dark magenta (Fig 4E). These findings underscore the critical role of *A. citrulli* T6SS and TseN in outcompeting *C. auris in vitro* and *in vivo*.

### *A. citrulli* T6SS induces global transcriptome responses in fungal cells

**Fig 5:**
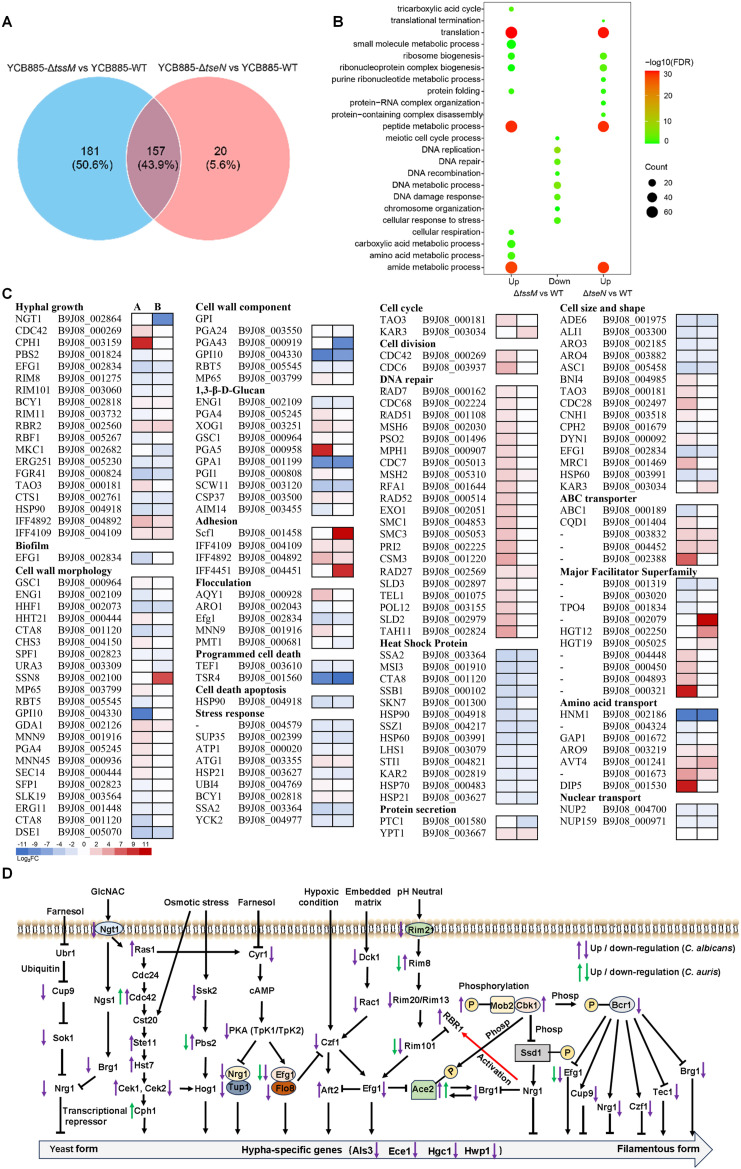
Transcriptomics reveals the roles of T6SS and the effector TseN in *A. citrulli* and fungal interactions. **(A)** Venn diagram showing the differentially expressed genes (*p* < 0.05) from comparisons of YCB885-Δ*tssM* and YCB885-Δ*tseN* relative to YCB885-WT. 177 and 338 fungal genes in *C. auris* were differentially expressed in response to TseN (YCB885-Δ*tseN* vs YCB885-WT) or the T6SS (YCB885-Δ*tssM* vs YCB885-WT), respectively. **(B)** Gene Ontology (GO) analysis from up- or downregulated differentially expressed genes (*p*-value) from comparisons of YCB885-Δ*tssM* to YCB885-WT, and YCB885-Δ*tseN* to YCB885-WT. **(C)** The heat map of Log_2_ fold change of differential genes (DEGs) of *C. auris* triggered by T6SS attack and TseN of *A. citrulli* (*C. auris* co-cultured with wild type, Δ*tssM*, or Δ*tseN-A. citrulli*) according to the RNA-seq data. DEGs were filtered at a cutoff of *p*-value <0.05. The comparison groups are: A, YCB885-WT vs YCB885-∆*tssM* (the differential gene expression in *C. auris* when co-cultured with either the wild-type strain or the ∆*tssM* strain); B, YCB885-WT vs YCB885-∆*tseN* (the differential gene expression in *C. auris* when co-cultured with either the wild-type strain or the ∆*tseN* strain). Abbreviations: WT, *A. citrulli* AAC00-1 wild type; ∆*tssM*, T6SS-null strain; ∆*tseN*, *tseN*-null strain. *Scf1*, *C. auris’s* specific and dominant adhesin called Surface Colonization Factor. Red and blue cells represent the genes that were significantly upregulated or downregulated, respectively. Transcriptomic analysis from RNA-seq reveals that these genes upregulate DNA repair, DNA replication processes, multiple transport systems, hyphae-regulation, and *C. auris* adhesion. Concurrently, there is a downregulation of heat shock proteins, stress response elements, cell growth regulators, and programmed cell death pathways. **(D)** Regulation of filamentous growth in *C. auris* and *C. albicans* by multiple environmental stresses and signal transduction pathways by targeting the promoters of hypha-specific genes. GlcNAc can strongly inhibit the filamentous growth of fungi, and Ngt1 is the GlcNAc-specific transporter gene. Transcriptional changes in *C. auris* and *C. albicans* genes are indicated by green and purple arrows, respectively.

To further understand the fungal cellular responses to the T6SS activities, we performed RNA-seq analysis on *C. auris* YCB885 co-cultured with WT, Δ*tssM*, and Δ*tseN* of *A. citrulli* for 3 hours or *C. albicans* SC5314 co-cultured with WT and Δ*tssM* of *A. citrulli* for 16 hours. Results show that 177 and 338 fungal genes in *C. auris* were differentially expressed responding to TseN (YCB885-Δ*tseN* vs YCB885-WT) or the T6SS (YCB885-Δ*tssM* vs YCB885-WT), respectively (Fig 5A). Notably, there are 157 overlapping genes between the groups, suggesting that TseN accounts for nearly 44% of the T6SS-elicited effects (Fig 5A). Gene ontology (GO) enrichment analysis revealed significant upregulation of DNA-related biological processes in *C. auris*, including DNA repair, replication, recombination, and chromosome organization in *C. auris* (Fig 5B). By contrast, this upregulation was absent when comparing transcriptional profiles of the YCB885-Δ*tseN* vs YCB885-WT group (Fig 5B).

Heatmap analysis further highlighted gene expression changes associated with hyphal growth, cell wall remodeling, DNA repair, transporter systems (ABC transporters, MFS, amino acid transporters), cell fate (e.g., cell cycling, programmed cell death, and stress responses), nuclear transport, and heat shock proteins (HSPs) both in *C. auris* and *C. albicans* (Figs 5C and S6). Hyphal growth in *Candida* is known to be induced by various environmental stresses [33]. In *C. auris* and *C. albicans* co-cultured with T6SS-inactivated and TseN-deficient *A. citrulli*, transcriptional changes were observed in genes regulating hyphal growth (NGT1, CDC42, CPH1, PBS2, EFG1, RIM8, RIM101A, etc.) (Fig 5D). Notably, EFG1 and CPH1, the two pivotal transcription factors for filamentous growth [34], exhibited significant changes (Fig 5C and 5D). Additionally, heat shock proteins HSP90 and HSP70, which inhibit filamentous growth in *C. auris* [35], along with other small heat shock proteins, were downregulated in both T6SS-inactivated and TseN-deficient groups (Figs 5C and S6). Adhesion proteins of the IFF/HYR family, including IFF4109, IFF4892, and IFF4451, were up-regulated in both groups in *C. auris* (Fig 5C). Notably, the *C. auris*-specific adhesion factor SCF1 was up-regulated 500- and 1,000-fold in the *∆tssM* and the *∆tseN* groups, respectively (Fig 5C).

Large-scale transcriptional changes were observed in genes related to biofilm, cell wall components and morphology, and flocculation. These alterations in hyphal growth, adhesion, and cell surface properties likely affect bacterial-fungal interactions and T6SS-mediated fungal killing. The up-regulation of DNA repair genes is also consistent with the nuclease function of TseN.

### Hyphal formation protects fungi from T6SS killing

**Fig 6:**
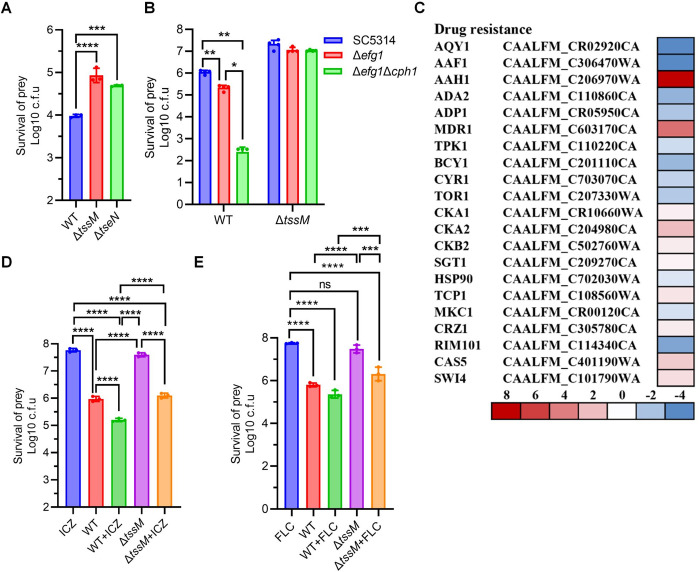
T6SS of *A. citrulli* enhances the killing of drug-resistant *C. albicans* by antifungal drugs. **(A)** Competition assay between *A. citrulli* and *C. albicans*. Competition assay of wild type (WT), the T6SS-null Δ*tssM* mutant, and Δ*tseN* against *C. albicans* on YPD agar plate at 30°C for 3 hours. Survival of prey cells was determined by serial dilutions on selective media. **(B)** Competition assay between *A. citrulli* and hyphae-deficient *C. albicans.* Competition assay of wild type (WT), and T6SS-null Δ*tssM* mutant against a panel of hyphae-deficient *C. albicans* strains (Δ*efg1*/ Δ*efg1* Δ*cph1*) on YPD agar plate at 30°C for 8 hours. **(C)** Regulation of *C. albicans* drug resistance genes by T6SS-mediated attack. Cellular stress response genes associated with azole and polyene resistance are regulated during T6SS challenge. Key regulators include Tpk1, Bcy1, and Cyr1. **(D-E)** *A. citrulli* potentiates azole killing of drug-resistant *C. albicans*. Both the wild-type (WT) strain and the T6SS-deficient Δ*tssM* mutant exhibit a synergistic effect with sub-inhibitory concentrations of **(D)** itraconazole (100 µg/ml) or **(E)** fluconazole (200 µg/ml), improving their efficacy against drug-resistant *C. albicans*. Competition assays on YPD agar at 30°C for 16 hours compared the efficacy of the wild-type (WT) strain and the T6SS-deficient Δ*tssM* mutant against *C. albicans* with and without azole antibiotic. Survival of prey cells was determined by serial dilutions on selective media. Error bars indicate the standard deviation of three biological replicates and statistical significance was calculated using a One–way ANOVA for panel E, and using a two-tailed Student’s *t*-test for panel A, panel B and panel D**.** *p < 0.05, **p < 0.01, ***p < 0.001, ****p < 0.0001.

Considering that *C. albicans*, which forms extensive hyphal structures, exhibited greater resistance to *A. citrulli* T6SS killing compared to *C. auris* (Figs 6A and S7A)*,* and in light of our transcriptome results indicating that hyphal expression is induced in T6SS-mediated response, we hypothesized that hyphal formation affects the T6SS efficacy in killing fungal cells. To test this, we used *C. albicans* strains with deletions of *efg1* and *cph1*, which are known to impair hyphal formation [36–38]. Competition assays showed significantly decreased survival of these hyphae-deficient strains, compared to the wild-type strain (Figs 6B and S7B). These findings suggest that hyphal formation provides protection against the T6SS attack.

### *A. citrulli* enhances azole efficacy against drug-resistant *C. albicans*

Considering the substantial transcriptomic changes in drug resistance-associated gene expression following interaction with *A. citrulli* (Fig 6C), we hypothesized that *A. citrulli* may modulate *C. albicans* drug resistance. To test this, two azole antifungals, fluconazole (a first-line treatment) and itraconazole (a broad-spectrum antifungal), were selected to assess the efficacy of combining *A. citrulli* antifungal activity with antifungal drugs against drug-resistant *C. albicans*. Our results showed that, although the drug-resistant *C. albicans* clinical isolates were resistant to azole treatment alone, *A. citrulli* significantly enhanced the subinhibitory concentrations of azoles-mediated killing of fungal cells.

Interestingly, while neither azoles nor the T6SS-inactive mutant Δ*tssM* exhibited any killing, combined treatment reduced fungal survival by over 90%, suggesting some T6SS-independent effects. Nonetheless, fungal survival was reduced the most when the T6SS was active (Figs 6D–6E and S7C–S7D). The findings suggest the synergy, though not T6SS-specific, highlights the potential of a combined bacterial-drug strategy to control antifungal resistance.

### Distribution of TseN homologs

**Fig 7:**
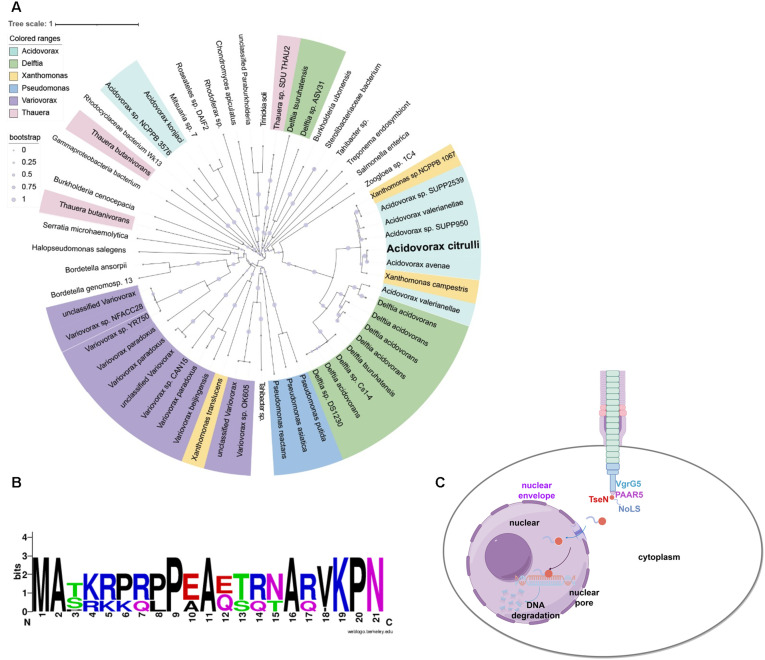
Phylogeny and action of T6SS effector TseN. **(A)** Phylogenetic tree of TseN orthologs from different bacterial species. Protein sequences of TseN orthologs from the indicated bacterial species were obtained from the NCBI database (https://www.ncbi.nlm.nih.gov/). The phylogenetic tree was constructed by MEGA 7.0 using the neighbor-joining method, and displayed by the Interactive Tree of Life (http://itol.embl.de). The clade of TseN in *Acidovorax citrulli* is highlighted in bold black. The scale bar indicates the percentage of divergence (distance). **(B)**The conserved sequence pattern of nucleolar localization sequence (NoLS) observed among TseN homologous proteins derived from the 10 most closely related bacterial species. The consensus sequence pattern was generated with WebLogo based on an alignment of NoLS homologous proteins. In the graph, the height of each letter corresponds to the frequency and conservation level of the amino acid at its respective position. **(C)** Diagram illustrating the mechanism of fungal killing by the T6SS effector TseN. TseN, facilitated by VgrG5 and PAAR5, was secreted into the fungal cell via T6SS. The N-terminal NoLS of TseN mediates its nuclear import. Once localized in the nucleus, TseN degrades genomic DNA, inducing cell death.

Considering the limited understanding of the antifungal functions of the T6SS and the potent antifungal activity exhibited by TseN, we next examined the distribution of TseN homologs. We conducted a phylogenetic analysis of TseN homologs across *Acidovorax*, *Delftia*, *Xanthomonas*, *Pseudomonas*, *Variovorax*, *Thauera*, and other genera (Fig 7A). Notably, the *Acidovorax* TseN did not form a monophyletic group but clustered with *Xanthomonas campestris* and *Xanthomonas sp*. NCPPB 1067. TseN homologues from *Delftia* formed a monophyletic group with *Acidovorax valerianellae*. This distribution likely reflects gene transfer between *Xanthomonas* and the *Acidovorax* genus. Because the NoLS of TseN is the first T6SS effector signal capable of targeting cargo GFP into the fungal nucleus, we performed sequence alignment of TseN NoLS with its homologs to test its prevalence (S8 Fig). Results showed that similar sequences were identified in *Acidovorax*, *Xanthomonas*, and *Delftia* genus, with some sequence variations. The NoLS is featured with enriched basic residues (lysine and arginine) (Fig 7B), consistent with known NoLS sequences in eukaryotic cells [39]. These results suggest the prevalence of antifungal capabilities among T6SS species.

## Discussion

Our findings uncover a potent mechanism of bacterial antagonism against fungal competitors mediated by TseN (Fig 7C). In contrast to the more common reliance on diffusible small molecules such as antibiotics or volatiles to inhibit fungal growth, TseN represents a proteinaceous toxin delivered into the nuclear compartment of fungal cells. Its N-terminal NoLS signal can direct the effector into the nucleus, and its C-terminal DNase domain exhibits no close homologs in existing protein databases, emphasizing that TseN represents a distinct fold with potentially unique catalytic or targeting properties. Although it is unfeasible to capture the process of T6SS-mediated direct delivery of TseN into fungal nucleus due to technical challenges, TseN is likely injected into fungal cells by the T6SS and translocated into the fungal nucleus by the NoLS signal. These features distinguish TseN from previously described T6SS effectors. These findings reveal a highly evolved molecular precision in cross-kingdom competition.

The identification of TseN as a key antifungal effector underscores the versatility of the T6SS. To date, very few T6SS antifungal effectors have been characterized. Phylogenetic analysis reveals that TseN represents a novel class of DNases distributed across multiple bacterial species (Fig 7A), with a unique structure distinguishing it from known DNases. Recently, another antifungal DNase effector, TafE, belonging to the Ntox15 domain family, was identified in *A. baumannii* [17]. GFP-TafE was also shown to localize in the nucleus of yeast cells, although its nuclear localization mechanism remains unclear. To our knowledge, the NoLS sequence of TseN is the first identified and experimentally validated nuclear signal of T6SS effectors. This finding will not only facilitate the discovery of additional anti-eukaryotic T6SS effectors with nuclear targets, but also further our understanding of the mechanisms underlying T6SS-mediated interactions with eukaryotic cells.

The structural characterization of TseN and its immunity protein TsiN provided critical insights into activity regulation. Our crystallographic analysis revealed that TsiN blocks the DNase activity of TseN by sterically hindering its active site, without inducing significant conformational changes (Fig 1H and 1I). This mechanism contrasts with that of known T6SS DNase effectors, such as Tde1 and Tde2, which undergo structural rearrangements upon immunity protein binding [40]. The lack of homology and structural similarity with known DNases supports its representation as a novel DNase family. Future research is warranted to reveal the evolutionary history and functions of its homologs in diverse species.

For unknown reasons, detection of the TseN and other T6SS effectors in the culture medium of AAC00–1 has been unsuccessful in our hands. This might result from effector competition or stability. There are several alternative lines of evidence supporting its delivery. Firstly, deletion of the *tseN* gene severely impaired antifungal activity without affecting antibacterial efficacy [26], suggesting not only the redundancy in antibacterial effectors but also the main role of TseN in antifungal functions. Secondly, expressing TsiN in yeast cells conferred significant protection against the T6SS attacks, strongly supporting that TseN was delivered into yeast cells (Fig 2E). Thirdly, our findings reveal that TseN’s anti-fungal activity requires the coordinated action of its upstream-encoded proteins, including VgrG5, chaperone, and PAAR5, through direct interaction (Fig 2B and 2C). Pull-down assays further reveal that TseN interacts with the C-terminal tail of VgrG5 (Fig 2D). Future work revealing the complex of TseN and its binding partners would be required to elucidate its delivery mechanism.

It remains elusive why some fungal species are more susceptible than others. Transcriptomic analysis of *C. auris* under T6SS attack revealed several fungal defense strategies, including upregulation of DNA repair pathways, cell wall remodeling, and enhanced filamentation and cell adhesion. In turn, T6SS attacks have caused significant side effects for the fungus, such as reduction of programmed cell death and stress response (Fig 5C). While these findings illustrate fungal adaptation to T6SS-mediated stress, the broader implications of these responses for fungal pathogenicity and persistence require further exploration. Our data, showing increased T6SS sensitivity of hyphae defective mutants in *C. albicans*, suggest that filamentous growth offers protection against the T6SS attack. Understanding these survival strategies of fungal pathogens is important for developing small molecules to enhance T6SS efficacy by targeting these survival pathways.

Our *in vivo* experiments demonstrated the efficacy of *A. citrulli* T6SS in reducing *C. auris* colonization in a mouse skin infection model (Fig 4B–4D). Importantly, we did not notice any negative effects resulting from the presence of *A. citrulli* on the mouse skin during preliminary tests (S9 Fig), which is consistent with the fact that *A. citrulli* is a phytobacterium. However, for therapeutic applications, future work will be needed to understand its host immune interactions.

The T6SS of *A. citrulli* exhibits potent activity against diverse fungal pathogens. And yet, such activity is dependent on a single effector TseN. This finding is both mechanistically and ecologically remarkable. It raises questions about the evolutionary pressures that select for such cross-kingdom weapons. Whether other T6SS effectors possess similar nuclear-targeting properties or whether fungi have evolved corresponding countermeasures remains an open and compelling area for future investigation. The wide presence of TseN homologs supports the existence of similar anti-fungal functions in diverse species. Our data suggest that fungi, particularly opportunistic pathogens like *C. auris* and *C. neoformans,* are susceptible targets in environmental niches where T6SS-equipped bacteria, such as *A. citrulli*, persist. This is highly relevant under a One Health framework, where interactions at the interface of plant, animal, and human environments can influence pathogen transmission and colonization potential. The possibility that plant- or soil-associated T6SS-positive bacteria may modulate the dissemination of environmental fungal pathogens with human health relevance is an emerging multidisciplinary topic.

Given the increasing interest in microbiome modulation, our findings also suggest a possible biotechnological or therapeutic use of T6SS and nucleus-targeting effectors for antifungal purposes in agricultural or clinical settings. It can be empowered further by synthetic biology approaches, such as modifying the T6SS to enhance effector delivery specificity and efficiency, engineering effector activities, and secreting other anti-fungal metabolites by chassis cells. Our findings that *A. citrulli* synergized with azole antifungal drugs, substantially enhancing efficacy against drug-resistant *C. albicans*, not only provide solid support for this direction but also demonstrate that drug resistance is relative rather than absolute, suggesting opportunities for synergistic treatment strategies. In summary, TseN defines a structurally and functionally novel class of T6SS effectors with cross-kingdom anti-fungal activity via nuclear disruption. It sets the stage for future investigations into effector-driven microbiome modulation across environmental and health-associated systems.

## Materials and methods

### Ethics statement

All animal experiments were conducted in individual ventilated cages in a pathogen-free animal facility at the Shanghai Institute of Immunity and Infection, Chinese Academy of Sciences. All the procedures were conducted in compliance with the protocol approved by the Institutional Animal Care and Use Committee (IACUC) of the Shanghai Institute of Immunity and Infection, Chinese Academy of Sciences (Permit Number: A2024008).

### Bacterial strains and plasmids

Strains and plasmids are detailed in S2 and S3 Tables, respectively. The strains were cultured in LB, YNB, or YPD media according to established protocols for each species. Antibiotics were administered at specific concentrations: kanamycin (25 µg/ml for bacterial strains, and 100 µg/ml for fungal strains), gentamicin (10 µg/ml), ampicillin (50 µg/ml), and zhongshengmycin (25 × dilutions). TseN and its mutants were cloned into the pBAD-expression vector for toxicity assessment, respectively. Cells harboring different constructs were cultivated in LB with 0.2% (w/v) glucose to inhibit gene expression. TseN-TsiN and TseN_H254A_-TsiN were expressed using the pETDuet plasmid in *E. coli* BL21(DE3) and purified for the DNase activity assay. pETDuet and pET28a were used to express TseN_H254A_^CT^-TsiN and TseN_H254A_^CT^, respectively, in *E. coli* BL21(DE3), for the purpose of obtaining purified proteins for crystallization. *A. citrulli* strains in the animal infection assay were conferred streptomycin and gentamicin resistance. All constructs were confirmed by sequencing. All primers and plasmids are available upon request.

### Bacterial competition assay

For the interspecies competition with fungi, exponential-phase killer cells and stationary-phase prey cells were mixed at a ratio of 10:1 (killer: prey), spotted on YPD or LB plates, and co-incubated at 30°C for the indicated times. Survival of prey and killer cells was quantified by serial dilution and plating on LB containing 10 µg/ml gentamicin and YPD containing 100 µg/ml kanamycin media, respectively.

The initial number of *A. fumigatus* spores, 3 × 10^7^/ml, was quantified using a hemocytometer for spores, and the number of surviving *A. fumigatus* spores after the competition was calculated by performing a spread plate method on a PDA medium.

Preparation of GFP/TsiN-TN124 Prey: Yeast cells were cultured in YNB-TRP (Tryptophan-deficient glucose medium) to an OD_600_ of 1–2. Cells were then transferred to YPR medium (YPD with 2% glucose replaced by 2% raffinose) and adjusted to an OD_600_ of 0.2. The culture was grown to OD_600_ 0.5-0.6 (~6 hours) to deplete intracellular glucose and then induced by adding 2% D-galactose for 3 hours.

Preparation of GFP/TsiN-YCB885 Prey: Yeast cells with an OD_600_ of 2 were inoculated at a 1% dilution in a YPD medium containing 250 ng/ml anhydrotetracycline (aTc) and incubated overnight (12 hours) to reach an OD_600_ of 0.5. Induction was then initiated by adding 1 µg/ml Anhydrotetracycline (aTc), and the culture was shaken for 7 hours, reaching an OD_600_ of 2.5.

### Western blot analysis

Proteins were run on an SDS-PAGE (Sodium Dodecyl sulfate–Polyacrylamide Gel Electrophoresis) gel and then transferred onto a polyvinylidene fluoride (PVDF) membrane (Bio-Rad) through electrophoresis. The membrane was subsequently blocked with a 5% (w/v) nonfat milk solution in TBST buffer (50 mM Tris, 150 mM NaCl, 0.1% (v/v) Tween-20, pH 7.6) at room temperature for 1 hour, followed by sequential treatment with primary antibodies and secondary HRP-conjugated antibodies. Signal detection was achieved using the Clarity Western ECL substrate (Bio-Rad). Monoclonal antibodies targeting epitope tags were procured from ABclonal (Product # AE005 [FLAG] and # AE003 [6His]), Thermo Scientific (Product # 37-7500 [V5]). Secondary antibodies were sourced from ZSGB-Bio (Product # ZB-2305 [mouse]).

### Protein purification and enzymatic assay

TseN and its H254A variant were co-expressed with TsiN in *E. coli* BL21(DE3) using pETDuet-His-SUMO vectors. Cells were cultured in LB medium at 37°C until OD_600_ reached ~0.6, then induced with 0.5 mM IPTG at 16°C for 14 hours. Harvested cells (4,500g, 25 min) were resuspended in lysis buffer (20 mM Tris-HCl pH 8.0, 300 mM NaCl, 10 mM imidazole) and lysed via high-pressure homogenization. Clarified lysates (15,000 g, 20 min) were incubated with Ni-NTA resin (Smart-Lifesciences). Resins were washed with buffer (20 mM Tris-HCl pH 8.0, 300 mM NaCl, 20 mM imidazole) until the effluent showed no detectable protein. His-SUMO-TseN/H254A-TsiN protein complex was eluted with a buffer containing 250 mM imidazole.

Purification of TseN protein under denaturing conditions proceeded as follows: The protein complex was subjected to triplicate dialysis against buffer (20 mM Tris-HCl pH 8.0, 150 mM NaCl) to remove imidazole. Subsequently, the protein sample was denatured in 6 M guanidine hydrochloride (20 mM Tris-HCl pH 8.0, 150 mM NaCl) for 1 hour at ambient temperature, followed by a second Ni-NTA purification. Elution was performed using buffer B (20 mM Tris-HCl pH 8.0, 150 mM NaCl, 6 M guanidine hydrochloride, 250 mM imidazole). Refolding was achieved through stepwise dialysis against decreasing concentrations of guanidine hydrochloride (4 M, 2 M, 1 M, and 0 M) at 4°C.

Purified SUMO protease (20 mM Tris-HCl, 150 mM NaCl, pH 8.0) was added to the refolding His-SUMO-TseN protein solution, followed by a 12-hour dialysis to facilitate His-SUMO tag cleavage and imidazole removal. The resulting solution was incubated with Ni-NTA resin (400 μl) for 1 hour at 4°C with gentle agitation. After centrifugation (10,000 g, 5 min), the supernatant containing the untagged TseN protein was collected and concentrated using a protein ultrafiltration tube (MWCO: molecular weight cutoff = 10 kDa).

Protein activity *in vitro* was detected by incubating with 800 ng plasmid or genomic DNA at 37°C for 10-, 20-, and 30-min. NEB CutSmart buffer (50 mM potassium acetate, 20 mM tris-acetate, 10 mM magnesium acetate, 100 µg/ml bovine serum albumin, pH 7.9) was chosen as the reaction buffer. Purified proteins (100 ng) and 0.5 units DNase I (positive control) were used separately in each reaction.

### Sample preparation for RNA-seq

Wild-type, Δ*tssM*, and Δ*tseN* of AAC00-1 strains were cultured in LB medium to an OD_600_ of 2. Cells were harvested by centrifugation and resuspended to an OD_600_ of 10. *C. auris* and *C. albicans* colonies, grown on YPD agar for 24 hours at 30°C, were suspended in YPD broth. Equal volumes of AAC00-1 and *C. auris* or *C. albicans* suspensions were mixed, spotted on YPD agar, and co-cultured at 30°C for 3 hours or 16 hours, respectively. Co-cultures were harvested, resuspended in LB medium, and pelleted for RNA extraction.

### Protein crystallization, data collection and structure determination

The C-terminus of TseN (TseN^CT^, 160–333aa) and its variant H254A (TseN_H254A_^CT^) were expressed alone or co-expressed with immunity protein TsiN using the pET28a-His-SUMO vectors and pETDuet-His-SUMO vectors in *E. coli* BL21(DE3), respectively. The methods of bacterial culture, protein induction, and purification are the same as described in the enzymatic assay method. Eluted samples were analyzed by SDS-PAGE analysis. The protein is excised from the His-tag in protein dialysis buffer (20 mM Tris-HCl, 150 mM NaCl, pH 8.0) and concentrated using an ultrafiltration tube (MWCO: molecular weight cutoff = 10 kDa) after gel filtration (Superdex-75/ Superdex-200). We use the crystallization reagent kits purchased from Hampton Research for preliminary crystal screening. Crystals obtained from screening are further optimized. X-ray diffraction data were collected at NFPSS BL19U1 beamline and processed using XDS. Data were merged and normalized with Aimless in CCP4 [41]. The crystal structure was determined by molecular replacement with Phaser in the PHENIX suite [42], using the AlphaFold2 predicted structure of the TseN (AlphaFoldDB: AF-A1TP22-F1-v4) as the search model. Iterative refinement and was performed using PHENIX.refine [42] and Coot [43] to obtain a complete structure. Statistical data summarizing data collection and refinement are provided in S1 Table.

### Protein pull-down assay

Genes of interest were cloned into pET28a, pBAD24, and pBBR1MCS vectors for expression. Cells were grown in liquid LB medium with appropriate antibiotics to the exponential phase (OD_600_ ~ 0.4) at 37°C and induced with 0.5 mM IPTG overnight at 20°C for pET28a vectors or 0.2% L-arabinose (L-Ara) for 3 hours at 37°C for pBAD24 vectors. Cells were harvested by centrifugation at 4500g for 10 min, resuspended in lysis buffer (20 mM Tris, pH 8.0, 500 mM NaCl, 50 mM imidazole with protease inhibitor), and lysed by sonication. After cell debris was removed by centrifugation at 15,000 g for 5 min, supernatants were mixed and incubated with Ni-NTA resin at 4°C for 1 hour. The resins were then washed five times with wash buffer (20 mM Tris, pH 8.0, 500 mM NaCl, 50 mM imidazole), and the sample was eluted in elution buffer (20 mM Tris, pH 8.0, 500 mM NaCl, 500 mM imidazole). Input and eluted samples were boiled for 10 min before SDS-PAGE and Western blot analysis.

### Bacterial toxicity assay

Cells harboring different plasmids were grown on LB agar plates with appropriate antibiotics and 0.2% (w/v) glucose at 30°C overnight. Cells were then harvested and resuspended in fresh liquid LB medium and grown to OD_600_ = 1. A series of ten-fold dilutions was plated on LB agar plates containing 0.1% (w/v) L-arabinose or 0.2% (w/v) glucose for induction and repression, respectively. The cells were induced with 0.1% (w/v) L-arabinose for 2 hours. The survival of *E. coli* before and after induction was enumerated by ten-fold dilutions on LB agar plates containing 0.2% (w/v) glucose and appropriate antibiotics. Each experiment was performed at least twice, with one representative experiment shown.

### Bacterial two-hybrid assay

Proteins were fused to the T25 and the T18 domains of the *Bordetella* adenylate cyclase as previously described [44]. Plasmids encoding fusion proteins were co-expressed in the reporter strain BTH101. Single colonies for each transformation were inoculated into 300 μl of LB medium, respectively, and grown for 4 hours at 30°C with shaking. Each culture (3 μl) was spotted onto LB agar plates supplemented with kanamycin, ampicillin, IPTG (0.02 mM), and X-Gal (40 µg/ml). Plates were incubated for 6 hours at 30°C and then 10 hours at room temperature. The experiments were performed in triplicate and a representative result is shown.

### Yeast strain construction and transformation

The *tseN* gene and its mutants (*tseN*_H254A_ and *tseN*^ΔNoLS^) were each fused with sfGFP and cloned into the pRS414 vector (containing a TRP1 selection marker). This vector features a galactose-inducible promoter for regulated expression. The plasmids were introduced into yeast cells via sorbitol electroporation, using either the TN124 (*MAT***a** *leu2 ura3 trp1 pho8*Δ*60 pho13*Δ::*LEU2*) standard strain or a TN124 strain with mCherry-labeled Nab2 mediated by the pRS416 integrative plasmid (containing a URA3 selection marker). Positive clones were selected through multiple screening steps using amino acid-deficient media and kanamycin.

### Yeast toxicity assay

Yeast cells were cultured overnight in tryptophan-deficient YNB minimal medium until an optical density at 600 nm (OD_600_) of 1–2 was reached. The cells were then harvested, washed with glucose-free YNB medium, and resuspended in YPR medium (2% Raffinose replacing glucose in YPD) to an optical density at 600 nm (OD_600_) of 0.2–0.3. The culture was incubated for 5–6 hours, followed by induction with 2% D-galactose for an additional 3 hours. Both pre- and post-induction yeast cells were serially diluted 10-fold, and 3 μl of each dilution was spotted onto YNB plates lacking tryptophan. The plates were incubated at 30°C for 3 days before image acquisition.

### Confocal microscope observation of yeast

pRS414 recombinant plasmids expressing target gene-GFP fusion, or GFP alone, were constructed and transformed into the TN124 strain (or TN124 with mCherry-labeled Nab2). Strains were initially cultured in Tryptophan-deficient (or Tryptophan-Uracil-deficient) -glucose medium overnight, then transferred to YPR medium (2% Raffinose replacing glucose in YPD) at OD_600_ = 0.2. Upon reaching OD_600_ = 0.5-0.6 (~6 hours), the expression was induced with 2% galactose for 3 hours. Cells were harvested (5,000 rpm), washed with YPR medium, and resuspended to an OD_600_ of 20. A 0.5 µl aliquot was placed on a 1% agarose gelatin block in 0.5 × PBS. Imaging was performed using an Olympus high-speed, high-sensitivity laser confocal microscope equipped with the IXplore SpinSR10 super-resolution imaging system with 488 nm and 594 nm lasers for GFP and mCherry visualization, respectively.

### TUNEL assay -DNA damage detection

Sample preparation and fixation: Yeast cell suspension (OD_600_ = 0.6–1.0), induced for 3 hours with 2% D-galactose, was collected (1.5 ml) and fixed in 4% (v/v) formaldehyde at room temperature for 30 minutes. Cells were pelleted by centrifugation and washed three times with 1 × PBS buffer.

Cell Wall Digestion and Permeabilization: Fixed cells were resuspended in Zymolyase 20T solution (50 µg/ml) and incubated at 37°C for 60 min to digest cell walls. Following digestion and centrifugation, the cell pellet was resuspended in permeabilization buffer (0.1% Triton X-100, 0.1% sodium citrate; 100 µl) and incubated on ice for 2 min. Cells were pelleted by centrifugation.

DNase I Treatment: For positive controls (sfGFP-TN124), cells were resuspended in DNase I working solution (1 µl DNase I in 50 µl 1 × reaction buffer; 100 µl total) and incubated at room temperature for 10 min. Negative controls (sfGFP-TN124) and experimental samples (*tseN*/*tseN*_H254A_/*tseN*^ΔNoLS^-sfGFP-TN124) were resuspended in 50 µl 1 × PBS and incubated similarly. All samples were subsequently centrifuged and washed twice with 1 × PBS.

TUNEL Staining: Terminal deoxynucleotidyl transferase dUTP nick-end labeling (TUNEL) was performed using a commercial kit according to the manufacturer’s instructions. Cell pellets were resuspended in TUNEL reaction mixture (containing fluorescein-dUTP and terminal deoxynucleotidyl transferase) and incubated at 37°C for 60 min in the dark. Cells were washed twice with 1 × PBS and resuspended in 1 × PBS to an OD_600_ ~ 10. Aliquots (0.5 µl) were spotted onto agar pads and covered with coverslips.

Fluorescence Imaging: Samples were visualized using a spinning-disk confocal microscope. sfGFP fluorescence was detected using 488 nm excitation. TUNEL signals (fluorescein-dUTP labeling) were imaged using 640 nm excitation.

### Murine model of skin colonization

*In vivo,* assessment of skin colonization of *C. auris* was conducted using an established murine skin model as described previously [45,46]. Groups of female C57BL/6 mice (6–8 weeks old, weighing 18–20 g) were used for the study. Briefly, the dorsal skin of the mice was shaved two days before the topical application, and the back skin was gently scratched with a syringe needle before infection. Yeast cells of *C. auris* (1 × 10^8^ CFUs) or PBS or bacterial cells of *A. citrulli* wild type (WT), Δ*tssM* or Δ*tseN* (1 × 10^9^ CFUs) were topically applied to the shaved dorsal skin and pinna areas of the mice using a Puritan cotton swab. For *A. citrulli* single-skin colonization, individual mice were topically associated with 1 × 10^9^ cells on the back every other day four times. The skin surface was swabbed, and on day 14, skin swabs and skin tissue were taken, weighed, and processed for culturing on a selective LB solid medium. For the interaction of *C. auris* and *A. citrulli*, skin swabs, and tissue samples were collected, weighed, homogenized, serially diluted, and plated onto selective LB and YPD agar plates. *A. citrulli* CFUs and fungal CFUs were determined after incubation at 30°C for 48 hours. The fungal burden in swabs was expressed as CFU/ml, while the fungal burden in skin tissue was expressed as CFUs/g. For histological analysis, mice were euthanized, and their skins were removed and fixed with 4% paraformaldehyde. The paraffin-embedded tissue sections were processed routinely and stained with PAS.

### Bioinformatic and statistical analysis

Statistical analyses were conducted using GraphPad Prism 9.3.0. Significance was assessed via two-tailed Student’s *t*-test, one-way ANOVA, or two-way ANOVA as appropriate.

NoLS prediction website:

NOD: NucleOlar localization sequence Detector (dundee.ac.uk) https://www.compbio.dundee.ac.uk/www-nod/index.jsp

The phylogenetic tree was constructed by the neighbor-joining method, using MEGA 7.0 and displayed by the Interactive Tree of Life (http://itol.embl.de).

The schematic diagrams presented in Figs 4A, 7C, and S9A were created by Figdraw (www.figdraw.com). The associated copyright codes are: TRUPAc496a (Fig 4A), SAYPSe4ed4 (Fig 7C), and SPAYI47e8d (S9 Fig). The full terms of use governing Figdraw can be accessed directly at: https://www.figdraw.com/#/paint_about.

## Supporting information

S1 Fig Effects of *A. citrulli* effector deletion on fungal interactions. (**A-C**) Survival of killer strains during competition assays, with the corresponding prey survival data displayed in Fig 1A-C. (**D-E**) Competition assay of wild type (WT), the T6SS-null Δ*tssM* mutant, and Δ*tseN* against *C. tropicalis* on LB agar for 6 hours at 30°C. Survival of prey and killer cells during competition assays is depicted in panel D and panel E. (**F-G**) Competition assay of wild type (WT), the T6SS-null Δ*tssM* mutant, and Δ*tseN* against *C. glabrata* on LB agar for 3 hours at 30°C. Survival of prey and killer cells during competition assays is depicted in panel F and panel G. (**H-K**) Competition assay of wild type (WT), the T6SS-null Δ*tssM* mutant, and Δ*tseN* against *C. neoformans* (**H**-**I**) *or A. fumigatus* (**J**-**K**) on LB agar for 16 hours at 30°C. The survival of prey (*C. neoformans*) and killer (*A. citrulli*) cells during competition assays is depicted in panels H and I; the survival of prey (*A. fumigatus*) and killer (*A. citrulli*) cells during competition assays is depicted in panels J and K. (**L**) The cellular morphology of *A. fumigatus* after competition with *A. citrulli* was observed by microscopy. For panels A-K, error bars indicate the standard deviation of three biological replicates and statistical significance was calculated using a One–way ANOVA (panels A, D-K) and a two-tailed Student’s *t*-test (panels B-C). **p < 0.01, ***p < 0.001, ****p < 0.0001, ns, not significant. DL, detection limit.
(TIF)

S2 Fig Domain-based multiple sequence alignment of TseN and homologs. (**A**-**D**) TseN was segmented based on predicted conserved domains: (A) residues 1–59, (B) 60–120, (C) 99–159, (D) 160–332. For each segment, we identified the top 39 most conserved homologous proteins through BLASTP searches. Multiple sequence alignments were generated using MUSCLE v3.8 implemented in MEGA 7.0 with default parameters. Resulting alignments were visualized in ESPript 3.0, where identical residues were highlighted in red and experimentally determined secondary structure elements (derived from reference PDB files) were annotated above sequences. Sequence logos below each alignment depict residue conservation; letter height corresponds to amino acid frequency/conservation level. Arrows indicate conserved residues mutated to alanine.
(TIF)

S3 Fig Characterization of the T6SS effector TseN and its immunity protein. (**A**-**B**) The toxic effects of expressing wild-type TseN or its mutant variants from pBAD plasmids in *E. coli* are demonstrated in panel (A), while panel (B) presents the corresponding Western blot analysis. All constructs were cloned on pBAD vectors, and the survival of *E. coli* was enumerated by serial plating on 0.2% arabinose (induction) and 0.2% glucose (repression) plates with 10-fold dilutions. (**C**) Western blot analysis confirmed the expression of truncated TseN variants: TseN^CT^ (200–333 aa) and TseN^CT^ (250–333 aa). (**D**) SDS-PAGE analysis of purified TseN and its catalytic mutant TseN_H254A_ as a quality control for the enzymatic assay presented in Fig 1F. (**E**) DNA degradation by TseN and its mutant TseN_H254A_. Purified 600 ng of genomic DNA of *S. cerevisiae* BY4741 was treated with GFP, DNase I, TseN, and TseN_H254A_ protein. DNA was sampled at the indicated time points and examined by electrophoresis on an agarose gel. For each 5 μl reaction, 0.5 μl of 10 × CutSmart buffer and either 100 ng of TseN or TseN_H254A_ were used. Commercial DNase I (1 unit) was used as a positive control, and GFP protein (100 ng) was used as a negative control. **(F-G)** Western blot analysis showing the expression signals of N-terminal FLAG-tagged full-length TseN (F) and C-terminal 3V5-tagged TsiN (or Aave_2132) (G) in the overexpression strains depicted in Fig 1G. (**H**) Bacterial two-hybrid analysis of TseN-TsiN interaction. TseN and TsiN were fused to adenylate cyclase fragments T25 or T18 and co-expressed in reporter strain BTH101, as indicated. Protein interaction activates cAMP synthesis, resulting in blue color development on LB-X-gal plates. The T6SS transcriptional regulator VasH served as a negative control. (**I**) Purified TseN_H254A_-TsiN complex for protein crystallization. (**J**-**K**) Overall structures of TseN_H254A_^CT^ (J) and TsiN (K). Overall structure of TseN_H254A_^CT^ shown in cartoon (cyan, left), and topology representations with the secondary structural elements labeled (right). Overall structure of TsiN shown in cartoon (magenta, left), and topology representations with the secondary structural elements labeled (right). (**L**) The TseN_H254A_^CT^-TsiN interface analyzed using PDBePISA. The interface is stabilized by 5 salt bridges, 15 hydrogen bonds, and 118 non-covalent contacts. It occludes 1,169.6 Å^2^ and 1,151.8 Å^2^ of solvent-accessible surface area on TseN_H254A_^CT^ and TsiN, respectively, comprising 13.5% and 13.1% of their total ASA. The interface involves 24 amino acid residues from TsiN and 19 residues from four helical regions (α1, α2, α5, and 3_10_) in TseN_H254A_^CT^.
(TIF)

S4 Fig VgrG5, Aave_2128, and PAAR5 are critical for the T6SS-mediated delivery of TseN. (**A**) Survival of killer strains during competition assays, with the corresponding prey survival data displayed in Fig 2B. **(B-E)** Western blot analysis confirms the expression of VgrG5, Aave_2128, PAAR5, and TseN in the complementary strain, as shown in Fig 2B. (**F-G**) Full images of the pull-down analysis in Fig 2C-D. (**H**) Survival of killer strains during competition assays, with the corresponding prey survival data displayed in Fig 2E. Error bars indicate the standard deviation of three biological replicates and statistical significance was calculated using a One-way ANOVA analysis.
(TIF)

S5 Fig Functional characterization of nucleolar localization sequences (NoLS) in the TseN-mediated killing. Survival of killer strains during competition assays, with the corresponding prey survival data displayed in Fig 3F. Error bars indicate the standard deviation of three biological replicates.
(TIF)

S6 Fig Transcriptional changes in *C. albicans* in response to T6SS attack of *A. citrulli.* Heatmap depicting Log2 fold changes of differentially expressed genes (DEGs) in *C. albicans* SC5314 after co-culture with wild-type (WT) *A. citrulli* AAC00–1 or its T6SS-deficient mutant (∆*tssM*), based on RNA-seq data. DEGs were identified using a significance cutoff of adjusted p-value < 0.05. Red cells indicate significantly upregulated genes; blue cells indicate significantly downregulated genes.
(TIF)

S7 Fig Bacterial competition assay between *A. citrull* and *C. albicans* (A-B) Survival of killer strains during competition assays, with the corresponding prey survival data displayed in Fig 6A and 6B respectively. (**C-D**) Survival of killer strains during competition assays, with the corresponding prey survival data displayed in Fig 6D and 6E, respectively. Error bars indicate the standard deviation of three biological replicates.
(TIF)

S8 Fig Multiple sequence alignment of the NoLS in TseN homologs. We selected the ten TseN homologs with the lowest E-values from BLASTP results. These sequences were aligned using ClustalW with default parameters, and the resulting multiple sequence alignment was visualized with ESPript 3.0.
(TIF)

S9 Fig Skin colonization by *A. citrulli* in an *in vivo* assay. (**A**) Schematic representation of skin colonization by *A. citrulli* in an *in vivo* assay. Mice were topically treated with 1 × 10^9^ cells on their backs every other day for a total of four applications (n = 3). (**B**) Survival of *A. citrulli* on the skin surface and in skin tissue. Error bars indicate the standard deviation of three biological replicates. DL, detection limit.
(TIF)

S1 Table Crystallographic data. (DOCX)

S2 Table Strains in this study. (DOCX)

S3 Table Plasmids in this study. (DOCX)

S4 Table Primers in this study. (DOCX)

S1 Data Alignment sequences of TseN homologous proteins. (DOCX)

S2 Data Conserved alignment of NoLS sequences. (DOCX)

S3 Data Homologous sequence of TseN for the establishment of phylogenetic trees. (DOCX)

S4 Data Transcriptome of *C. auris* after co-culture with *A. citrulli.* (XLSX)

S5 Data Transcriptome of *C. albicans* after co-culture with *A. citrulli.* (XLSX)
