# Supplementary figures and images for: A broad-spectrum anti-fungal effector dictates bacterial-fungal interkingdom interactions
Source: PLoS Pathog. 2025 Oct 27;21(10):e1013598. doi: 10.1371/journal.ppat.1013598 (PMC12574953; doi:10.1371/journal.ppat.1013598)

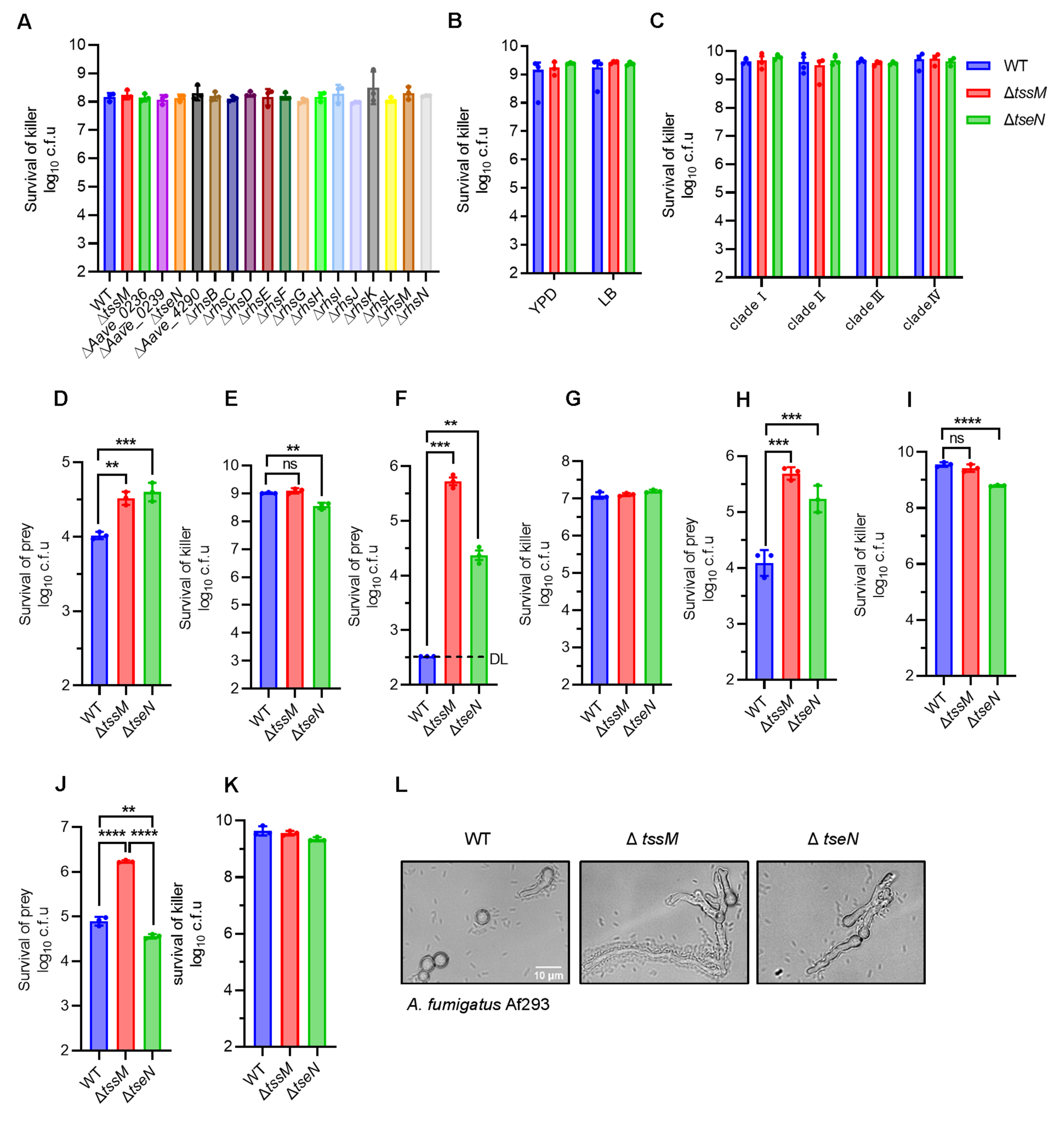

Supplement: S1 Fig — (A-C) Survival of killer strains during competition assays, with the corresponding prey survival data displayed in Fig 1A-C. (D-E) Competition assay of wild type (WT), the T6SS-null ΔtssM mutant, and ΔtseN against C. tropicalis on LB agar for 6 hours at 30°C. Survival of prey and killer cells during competition assays is depicted in panel D and panel E. (F-G) Competition assay of wild type (WT), the T6SS-null ΔtssM mutant, and ΔtseN against C. glabrata on LB agar for 3 hours at 30°C. Survival of prey and killer cells during competition assays is depicted in panel F and panel G. (H-K) Competition assay of wild type (WT), the T6SS-null ΔtssM mutant, and ΔtseN against C. neoformans (H-I) or A. fumigatus (J-K) on LB agar for 16 hours at 30°C. The survival of prey (C. neoformans) and killer (A. citrulli) cells during competition assays is depicted in panels H and I; the survival of prey (A. fumigatus) and killer (A. citrulli) cells during competition assays is depicted in panels J and K. (L) The cellular morphology of A. fumigatus after competition with A. citrulli was observed by microscopy. For panels A-K, error bars indicate the standard deviation of three biological replicates and statistical significance was calculated using a One–way ANOVA (panels A, D-K) and a two-tailed Student’s t-test (panels B-C). **p < 0.01, ***p < 0.001, ****p < 0.0001, ns, not significant. DL, detection limit. (TIF) [file ppat.1013598.s001.tif]

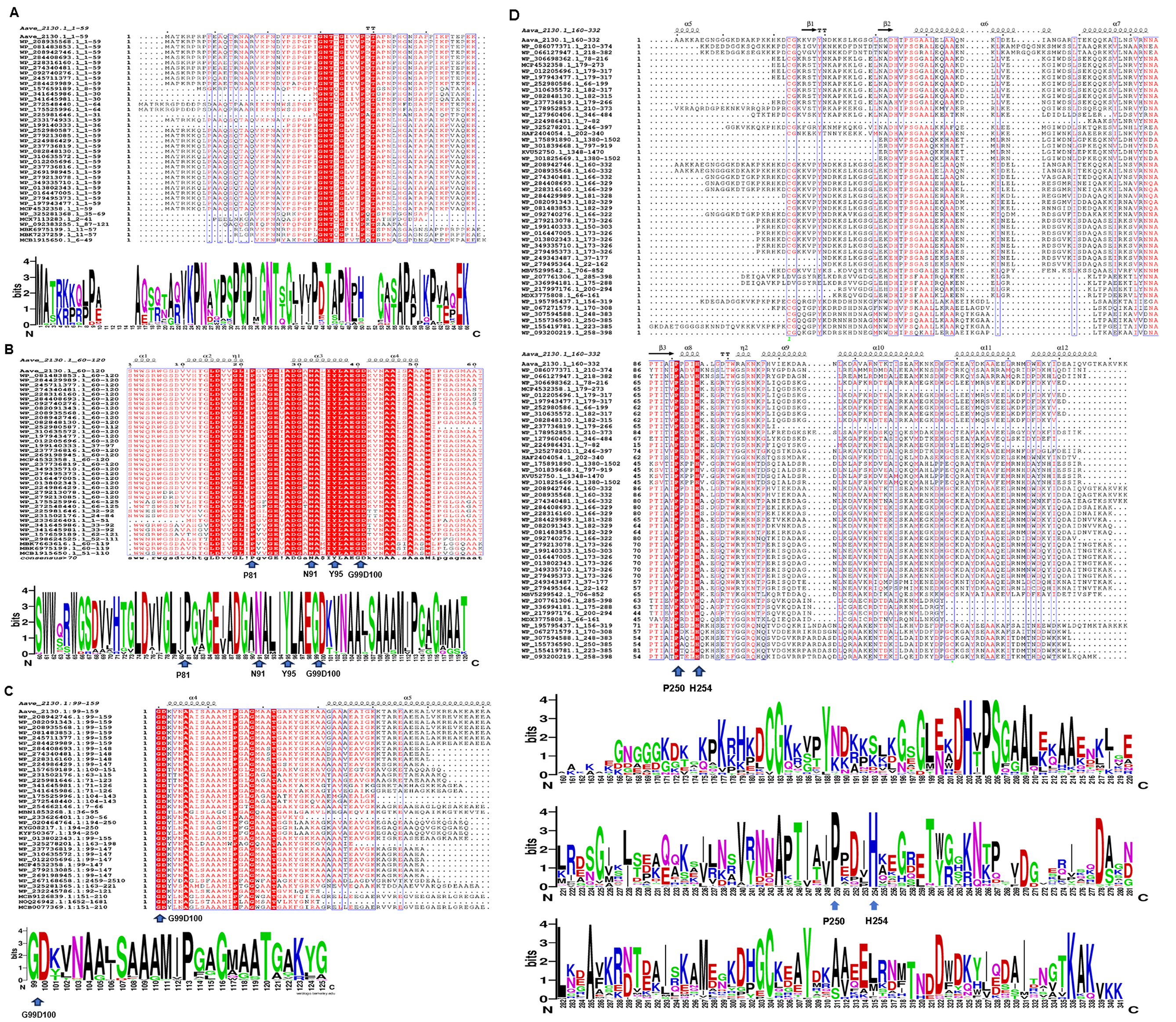

Supplement: S2 Fig — (A-D) TseN was segmented based on predicted conserved domains: (A) residues 1–59, (B) 60–120, (C) 99–159, (D) 160–332. For each segment, we identified the top 39 most conserved homologous proteins through BLASTP searches. Multiple sequence alignments were generated using MUSCLE v3.8 implemented in MEGA 7.0 with default parameters. Resulting alignments were visualized in ESPript 3.0, where identical residues were highlighted in red and experimentally determined secondary structure elements (derived from reference PDB files) were annotated above sequences. Sequence logos below each alignment depict residue conservation; letter height corresponds to amino acid frequency/conservation level. Arrows indicate conserved residues mutated to alanine. (TIF) [file ppat.1013598.s002.tif]

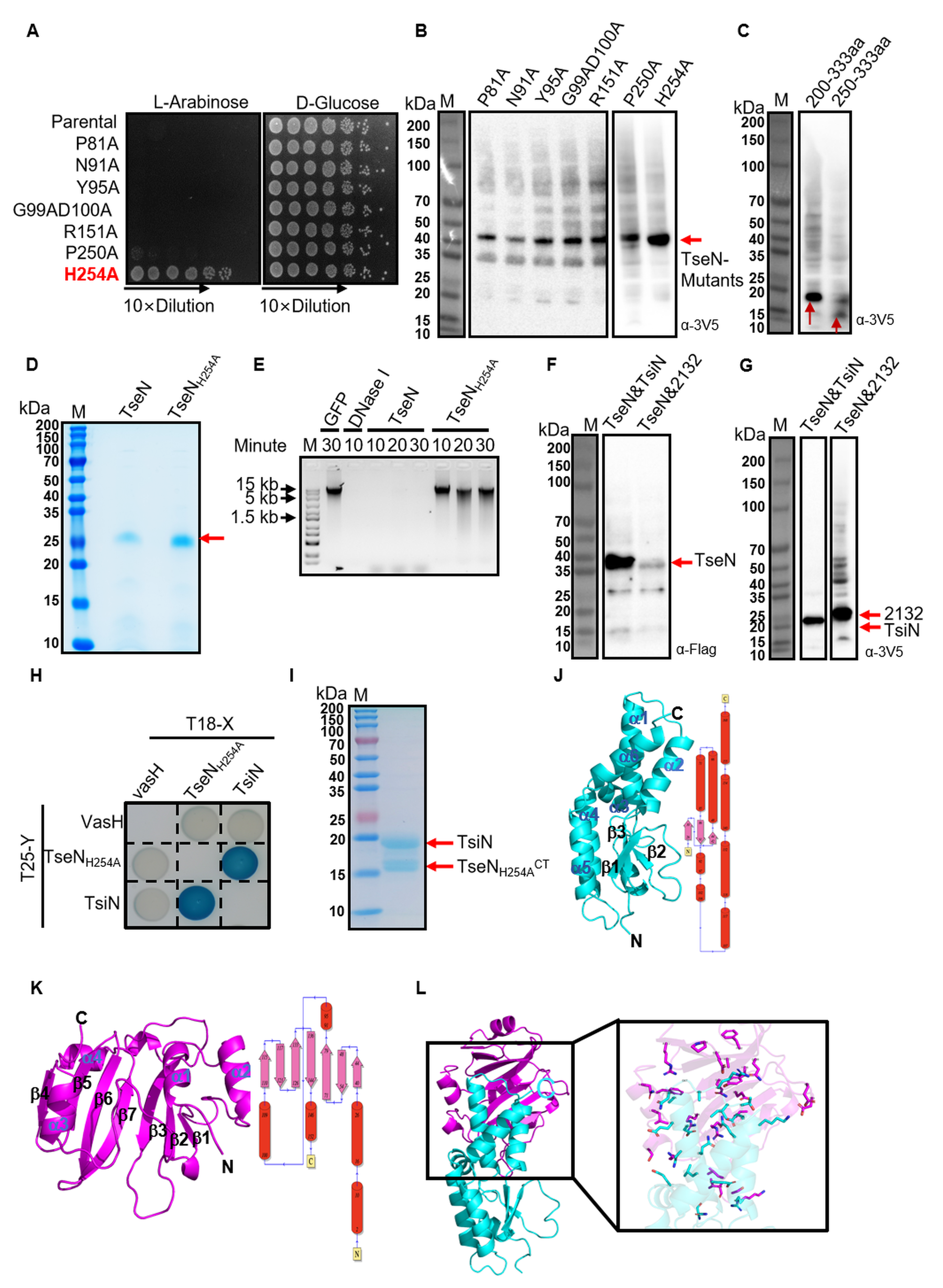

Supplement: S3 Fig — (A-B) The toxic effects of expressing wild-type TseN or its mutant variants from pBAD plasmids in E. coli are demonstrated in panel (A), while panel (B) presents the corresponding Western blot analysis. All constructs were cloned on pBAD vectors, and the survival of E. coli was enumerated by serial plating on 0.2% arabinose (induction) and 0.2% glucose (repression) plates with 10-fold dilutions. (C) Western blot analysis confirmed the expression of truncated TseN variants: TseNCT (200–333 aa) and TseNCT (250–333 aa). (D) SDS-PAGE analysis of purified TseN and its catalytic mutant TseNH254A as a quality control for the enzymatic assay presented in Fig 1F. (E) DNA degradation by TseN and its mutant TseNH254A. Purified 600 ng of genomic DNA of S. cerevisiae BY4741 was treated with GFP, DNase I, TseN, and TseNH254A protein. DNA was sampled at the indicated time points and examined by electrophoresis on an agarose gel. For each 5 μl reaction, 0.5 μl of 10 × CutSmart buffer and either 100 ng of TseN or TseNH254A were used. Commercial DNase I (1 unit) was used as a positive control, and GFP protein (100 ng) was used as a negative control. (F-G) Western blot analysis showing the expression signals of N-terminal FLAG-tagged full-length TseN (F) and C-terminal 3V5-tagged TsiN (or Aave_2132) (G) in the overexpression strains depicted in Fig 1G. (H) Bacterial two-hybrid analysis of TseN-TsiN interaction. TseN and TsiN were fused to adenylate cyclase fragments T25 or T18 and co-expressed in reporter strain BTH101, as indicated. Protein interaction activates cAMP synthesis, resulting in blue color development on LB-X-gal plates. The T6SS transcriptional regulator VasH served as a negative control. (I) Purified TseNH254A-TsiN complex for protein crystallization. (J-K) Overall structures of TseNH254ACT (J) and TsiN (K). Overall structure of TseNH254ACT shown in cartoon (cyan, left), and topology representations with the secondary structural elements labeled (right). Overall structu [file ppat.1013598.s003.tif]

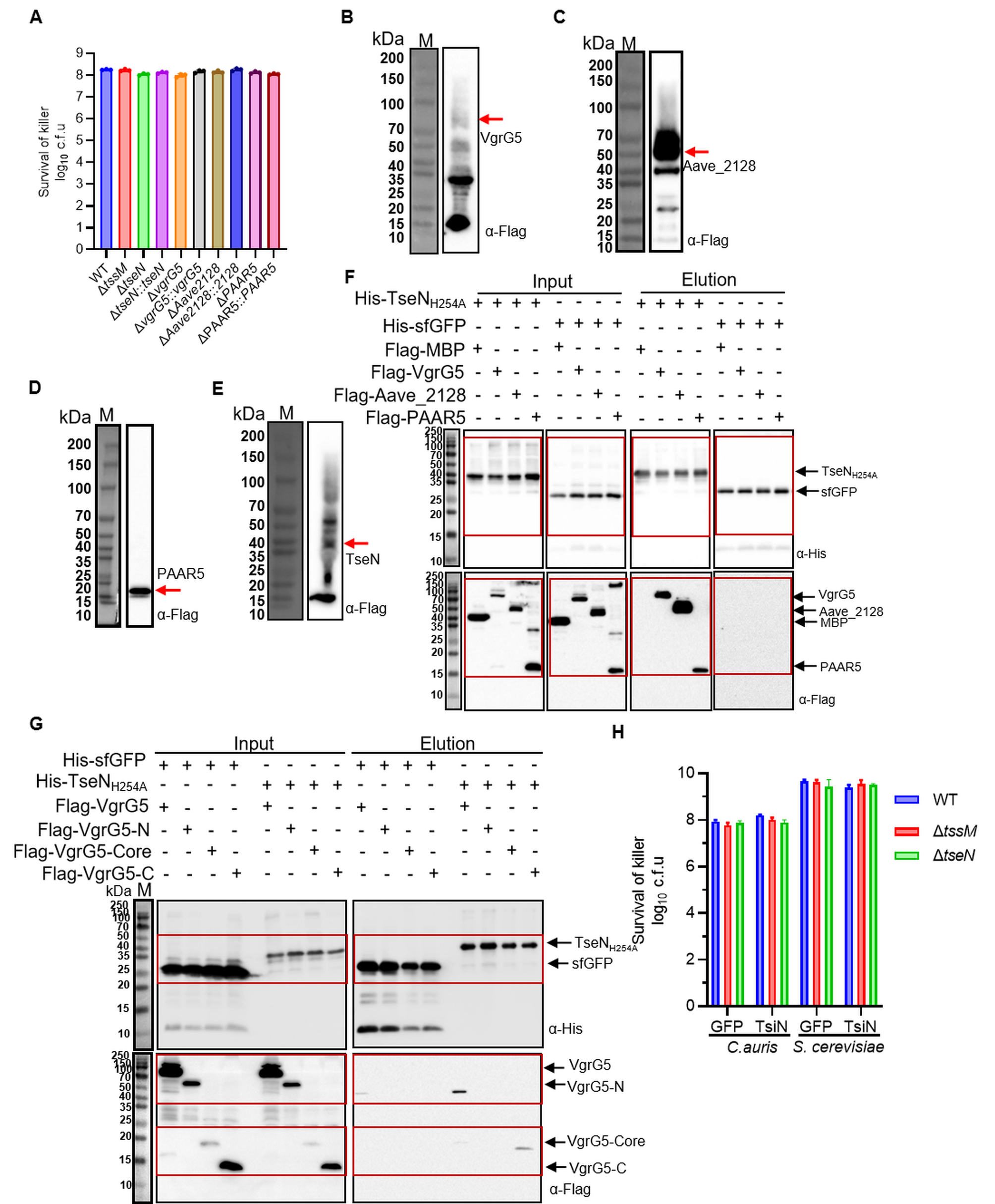

Supplement: S4 Fig — (A) Survival of killer strains during competition assays, with the corresponding prey survival data displayed in Fig 2B. (B-E) Western blot analysis confirms the expression of VgrG5, Aave_2128, PAAR5, and TseN in the complementary strain, as shown in Fig 2B. (F-G) Full images of the pull-down analysis in Fig 2C-D. (H) Survival of killer strains during competition assays, with the corresponding prey survival data displayed in Fig 2E. Error bars indicate the standard deviation of three biological replicates and statistical significance was calculated using a One-way ANOVA analysis. (TIF) [file ppat.1013598.s004.tif]

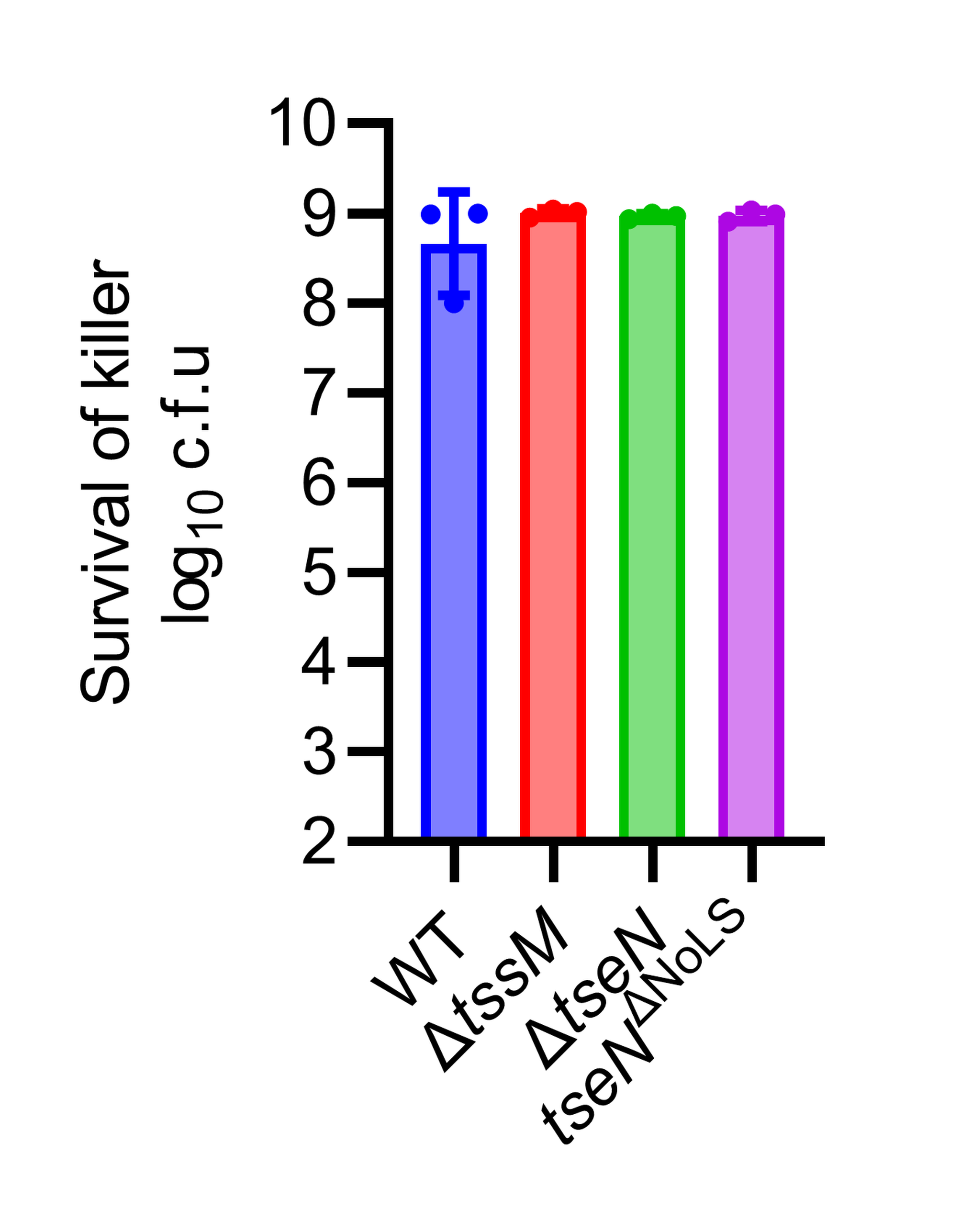

Supplement: S5 Fig — Survival of killer strains during competition assays, with the corresponding prey survival data displayed in Fig 3F. Error bars indicate the standard deviation of three biological replicates. (TIF) [file ppat.1013598.s005.tif]

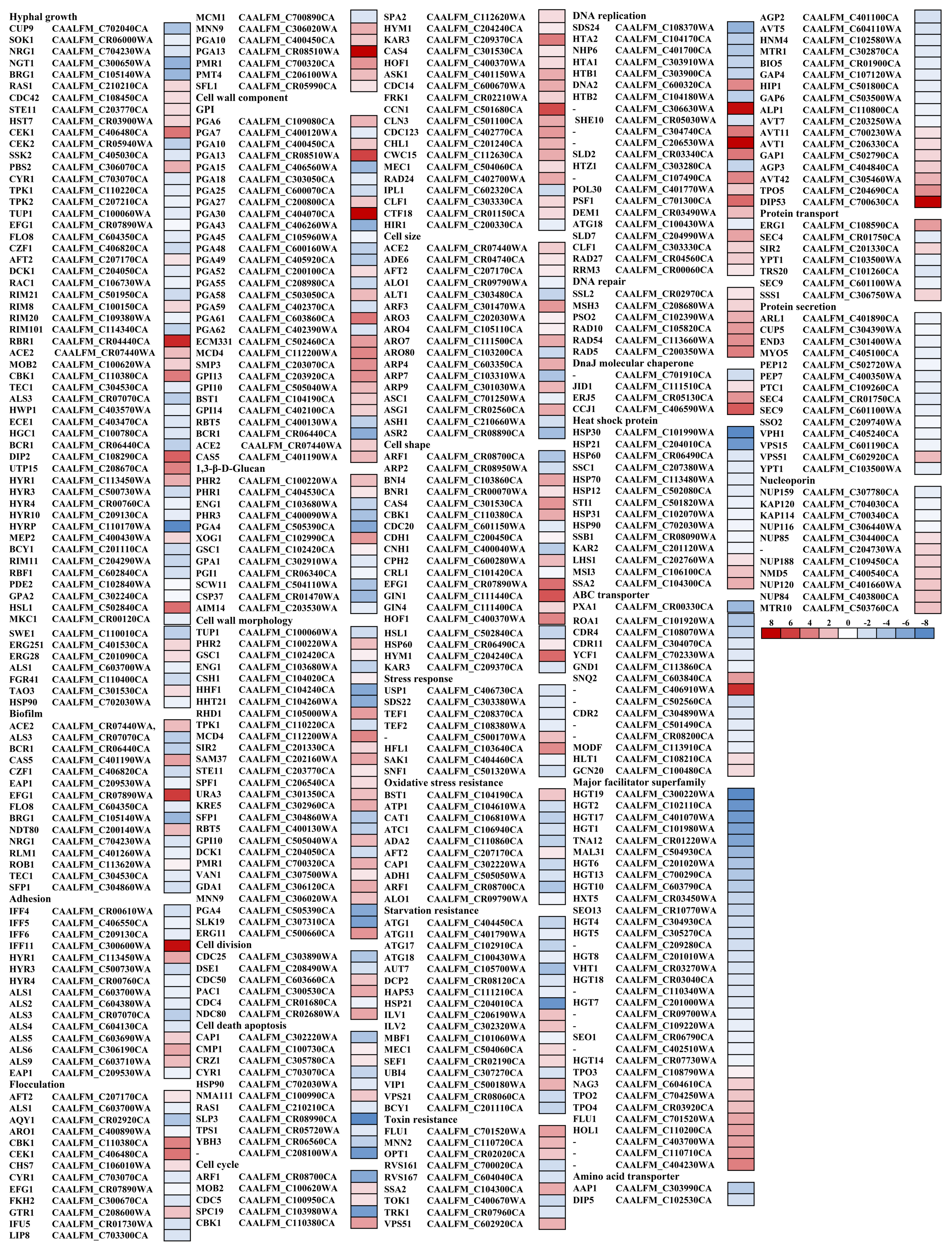

Supplement: S6 Fig — Heatmap depicting Log2 fold changes of differentially expressed genes (DEGs) in C. albicans SC5314 after co-culture with wild-type (WT) A. citrulli AAC00–1 or its T6SS-deficient mutant (∆tssM), based on RNA-seq data. DEGs were identified using a significance cutoff of adjusted p-value < 0.05. Red cells indicate significantly upregulated genes; blue cells indicate significantly downregulated genes. (TIF) [file ppat.1013598.s006.tif]

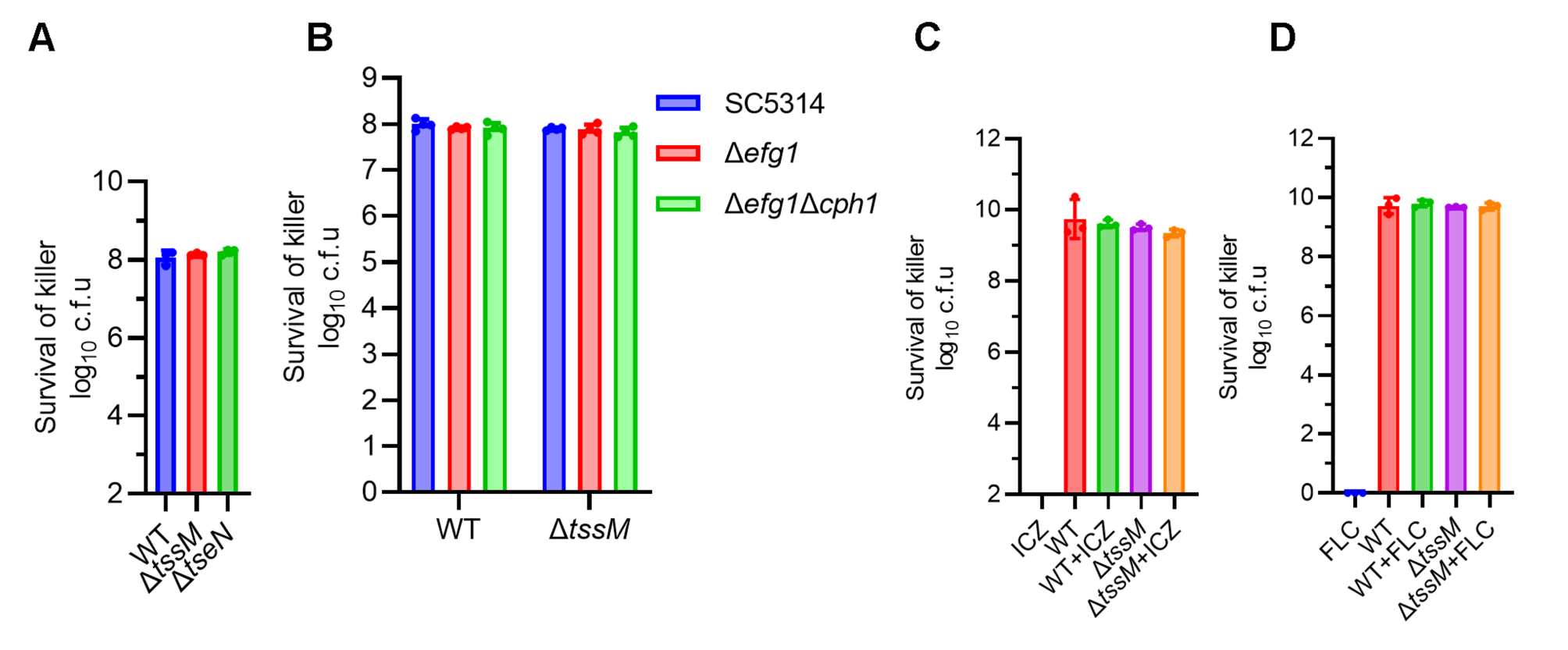

Supplement: S7 Fig — (C-D) Survival of killer strains during competition assays, with the corresponding prey survival data displayed in Fig 6D and 6E, respectively. Error bars indicate the standard deviation of three biological replicates. (TIF) [file ppat.1013598.s007.tif]

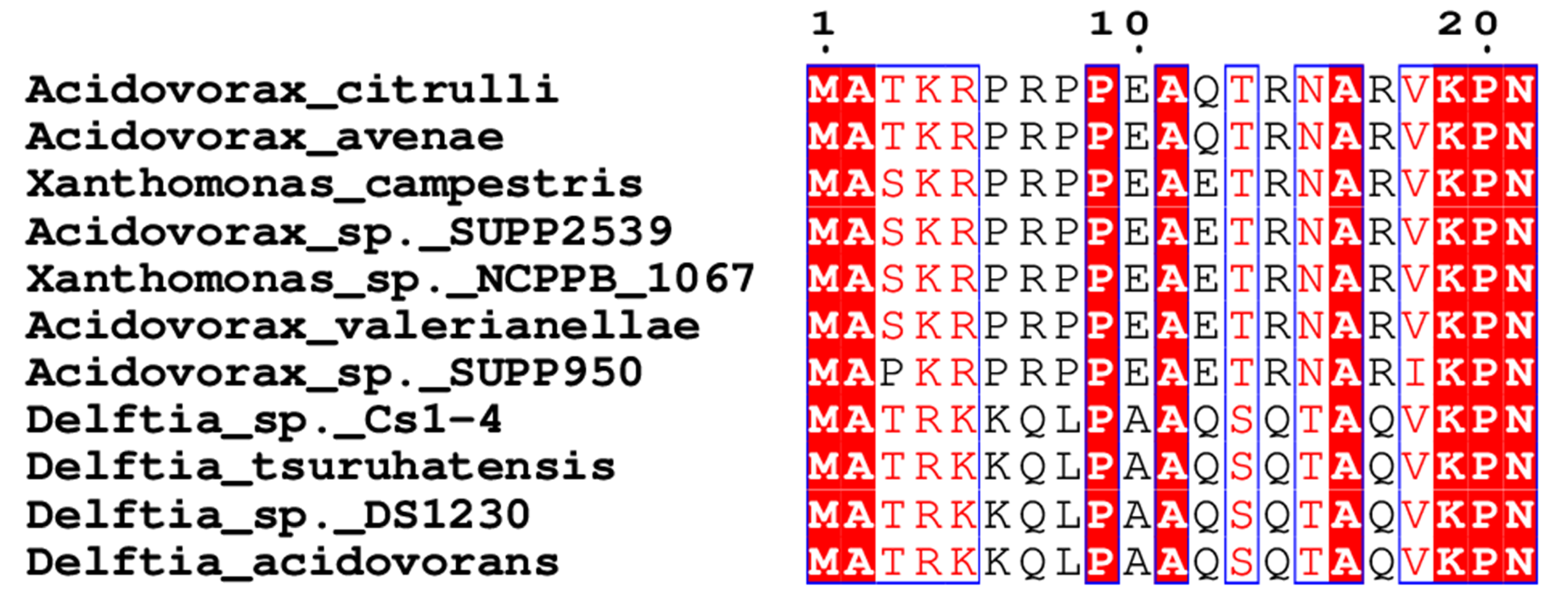

Supplement: S8 Fig — We selected the ten TseN homologs with the lowest E-values from BLASTP results. These sequences were aligned using ClustalW with default parameters, and the resulting multiple sequence alignment was visualized with ESPript 3.0. (TIF) [file ppat.1013598.s008.tif]

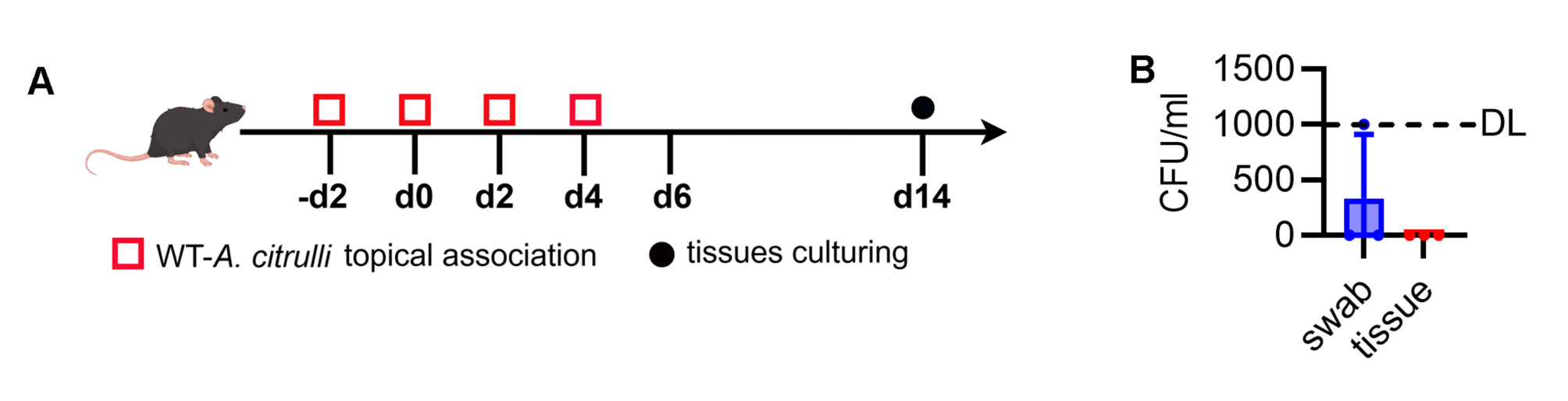

Supplement: S9 Fig — (A) Schematic representation of skin colonization by A. citrulli in an in vivo assay. Mice were topically treated with 1 × 10^9^ cells on their backs every other day for a total of four applications (n = 3). (B) Survival of A. citrulli on the skin surface and in skin tissue. Error bars indicate the standard deviation of three biological replicates. DL, detection limit. (TIF) [file ppat.1013598.s009.tif]
