# Supplementary material for: A broad-spectrum anti-fungal effector dictates bacterial-fungal interkingdom interactions
Source: PLoS Pathog. 2025 Oct 27;21(10):e1013598. doi: 10.1371/journal.ppat.1013598 (PMC12574953; doi:10.1371/journal.ppat.1013598)
Supplement: S1 Table — (DOCX) [file ppat.1013598.s010.docx]

**S1 Table. Crystallographic data**

| TseN_H254A_^CT^-TsiN complex | |
| --- | --- |
| **Data Collection** |  |
| Space group | P 61 2 2 |
| Cell dimension  a, b, c (Å)  α, β, γ (º) | 86.93, 86.93, 170.662  90.000, 90.000, 120.000 |
| Wavelength (Å) | 0.97853 |
| Resolution (Å) | 75.284 - 2.600 |
| No. observed reflections | 451640 |
| No. unique reflections | 12420 |
| R_merge_ | 0.085 |
| (I/σ) | 34.0 |
| Completeness (%) | 100 |
| Multiplicity | 36.4 |
| Wilson B-factor(Å^2^) | 58.047 |
| **Refinement** |  |
| Resolution (Å) | 75.284- 2.600 |
| No. reflections | 12354 |
| R_work_ / R_free_ | 0.2309/0.2846 |
| No. atoms | 2424 |
| Protein | 2419 |
| Water | 5 |
| RMSD |  |
| Bond length (Å) | 0.0090 |
| Bond angles (º) | 1.13 |
| Missing residues |  |
| Chain B | 1-18 |
