# Supplementary material for: A broad-spectrum anti-fungal effector dictates bacterial-fungal interkingdom interactions
Source: PLoS Pathog. 2025 Oct 27;21(10):e1013598. doi: 10.1371/journal.ppat.1013598 (PMC12574953; doi:10.1371/journal.ppat.1013598)
Supplement: S3 Table — (DOCX) [file ppat.1013598.s012.docx]

**S3 Table. Plasmids in this study.**

| Plasmid | Description | Reference |
| --- | --- | --- |
| pEXG2.0 | Suicidal conjugation vector for all chromosomal allelic changes | Lab stock |
| pEXG2.0-Aave_2128 | Suicidal vector to construct inactivating-insertion in Aave_2128 | This study |
| pBAD24kan-2130-3v5 | Arabinose inducible expression of Aave_2130 with a C-terminal 3V5 tag | This study |
| pBAD24kan-2130 H254A -3v5 | Arabinose inducible expression of Aave_2130H254A with a C-terminal 3V5 tag | This study |
| pBAD24kan-2130-2131-3v5 | Arabinose inducible co-expression of Aave_2130 and 2131 with a C-terminal 3V5 tag | This study |
| pBAD24kan-2130-2132-3v5 | Arabinose inducible co-expression of Aave_2130 and 2132 with a C-terminal 3V5 tag | This study |
| pBAD24kan-2130P81A-3v5 | Arabinose inducible expression of Aave_2130P81A with a C-terminal 3V5 tag | This study |
| pBAD24kan-2130N91A-3v5 | Arabinose inducible expression of Aave_2130N91A with a C-terminal 3V5 tag | This study |
| pBAD24kan-Y95A-3v5 | Arabinose inducible expression of Aave_2130Y95A with a C-terminal 3V5 tag | This study |
| pBAD24kan-G99A& D100A-3v5 | Arabinose inducible expression of Aave_2130G99A&D100A with a C-terminal 3V5 tag | This study |
| pBAD24kan-2130R151A-3v5 | Arabinose inducible expression of Aave_2130R151A with a C-terminal 3V5 tag | This study |
| pBAD24kan-2130P250A-3v5 | Arabinose inducible expression of Aave_2130P250A with a C-terminal 3V5 tag | This study |
| pBAD24kan-2130d60-159aa -3v5 | Arabinose inducible expression of Aave_2130 1-59aa and 160-333aa fusion protein with a C-terminal 3V5 tag | This study |
| pBAD24kan-2130d60-99aa-3v5 | Arabinose inducible expression of Aave_2130 1-59aa and 100-333aa fusion protein with a C-terminal 3V5 tag | This study |
| pBAD24kan-2130d2-159aa-3v5 | Arabinose inducible expression of Aave_2130 160-333aa protein with a C-terminal 3V5 tag | This study |
| pBAD24kan-2130d2-59aa 3v5 | Arabinose inducible expression of Aave_2130 60-333aa protein with a C-terminal 3V5 tag | This study |
| pBAD24kan-2130d160-333aa-3v5 | Arabinose inducible expression of Aave_2130 1-159aa protein with a C-terminal 3V5 tag | This study |
| pBAD24kan-2130d120-159aa-3v5 | Arabinose inducible expression of Aave_2130 1-119aa and 160-333aa fusion protein with a C-terminal 3V5 tag | This study |
| pBAD24Kan-2130 200-333aa -3v5 | Arabinose inducible expression of Aave_2130 200-333aa protein with a C-terminal 3V5 tag | This study |
| pBAD24Kan-2130 250-333aa -3v5 | Arabinose inducible expression of Aave_2130 250-333aa protein with a C-terminal 3V5 tag | This study |
| pBBR1MCS5-His-Aave_2127 | Constitutive expression of His-Aave_2127 | This study |
| pBBR1MCS-2 -Aave_2127-Flag | Constitutive expression of Aave_2127-Flag | This study |
| pBBR1MCS-2 -Aave_2128-Flag | Constitutive expression of Aave_2128-Flag | This study |
| pBBR1MCS-2 -Aave_2129-Flag | Constitutive expression of PAAR5-Flag | This study |
| pBBR1MCS-5 -Flag-2130-2131 | Constitutive expression of Flag-tseN-tsiN | This study |
| pRS414 | A shuttle vector with TRP1 selection in *Saccharomyces cerevisiae* TN124 | Lab stock |
| pRS414-sfGFP | Galactose-inducible expression of sfGFP in TN124 | This study |
| pRS414-2130-sfGFP | Galactose-inducible expression of Aave_2130-sfGFP fusion protein in TN124 | This study |
| pRS414-2130H254A-sfGFP | Galactose-inducible expression of Aave_ 2130H254A-sfGFP fusion protein in TN124 | This study |
| pRS414-2130^ΔNoLS^-sfGFP | Galactose-inducible expression of Aave_ 2130^ΔNoLS^-sfGFP fusion protein in TN124 | This study |
| RS414-2130 1-10aa-sfGFP | Galactose-inducible fusion expression of Aave_2130 1-10aa fragment and sfGFP in TN124 | This study |
| RS414- 2130 1-21aa-sfGFP | Galactose-inducible fusion expression of Aave_2130 1-21aa fragment and sfGFP in TN124 | This study |
| RS414- 2130 11-21aa-sfGFP | Galactose-inducible fusion expression of Aave_2130 11-21aa fragment and sfGFP in TN124 | This study |
| RS414-2130 166-193aa sfGFP | Galactose-inducible fusion expression of Aave_2130 166-193aa fragment and sfGFP in TN124 | This study |
| RS414-2130 166-180aa-sfGFP | Galactose-inducible fusion expression of Aave_2130 166-180aa fragment and sfGFP in TN124 | This study |
| RS414-2130 180-193aa-sfGFP | Galactose-inducible fusion expression of Aave_2130 180-193aa fragment and sfGFP in TN124 | This study |
| RS414-GAL-NoLS^1-21aa^-2131 | Galactose-inducible expression of 2131 in TN124 | This study |
| pKNT25-2130H254A | IPTG inducible expression of Aave_2130H254A for bacterial two-hybrid analysis | This study |
| pKNT25-vasH | IPTG inducible expression of VasH for bacterial two-hybrid analysis | This study |
| pCH363N-2131 | IPTG inducible expression of Aave_2131 for bacterial two-hybrid analysis | This study |
| pCH363N-2130H254A | IPTG inducible expression of Aave_2130H254A for bacterial two-hybrid analysis | This study |
| pCH363N-vasH | IPTG inducible expression of VasH for bacterial two-hybrid analysis | This study |
| pET28a-Flag-2130H254A | IPTG inducible expression of Aave_2130H254A with an N-terminal Flag tag | This study |
| pET28a-2130H254A-His | IPTG inducible expression of Aave_2130H254A with an N-terminal His tag | This study |
| pET28a-His-sfGFP | IPTG inducible expression of sfGFP with an N-terminal His tag | This study |
| pBAD24-Flag-2127 | Arabinose inducible expression of Aave_2127 with an N-terminal Flag tag | This study |
| pBAD24-Flag-2127N-term | Arabinose inducible expression of Aave_127N-term with an N-terminal Flag tag | This study |
| pBAD24-Flag-2127C-term | Arabinose inducible expression of Aave_2127C-term with an N-terminal Flag tag | This study |
| pBAD24-Flag-2127Core | Arabinose inducible expression of Aave_2127Core with an N-terminal Flag tag | This study |
| pBAD24-Flag-MBP | Arabinose inducible expression of MBP with an N-terminal Flag tag | Lab stock |
| pBBR1MCS2-Flag-Aave_2128 | Constitutive expression of Aave_2128 with an N-terminal Flag tag | This study |
| pET28a-Flag-2129 | IPTG inducible expression of Aave_2129 with an N-terminal Flag tag | This study |
| pETDuet2-SUMO-N-His-2130-b-2131 | IPTG inducible co-expression of Aave_2130 with an N-terminal His-SUMO tag and Aave_2131 |  |
| pETDuet2-SUMO-N-His-2130 H254A-b-2131 | IPTG inducible co-expression of Aave_2130H254A with an N-terminal His-SUMO tag and Aave_2131 | This study |
| pETDuet-His-SUMO-2130H254A 160-333aa-2131 | IPTG inducible co-expression of Aave_2130H254A 160-333aa fragment with an N-terminal His-SUMO tag and Aave_2131 | This study |

**S4 Table. Primers in this study.**

| **Primer name** | **Sequence (forward/reverse)** | **Description** |
| --- | --- | --- |
| 2127-KO1 | taccgaattcgagctcgagcgtcaggagggagccaatcagag | For deleting the *Aave_2127* gene, located upstream of the *Aave_2127* gene |
| 2127-KO2 | tgtgcttggtcagctgctctcccagggg |  |
| 2127-KO3 | agagcagctgaccaagcacattgggctgg | For deleting the *Aave_2127* gene, located downstream of the *Aave_2127* gene |
| 2127-KO4 | ctgcaggtcgactctgagatctcagtagcggtagtc |  |
| 2127-KO5 | ctgatttgctgcgttgcgag | For confirming the deletion of the *Aave_2127* mutant |
| 2127-KO6 | caccttggggatagtcgatctgc |  |
| 2128-KO1 | acacattatacgagccggaagataacgccgcgaacatgcca | For deleting the *Aave_2128* gene, located upstream of the *Aave_2128* gene |
| 2128-KO2 | tcattggccatggctcgatggttgtgccatagcgtca |  |
| 2128-KO3 | atggcacaaccatcgagccatggccaatgaaaacattgt | For deleting the *Aave_2128* gene, located downstream of the *Aave_2128* gene |
| 2128-KO4 | gcgcacccgtggaaattaattgtccttttccagaccgctaccc |  |
| 2128-KO5 | tcatcgtggacttcctgaacgg | For confirming the deletion of the *Aave_2128* mutant |
| 2128-KO6 | tagttccaaagccgctcctg |  |
| 2129-KO1 | acacattatacgagccggaagaacactgcaactgaccgagc | For deleting the *Aave_2129* gene, located upstream of the *Aave_2129* gene |
| 2129-KO2 | gcttcttccagttttcattggccatggctac |  |
| 2129-KO3 | caatgaaaactggaagaagccgtagcatcc | For deleting the *Aave_2129* gene, located downstream of the *Aave_2129* gene |
| 2129-KO4 | gcgcacccgtggaaattaattgtcgttgtaaggaacctttttgccac |  |
| 2129-KO5 | tgcgatgtgctgctggtgaacg | For deleting the *Aave_2129* gene, located upstream of the *Aave_2129* gene |
| 2129-KO6 | tgcctctttgcatccgtggt |  |
| 2130-KO1 | taccgaattcgagctcgagcgcagatagaggcattcgagatgc | For deleting the *Aave_2130* gene, located upstream of the *Aave_2130* gene |
| 2130-KO2 | ctggattgcttcatctttgacacgcgcgttgc |  |
| 2130-KO3 | cgtgtcaaagatgaagcaatccaggtaggcac | For deleting the *Aave_2130* gene, located downstream of the *Aave_2130* gene |
| 2130-KO4 | ctgcaggtcgactctgagatctcacaaaccatgtttgcgcttg |  |
| 2130-KO5 | acgaagcaggtttaagcaacatgtc | For confirming the deletion of the *Aave_2130* mutant |
| 2130-KO6 | tacgttctcggccgttgatg |  |
| NOLS 1-21aa-KO1 | cacattatacgagccggaagaacggtcagcgtgactatcc | For deleting the NOLS ^1-21aa^ in *Aave_2130* gene, located upstream of the NOLS ^1-21aa^ |
| NOLS 1-21aa-KO2 | aggggtagtccatgggatgctacggcttct |  |
| NOLS 1-21aa-KO3 | tagcatcccatggactacccctcaccgggg | For deleting the NOLS ^1-21aa^ in *Aave_2130* gene, located downstream of the NOLS ^1-21aa^ |
| NOLS 1-21aa-KO4 | gcgcacccgtggaaattaattgtcgttgtaaggaacctttttgccac |  |
| NOLS 1-21aa-KO5 | ctacgttctcggccgttgat | For confirming the deletion of the NOLS ^1-21aa^ mutant |
| NOLS ^1-21aa^-KO6 | gcttttctgtaatcctggcacc |  |
| tssM-KO1 | taccgaattcgagctcgagcggcttcgagggacgctatca | For deleting the *tssM* gene, located upstream of the *tssM* gene |
| tssM-KO2 | gaagcgcagcttcgtgaaccggttgaagaagaatg |  |
| tssM-KO3 | gttcacgaagctgcgcttcgaggtcac | For deleting the *tssM* gene, located downstream of the *tssM* gene |
| tssM-KO4 | ctgcaggtcgactctgagatctctccgcgtcgtaggtgagatc |  |
| tssM-KO5 | catcgagctcatgtacttctgcc | For confirming the deletion of the *tssM* mutant |
| tssM-KO6 | catgaagtggtcgccatcct |  |
| 2130-V5-f | ggtaaacctattcctaatcctctcctt | pBAD24-2130-3V5 plasmid construction for toxicity assay |
| 2130-V5-r | ggtacctcctgctagcccaaa |  |
| 2130 1-159aa-3V5-f | cgaagaggctggtaaacctattcctaatcctctcc | pBAD24-2130 1-159aa- 3V5 plasmid construction for toxicity assay |
| 2130 1-159aa-3V5-r | taggtttaccagcctcttcggcttccttgg |  |
| 2130 160-333aa-3V5-f | catggctgccgcggccaagaaggcggaaggcaat | pBAD24-2130 160-333aa-3V5 plasmid construction for toxicity assay |
| 2130 160-333aa-3V5-r | tcttggccgcggcagccatgccagcgccagggatcat |  |
| 2130 200-333aa-3V5-f | ttgggctagcaggaggtaccatggaaaaggaccacacgcc | pBAD24-2130 200-333aa-3V5 plasmid construction for toxicity assay |
| 2130 200-333aa-3V5-r | ggtacctcctgctagcccaaa |  |
| 2130 250-333aa-3V5-f | ttgggctagcaggaggtaccatgccgcctgacatacatgc | pBAD24-2130 250-333aa-3V5 plasmid construction for toxicity assay |
| 2130 250-333aa-3V5-r | ggtacctcctgctagcccaaa |  |
| 2130 P81A-3V5-f | ggctgatcGcgggtgtcggcgagatcgcggatggcgcaaa | pBAD24-2130P81A-3V5 plasmid construction for toxicity assay |
| 2130 P81A-3V5-r | ctcgccgacacccgCgatcagcccgacgacatccagcccg |  |
| 2130 N91A-3V5-f | atcgcggatggcgcaGCCgcgctgatctatctg | pBAD24-2130N91A-3V5 plasmid construction for toxicity assay |
| 2130 N91A-3V5-r | cagatagatcagcgcgGCtgcgccatccgcgatctcgccgaca |  |
| 2130 Y95A3V5-f | atcgcggatggcgcaGCcgcgctgatctatctggcggagggcga | pBAD24-2130Y95A-3V5 plasmid construction for toxicity assay |
| 2130 Y95A-3V5-r | tcgccctccgccagaGCgatcagcgcgtttgcgccatccgcgat |  |
| 2130 G99AD100A-3V5-f | ctggcggaggCcgCtaaggtcaacgccgccatcagcgct | pBAD24-2130G99AD100A-3V5 plasmid construction for toxicity assay |
| 2130 G99AD100A-3V5-r | gttgaccttaGcgGcctccgccagatagatcagcgcgtt |  |
| 2130 R151A-3V5-f | cgctggtgaaaGCggaggccaaggaagccgaagaggctg | pBAD24-2130R151A-3V5 plasmid construction for toxicity assay |
| 2130 R151A-3V5-r | ttggcctccGCtttcaccagcgcctcttccgcctc |  |
| 2130 P250A-3V5-f | cctacaatagctattGcgcctgacatacatgct | pBAD24-2130P250A-3V5 plasmid construction for toxicity assay |
| 2130 P250A-3V5-r | tatgtcaggcgCaatagctattgtaggcgcattatttctaacgg |  |
| 2130 H254A-3V5-f | attccgcctgacataGCtgctctaggcgatacttggcg | pBAD24-2130H254A-3V5 plasmid construction for toxicity assay |
| 2130 H254A-3V5-r | agtatcgcctagagcaGCtatgtcaggcggaatagctattgta |  |
| 2130-2131-3V5-f | ttgggctagcaggaggtaccatggcgacgaagagacct | pBAD24-2130-2131-3V5 plasmid construction for toxicity assay |
| 2130-2131-3V5-r | ggattaggaataggtttacctggacaaatattattctttcgaaacag |  |
| 2130-2132-3V5-f | atgaatgcaatagctctgattgattttttaggacac | pBAD24-2130-2132-3V5 plasmid construction for toxicity assay |
| 2130-2132-3V5-r | atcagagctattgcattcattttttcacctttgctt |  |
| pCH363-2131-f | cctgcaggtcgactctagagatgaatgcaattgatttgat | pCH363-2131 plasmid construction for Bacterial two-hybrid assay |
| pCH363-2131-r | gaattcgagctcggtaccgctggacaaatattattctttc |  |
| pCH363-2130H254A-f | cctgcaggtcgactctagagatggcgacgaagagacct | pCH363-2130H254A plasmid construction for Bacterial two-hybrid assay |
| pCH363-2130H254A-r | gaattcgagctcggtaccgcttttttcacctttgcttttgtgcc |  |
| pKNT25N-2131-f | caaagcttgcatgctctagaatgaatgcaattgatttgat | pKNT25N-2131 plasmid construction for Bacterial two-hybrid assay |
| pKNT25N -2131-r | atcgatgggaattcggtacctcatggacaaatattattc |  |
| pKNT25N -2130H254A-f | caaagcttgcatgctctagaatggcgacgaagagacct | pKNT25N-2130H254A plasmid construction for Bacterial two-hybrid assay |
| pKNT25N -2130H254A-r | atcgatgggaattcggtacctcattttttcacctttgctt |  |
| His-SUMO-2130/H254A-f | tggtggtggatccggtaccatggcgacgaagagacctc | pETDuet-His-SUMO-2130/H254A-2131  plasmid construction for protein purification |
| His-SUMO-2130/H254A-r | ttaagcattatgcggccgctcattttttcacctttgct |  |
| His-SUMO-2131-f | cgatcgctgacgtcggtaccatgaatgcaattgatttgat |  |
| His-SUMO-2131-r | ggtttaccaccagactcgagtcatggacaaatattattct |  |
| His-SUMO-2130_H254A_^CT^-f | gtggtggatccggtaccatggcggccaagaaggcggaagg | pETDuet-His-SUMO-2130_H254A_^CT^ -2131 plasmid construction for protein purification |
| His-SUMO-2130_H254A_^CT^-r | ttaagcattatgcggccgctcattttttcacctttgct |  |
| pBBR1MCS2-Flag-2128-f | acaaggacgacgatgacaagatggcacaaccatcgcacg | pBBR1MCS2-Flag-2128 plasmid construction for pull-down assay |
| pBBR1MCS2-Flag-2128-r | cttgtcatcgtcgtccttgtaatccatggtttcctcctgtgtgaaattgt |  |
| pET28a-flag-2129-f | atggattacaaggacgacgatgacaagatggccaatgaaaacattgtc | pET28a-flag-2129 plasmid construction for pull-down assay |
| pET28a -flag-2129-r | cagcttcctttcgggctttgctacggcttcttccagttcatc |  |
| pBAD24-Flag-2127N-term-f | gggctagcaggaggtaccatggattacaaggacgacgatgac | pBAD24-Flag-2127N-term plasmid construction for pull-down assay |
| pBAD24-Flag-2127N-term-r | aacagccaagcttttattaccacagctgctccgcgc |  |
| pBAD24-Flag-2127Core-f | acaaggacgacgatgacaagatgctgcatgcgcagaagg | pBBR1MCS2-Flag-2128 plasmid construction for pull-down assay |
| pBAD24-Flag-2127Core-r | aaacagccaagcttttattaactgccgaccttgatcgacc |  |
| pBAD24-Flag-2127C-term-f | acaaggacgacgatgacaagatgttcaagaccgagaccat | pBAD24-Flag-2127Core plasmid construction for pull-down assay |
| pBAD24-Flag-2127C-term-r | aaacagccaagcttttattaattcaggtcgatgcgcttgg |  |
| pBAD24-Flag-2127-f | gggctagcaggaggtaccatggattacaaggacgacgatg | pBAD24-Flag-2127C plasmid construction for pull-down assay |
| pBAD24-Flag-2127-r | aacagccaagcttttattaattcaggtcgatgcgcttgga |  |
| 2130/H254A-linker-f | tcaaggagtctagaaagcttggatccatggcgacgaagagacct | Amplification of 2130/H254A for Gibson Assembly with sfGFP  Amplification of 2130/H254A for Gibson Assembly with sfGFP |
| 2130/H254A-linker-r | ctgcggccgcttttttcacctttgcttttgtgcct |  |
| SfGFP-linker-f | ggtgaaaaaagcggccgcaggaggaggat | Amplification of 2130/H254A for Gibson Assembly with sfGFP  Amplification of sfGFP for Gibson Assembly with 2130/H254A |
| SfGFP-linker-r | ataaatcataagaaattcgcggatcctcatttgtagagctcatccatg |  |
