# Supplementary material for: A broad-spectrum anti-fungal effector dictates bacterial-fungal interkingdom interactions
Source: PLoS Pathog. 2025 Oct 27;21(10):e1013598. doi: 10.1371/journal.ppat.1013598 (PMC12574953; doi:10.1371/journal.ppat.1013598)
Supplement: S4 Table — (DOCX) [file ppat.1013598.s013.docx]

**S4 Table. Primers in this study.**

| **Primer name** | **Sequence (forward/reverse)** | **Description** |
| --- | --- | --- |
| 2127-KO1 | taccgaattcgagctcgagcgtcaggagggagccaatcagag | For deleting the *Aave_2127* gene, located upstream of the *Aave_2127* gene |
| 2127-KO2 | tgtgcttggtcagctgctctcccagggg |  |
| 2127-KO3 | agagcagctgaccaagcacattgggctgg | For deleting the *Aave_2127* gene, located downstream of the *Aave_2127* gene |
| 2127-KO4 | ctgcaggtcgactctgagatctcagtagcggtagtc |  |
| 2127-KO5 | ctgatttgctgcgttgcgag | For confirming the deletion of the *Aave_2127* mutant |
| 2127-KO6 | caccttggggatagtcgatctgc |  |
| 2128-KO1 | acacattatacgagccggaagataacgccgcgaacatgcca | For deleting the *Aave_2128* gene, located upstream of the *Aave_2128* gene |
| 2128-KO2 | tcattggccatggctcgatggttgtgccatagcgtca |  |
| 2128-KO3 | atggcacaaccatcgagccatggccaatgaaaacattgt | For deleting the *Aave_2128* gene, located downstream of the *Aave_2128* gene |
| 2128-KO4 | gcgcacccgtggaaattaattgtccttttccagaccgctaccc |  |
| 2128-KO5 | tcatcgtggacttcctgaacgg | For confirming the deletion of the *Aave_2128* mutant |
| 2128-KO6 | tagttccaaagccgctcctg |  |
| 2129-KO1 | acacattatacgagccggaagaacactgcaactgaccgagc | For deleting the *Aave_2129* gene, located upstream of the *Aave_2129* gene |
| 2129-KO2 | gcttcttccagttttcattggccatggctac |  |
| 2129-KO3 | caatgaaaactggaagaagccgtagcatcc | For deleting the *Aave_2129* gene, located downstream of the *Aave_2129* gene |
| 2129-KO4 | gcgcacccgtggaaattaattgtcgttgtaaggaacctttttgccac |  |
| 2129-KO5 | tgcgatgtgctgctggtgaacg | For deleting the *Aave_2129* gene, located upstream of the *Aave_2129* gene |
| 2129-KO6 | tgcctctttgcatccgtggt |  |
| 2130-KO1 | taccgaattcgagctcgagcgcagatagaggcattcgagatgc | For deleting the *Aave_2130* gene, located upstream of the *Aave_2130* gene |
| 2130-KO2 | ctggattgcttcatctttgacacgcgcgttgc |  |
| 2130-KO3 | cgtgtcaaagatgaagcaatccaggtaggcac | For deleting the *Aave_2130* gene, located downstream of the *Aave_2130* gene |
| 2130-KO4 | ctgcaggtcgactctgagatctcacaaaccatgtttgcgcttg |  |
| 2130-KO5 | acgaagcaggtttaagcaacatgtc | For confirming the deletion of the *Aave_2130* mutant |
| 2130-KO6 | tacgttctcggccgttgatg |  |
| NOLS 1-21aa-KO1 | cacattatacgagccggaagaacggtcagcgtgactatcc | For deleting the NOLS ^1-21aa^ in *Aave_2130* gene, located upstream of the NOLS ^1-21aa^ |
| NOLS 1-21aa-KO2 | aggggtagtccatgggatgctacggcttct |  |
| NOLS 1-21aa-KO3 | tagcatcccatggactacccctcaccgggg | For deleting the NOLS ^1-21aa^ in *Aave_2130* gene, located downstream of the NOLS ^1-21aa^ |
| NOLS 1-21aa-KO4 | gcgcacccgtggaaattaattgtcgttgtaaggaacctttttgccac |  |
| NOLS 1-21aa-KO5 | ctacgttctcggccgttgat | For confirming the deletion of the NOLS ^1-21aa^ mutant |
| NOLS ^1-21aa^-KO6 | gcttttctgtaatcctggcacc |  |
| tssM-KO1 | taccgaattcgagctcgagcggcttcgagggacgctatca | For deleting the *tssM* gene, located upstream of the *tssM* gene |
| tssM-KO2 | gaagcgcagcttcgtgaaccggttgaagaagaatg |  |
| tssM-KO3 | gttcacgaagctgcgcttcgaggtcac | For deleting the *tssM* gene, located downstream of the *tssM* gene |
| tssM-KO4 | ctgcaggtcgactctgagatctctccgcgtcgtaggtgagatc |  |
| tssM-KO5 | catcgagctcatgtacttctgcc | For confirming the deletion of the *tssM* mutant |
| tssM-KO6 | catgaagtggtcgccatcct |  |
| 2130-V5-f | ggtaaacctattcctaatcctctcctt | pBAD24-2130-3V5 plasmid construction for toxicity assay |
| 2130-V5-r | ggtacctcctgctagcccaaa |  |
| 2130 1-159aa-3V5-f | cgaagaggctggtaaacctattcctaatcctctcc | pBAD24-2130 1-159aa- 3V5 plasmid construction for toxicity assay |
| 2130 1-159aa-3V5-r | taggtttaccagcctcttcggcttccttgg |  |
| 2130 160-333aa-3V5-f | catggctgccgcggccaagaaggcggaaggcaat | pBAD24-2130 160-333aa-3V5 plasmid construction for toxicity assay |
| 2130 160-333aa-3V5-r | tcttggccgcggcagccatgccagcgccagggatcat |  |
| 2130 200-333aa-3V5-f | ttgggctagcaggaggtaccatggaaaaggaccacacgcc | pBAD24-2130 200-333aa-3V5 plasmid construction for toxicity assay |
| 2130 200-333aa-3V5-r | ggtacctcctgctagcccaaa |  |
| 2130 250-333aa-3V5-f | ttgggctagcaggaggtaccatgccgcctgacatacatgc | pBAD24-2130 250-333aa-3V5 plasmid construction for toxicity assay |
| 2130 250-333aa-3V5-r | ggtacctcctgctagcccaaa |  |
| 2130 P81A-3V5-f | ggctgatcGcgggtgtcggcgagatcgcggatggcgcaaa | pBAD24-2130P81A-3V5 plasmid construction for toxicity assay |
| 2130 P81A-3V5-r | ctcgccgacacccgCgatcagcccgacgacatccagcccg |  |
| 2130 N91A-3V5-f | atcgcggatggcgcaGCCgcgctgatctatctg | pBAD24-2130N91A-3V5 plasmid construction for toxicity assay |
| 2130 N91A-3V5-r | cagatagatcagcgcgGCtgcgccatccgcgatctcgccgaca |  |
| 2130 Y95A3V5-f | atcgcggatggcgcaGCcgcgctgatctatctggcggagggcga | pBAD24-2130Y95A-3V5 plasmid construction for toxicity assay |
| 2130 Y95A-3V5-r | tcgccctccgccagaGCgatcagcgcgtttgcgccatccgcgat |  |
| 2130 G99AD100A-3V5-f | ctggcggaggCcgCtaaggtcaacgccgccatcagcgct | pBAD24-2130G99AD100A-3V5 plasmid construction for toxicity assay |
| 2130 G99AD100A-3V5-r | gttgaccttaGcgGcctccgccagatagatcagcgcgtt |  |
| 2130 R151A-3V5-f | cgctggtgaaaGCggaggccaaggaagccgaagaggctg | pBAD24-2130R151A-3V5 plasmid construction for toxicity assay |
| 2130 R151A-3V5-r | ttggcctccGCtttcaccagcgcctcttccgcctc |  |
| 2130 P250A-3V5-f | cctacaatagctattGcgcctgacatacatgct | pBAD24-2130P250A-3V5 plasmid construction for toxicity assay |
| 2130 P250A-3V5-r | tatgtcaggcgCaatagctattgtaggcgcattatttctaacgg |  |
| 2130 H254A-3V5-f | attccgcctgacataGCtgctctaggcgatacttggcg | pBAD24-2130H254A-3V5 plasmid construction for toxicity assay |
| 2130 H254A-3V5-r | agtatcgcctagagcaGCtatgtcaggcggaatagctattgta |  |
| 2130-2131-3V5-f | ttgggctagcaggaggtaccatggcgacgaagagacct | pBAD24-2130-2131-3V5 plasmid construction for toxicity assay |
| 2130-2131-3V5-r | ggattaggaataggtttacctggacaaatattattctttcgaaacag |  |
| 2130-2132-3V5-f | atgaatgcaatagctctgattgattttttaggacac | pBAD24-2130-2132-3V5 plasmid construction for toxicity assay |
| 2130-2132-3V5-r | atcagagctattgcattcattttttcacctttgctt |  |
| pCH363-2131-f | cctgcaggtcgactctagagatgaatgcaattgatttgat | pCH363-2131 plasmid construction for Bacterial two-hybrid assay |
| pCH363-2131-r | gaattcgagctcggtaccgctggacaaatattattctttc |  |
| pCH363-2130H254A-f | cctgcaggtcgactctagagatggcgacgaagagacct | pCH363-2130H254A plasmid construction for Bacterial two-hybrid assay |
| pCH363-2130H254A-r | gaattcgagctcggtaccgcttttttcacctttgcttttgtgcc |  |
| pKNT25N-2131-f | caaagcttgcatgctctagaatgaatgcaattgatttgat | pKNT25N-2131 plasmid construction for Bacterial two-hybrid assay |
| pKNT25N -2131-r | atcgatgggaattcggtacctcatggacaaatattattc |  |
| pKNT25N -2130H254A-f | caaagcttgcatgctctagaatggcgacgaagagacct | pKNT25N-2130H254A plasmid construction for Bacterial two-hybrid assay |
| pKNT25N -2130H254A-r | atcgatgggaattcggtacctcattttttcacctttgctt |  |
| His-SUMO-2130/H254A-f | tggtggtggatccggtaccatggcgacgaagagacctc | pETDuet-His-SUMO-2130/H254A-2131  plasmid construction for protein purification |
| His-SUMO-2130/H254A-r | ttaagcattatgcggccgctcattttttcacctttgct |  |
| His-SUMO-2131-f | cgatcgctgacgtcggtaccatgaatgcaattgatttgat |  |
| His-SUMO-2131-r | ggtttaccaccagactcgagtcatggacaaatattattct |  |
| His-SUMO-2130_H254A_^CT^-f | gtggtggatccggtaccatggcggccaagaaggcggaagg | pETDuet-His-SUMO-2130_H254A_^CT^ -2131 plasmid construction for protein purification |
| His-SUMO-2130_H254A_^CT^-r | ttaagcattatgcggccgctcattttttcacctttgct |  |
| pBBR1MCS2-Flag-2128-f | acaaggacgacgatgacaagatggcacaaccatcgcacg | pBBR1MCS2-Flag-2128 plasmid construction for pull-down assay |
| pBBR1MCS2-Flag-2128-r | cttgtcatcgtcgtccttgtaatccatggtttcctcctgtgtgaaattgt |  |
| pET28a-flag-2129-f | atggattacaaggacgacgatgacaagatggccaatgaaaacattgtc | pET28a-flag-2129 plasmid construction for pull-down assay |
| pET28a -flag-2129-r | cagcttcctttcgggctttgctacggcttcttccagttcatc |  |
| pBAD24-Flag-2127N-term-f | gggctagcaggaggtaccatggattacaaggacgacgatgac | pBAD24-Flag-2127N-term plasmid construction for pull-down assay |
| pBAD24-Flag-2127N-term-r | aacagccaagcttttattaccacagctgctccgcgc |  |
| pBAD24-Flag-2127Core-f | acaaggacgacgatgacaagatgctgcatgcgcagaagg | pBBR1MCS2-Flag-2128 plasmid construction for pull-down assay |
| pBAD24-Flag-2127Core-r | aaacagccaagcttttattaactgccgaccttgatcgacc |  |
| pBAD24-Flag-2127C-term-f | acaaggacgacgatgacaagatgttcaagaccgagaccat | pBAD24-Flag-2127Core plasmid construction for pull-down assay |
| pBAD24-Flag-2127C-term-r | aaacagccaagcttttattaattcaggtcgatgcgcttgg |  |
| pBAD24-Flag-2127-f | gggctagcaggaggtaccatggattacaaggacgacgatg | pBAD24-Flag-2127C plasmid construction for pull-down assay |
| pBAD24-Flag-2127-r | aacagccaagcttttattaattcaggtcgatgcgcttgga |  |
| 2130/H254A-linker-f | tcaaggagtctagaaagcttggatccatggcgacgaagagacct | Amplification of 2130/H254A for Gibson Assembly with sfGFP  Amplification of 2130/H254A for Gibson Assembly with sfGFP |
| 2130/H254A-linker-r | ctgcggccgcttttttcacctttgcttttgtgcct |  |
| SfGFP-linker-f | ggtgaaaaaagcggccgcaggaggaggat | Amplification of 2130/H254A for Gibson Assembly with sfGFP  Amplification of sfGFP for Gibson Assembly with 2130/H254A |
| SfGFP-linker-r | ataaatcataagaaattcgcggatcctcatttgtagagctcatccatg |  |
