# Supplementary material for: A broad-spectrum anti-fungal effector dictates bacterial-fungal interkingdom interactions
Source: PLoS Pathog. 2025 Oct 27;21(10):e1013598. doi: 10.1371/journal.ppat.1013598 (PMC12574953; doi:10.1371/journal.ppat.1013598)
Supplement: S1 Data — (DOCX) [file ppat.1013598.s014.docx]

**1. Alignment sequence (1-59aa)**

>Aave_2130.1:1-59 hypothetical protein [Paracidovorax citrulli]

MATKRPRPPEAQTRNARVKPNDYPSPGPIGNTTGIVVPDTAPNPHGNSAPPIKPTEPEK

>WP_208935568.1:1-59 hypothetical protein [Paracidovorax avenae]

MATKRPRPPEAQTRNARVKPNDYPSPGPIGNTTGIVVPDTAPNPHGNSAPPIKPTEPEK

>WP_081483853.1:1-59 MULTISPECIES: hypothetical protein [Paracidovorax]

MATKRPRPPEAQTRNARVKPNDYPSPGPIGNTTGIVVPDTAPNPHGNSAPPIKPTEPEK

>WP_208942746.1:1-59 hypothetical protein [Paracidovorax avenae]

MATKRPRPPEAQTRNARVKPNDYPSPGPIGNTTGIVVPDTAPNPHGNSAPPIKPTEPEK

>WP_284408693.1:1-59 hypothetical protein [Acidovorax sp. SUPP2539]

MASKRPRPPEAETRNARVKPNNYPSPGPIGNTTGIVVPDTAPNPHGNSAPPIKPTEPEK

>WP_228316160.1:1-59 hypothetical protein [Xanthomonas sp. NCPPB 1067]

MASKRPRPPEAETRNARVKPNNYPSPGPIGNTTGIVVPDTAPNPHGNSAPPIKPTEPEK

>WP_274340481.1:1-59 hypothetical protein [Xanthomonas campestris]

MASKRPRPPEAETRNARVKPNNYPSPGPIGNTTGIVVPDTAPNPHGNSAPPIKPTEPEK

>WP_092740276.1:1-59 MULTISPECIES: hypothetical protein [Pseudomonadota]

MASKRPRPPEAETRNARVKPNNYPSPGPIGNTTGIVVPDTAPNPHGNSAPPIKPTEPEK

>WP_245711377.1:1-59 hypothetical protein [Paracidovorax valerianellae]

MASKRPRPPQADTRNARVKPNDYPSPGPIGNTTGIVVPDTAPNPHGNSAPPIKPTEPEK

>WP_284429989.1:1-59 hypothetical protein [Acidovorax sp. SUPP950]

MAPKRPRPPEAETRNARIKPNDYPAPGPIGNTTGIVVPDTAPNPHGNSAPPIKPTETEK

>WP_233174933.1:1-59 hypothetical protein [Delftia sp. ASV31]

MATRKKQLPAAQSQTAQVKPNAYPSPGPIGNTSGLVIPDTAPNLHGATAPAIKPVAQEK

>WP_252980587.1:1-59 hypothetical protein [Delftia acidovorans]

MATRKKQLPAAQSQTAQVKPNAYPSPGPIGNTSGLVIPDTAPNLHGATAPAIKPVAQEK

>WP_279213085.1:1-59 hypothetical protein [Delftia acidovorans]

MATRKKQLPAAQSQTAQVKPNAYPSPGPIGNTSGLVIPDTAPNLHGATAPAIKPVAQEK

>WP_224986429.1:1-59 hypothetical protein [Delftia acidovorans]

MATRKKQLPAAQSQTAQVKPNAYPSPGPIGNTSGLVIPDTAPNLHGATAPAIKPVAQEK

>MCP4532358.1:1-59 hypothetical protein [Delftia sp.]

MATRKKQLPAAQSQTAQVKPNAYPSPGPIGNTSGLVIPDTAPNLHGATAPAIKPVAQEK

>WP_237736819.1:1-59 hypothetical protein [Delftia acidovorans]

MATRKKQLPAAQSQTAQVKPNAYPSPGPIGNTSGLVIPDTAPNLHGATAPAIKPVAQEK

>WP_082848130.1:1-59 MULTISPECIES: hypothetical protein [Delftia]

MATRKKQLPAAQSQTAQVKPNAYPSPGPIGNTSGLVIPDTAPNLHGATAPAIKPVAQEK

>WP_310635572.1:1-59 hypothetical protein [Delftia acidovorans]

MATRKKQLPAAQSQTAQVKPNAYPSPGPIGNTSGLVIPDTAPNLHGATAPAIKPVAQEK

>WP_197943477.1:1-59 hypothetical protein [Delftia acidovorans]

MATRKKQLPAAQSQTAQVKPNAYPSPGPIGNTSGLVIPDTAPNLHGATAPAIKPVAQEK

>WP_012205696.1:1-59 MULTISPECIES: hypothetical protein [Delftia]

MATRKKQLPAAQSQTAQVKPNAYPSPGPIGNTSGLVIPDTAPNLHGATAPAIKPVAQEK

>WP_279495373.1:1-59 hypothetical protein [Delftia tsuruhatensis]

MATRKKQLPAAQSQTAQVKPNAYPSPGPIGNTSGLVIPDTAPNLHGATAPAIKPVAQEK

>WP_016447005.1:1-59 MULTISPECIES: hypothetical protein [Delftia]

MATRKKQLPAAQSQTAQVKPNAYPSPGPIGNTSGLVIPDTAPNLHGATAPAIKPVAQEK

>WP_013802343.1:1-59 hypothetical protein [Delftia sp. Cs1-4]

MATRKKQLPAAQSQTAQVKPNAYPSPGPIGNTSGLVIPDTAPNLHGATAPAIKPVAQEK

>WP_349335710.1:1-59 hypothetical protein [Delftia sp. DS1230]

MATRKKQLPAAQSQTAQVKPNAYPSPGPIGNTSGLVIPDTAPNLHGATAPAIKPVAQEK

>WP_279213078.1:1-59 hypothetical protein [Delftia acidovorans]

MATRKKQLPAAQSQTAQVKPNAYPSPGPIGNTSGLVIPDTAPNLHGATAPAIKPVAQEK

>WP_272548440.1:1-64 hypothetical protein [Acidovorax sp. NCPPB 3576]

MATRKRGPDDDPSDAAQTPAARIKPNNWSSPGPIGNTTGLVVPETAPNPHFDSAPPIKPTEPEK

>WP_237736816.1:1-59 hypothetical protein [Delftia acidovorans]

MATRKKQLPAAQSQTAQVKPNAYPSPGPIGNTSGLVIPDTAPNLHGATAPAIKPVAQEK

>WP_269198945.1:1-59 hypothetical protein [Delftia acidovorans]

MATRKKQLPAAQSQTAQVKPNAYPSPGPIGNTSGLVIPDTAPNLHGATAPAIKPVAQEK

>WP_175525996.1:1-64 hypothetical protein [Paracidovorax konjaci]

MATRKRGPDDDPSDAAQPRAARIKPNSWSSPGPIGNTTGLVVPETAPNPHFASAPAIKPTETEK

>WP_157659189.1:8-59 polymorphic toxin type 15 domain-containing protein [Thauera butanivorans]

MSGKRPTVSAQVKPNAQPTPGPMGNTQGIIVPDTGSNPHGNSAPPIQATAKE

>WP_199140333.1:1-36 hypothetical protein, partial [Delftia sp. ASV31]

PSPGPIGNTSGLVIPDTAPNLHGATAPAIKPVAQEK

>WP_341645986.1:1-30 hypothetical protein [Thauera sp. SDU_THAU2]

MGNTQGIIVPDTGPNPHGNSAPPIQATAKE

>WP_341645981.1:1-30 hypothetical protein [Thauera sp. SDU_THAU2]

MGNTQGIIVPDTGPNPHGNSAPPIQATAKE

>WP_325281368.1:35-69 hypothetical protein [Tahibacter sp.]

GRVKPNSQRKPGPIGNTQGIIVPQTSPNPGGNSAP

>MCB1915650.1:6-49 hypothetical protein [Rhodocyclaceae bacterium]

VKPNHYAKPGPQGNTTGIVTPQTRPNASGPDNSAPPPKPAEEEK

>WP_092383255.1:87-121 hypothetical protein [Halopseudomonas salegens]

QAQQGRIQPNNYPQPGPIGNTTGIIVPKTSAAPAG

>MBK6975199.1:11-57 hypothetical protein [Candidatus Methylophosphatis roskildensis]

RLGRVKPNNRPSPGPLGNTQGPILPETSPNASGAGNSAPPPRPPEAE

>MBK7237259.1:11-57 hypothetical protein [Sterolibacteriaceae bacterium]

RLGRVKPNNRPSPGPLGNTQGPILPETSPNASGAGNSAPPPRPPEAE

>WP_225981646.1:1-31 hypothetical protein [Paracidovorax avenae]

MGNTTGLVVPATSPNPHANSAPAIQPTPQEK

>MCX7113283.1:2-41 hypothetical protein [Pseudomonadota bacterium]

PEELNKGAVKPNAQASPGPIGNTQGIIVPKTATHPDGDSA

**2. Alignment sequence (60-120aa)**

>Aave_2130.1:60-120 hypothetical protein [Paracidovorax citrulli]

SWWSRWGSDVVHTGLDVVGLIPGVGEIADGANALIYLAEGDKVNAAISAAAMIPGAGMAAT

>WP_081483853.1:60-120 MULTISPECIES: hypothetical protein [Paracidovorax]

SWWSRWGSDVVHTGLDVVGLIPGVGEIADGANALIYLAEGDKVNAAISAAAMIPGAGMAAT

>WP_082091343.1:60-120 hypothetical protein [Paracidovorax citrulli]

SWWSRWGSDVVHTGLDVVGLIPGVGEVADGANALIYLAEGDKVNAAISAAAMIPGAGMAAT

>WP_208935568.1:60-120 hypothetical protein [Paracidovorax avenae]

SWWSRWGSDVVHTGLDVVGLIPGVGEVADGANALIYLAEGDKVNAAISAAAMIPGAGMAAT

>WP_208942746.1:60-120 hypothetical protein [Paracidovorax avenae]

SWWSRWGSDVVHTGLDIVGLIPGVGEVADGANALIYLAEGDKVNAAISAAAMIPGAGMAAT

>WP_012205696.1:60-120 MULTISPECIES: hypothetical protein [Delftia]

SWWQRWGSDVVHTGLDVVGLIPGVGEIADGANALIYLAEGDKVNAALSAAAMIPGAGMAAT

>WP_197943477.1:60-120 hypothetical protein [Delftia acidovorans]

SWWQRWGSDVVHTGLDVVGLIPGVGEIADGANALIYLAEGDKVNAALSAAAMIPGAGMAAT

>WP_310635572.1:60-120 hypothetical protein [Delftia acidovorans]

SWWQRWGSDVVHTGLDVVGLIPGVGEIADGANALIYLAEGDKVNAALSAAAMIPGAGMAAT

>WP_082848130.1:60-120 MULTISPECIES: hypothetical protein [Delftia]

SWWQRWGSDVVHTGLDVVGLIPGVGEIADGANALIYLAEGDKVNAALSAAAMIPGAGMAAT

>WP_284429989.1:60-120 hypothetical protein [Acidovorax sp. SUPP950]

SWWSRWGSDVVHTGLDVIGLIPGAGEIADGANALIYLAEGDKVNAAISAAAMIPGAGMAAT

>WP_013802343.1:60-120 hypothetical protein [Delftia sp. Cs1-4]

SWWQRWGSDVVHTGLDVVGLIPGVGEIADGANALIYLAEGDKVNAALSAAAMIPGAGMAAT

>WP_016447005.1:60-120 MULTISPECIES: hypothetical protein [Delftia]

SWWQRWGSDVVHTGLDVVGLIPGVGEIADGANALIYLAEGDKVNAALSAAAMIPGAGMAAT

>WP_279495373.1:60-120 hypothetical protein [Delftia tsuruhatensis]

SWWQRWGSDVVHTGLDVVGLIPGVGEIADGANALIYLAEGDKVNAALSAAAMIPGAGMAAT

>WP_349335710.1:60-120 hypothetical protein [Delftia sp. DS1230]

SWWQRWGSDVVHTGLDVVGLIPGVGEIADGANALIYLAEGDKVNAALSAAAMIPGAGMAAT

>WP_237736819.1:60-120 hypothetical protein [Delftia acidovorans]

SWWQRWGSDVVHTGLDVVGLIPGVGEIADGANALIYLAEGDKVNAALSAAAMIPGAGMAAT

>MCP4532358.1:60-120 hypothetical protein [Delftia sp.]

SWWQRWGSDVVHTGLDVVGLIPGVGEIADGANALIYLAEGDKVNAALSAAAMIPGAGMAAT

>WP_274340481.1:60-120 hypothetical protein [Xanthomonas campestris]

SWWSRWGSDVVHTGLDVVGLIPGAGEIADGANALIYLAEGDKVNAAISAAAMIPGAGMAST

>WP_228316160.1:60-120 hypothetical protein [Xanthomonas sp. NCPPB 1067]

SWWSRWGSDVVHTGLDVVGLIPGAGEIADGANALIYLAEGDKVNAAISAAAMIPGAGMAST

>WP_284408693.1:60-120 hypothetical protein [Acidovorax sp. SUPP2539]

SWWSRWGSDVVHTGLDVVGLIPGAGEIADGANALIYLAEGDKVNAAISAAAMIPGAGMAST

>WP_245711377.1:60-120 hypothetical protein [Paracidovorax valerianellae]

SWWSRWGSDVVHTGLDVVGLIPGAGEIADGANALIYLAEGDKVNAAISAAAMIPGAGMAAT

>WP_092740276.1:60-120 MULTISPECIES: hypothetical protein [Pseudomonadota]

SWWSRWGSDVVHTGLDVVGLIPGAGEIADGANALIYLAEGDKVNAAISAAAMIPGAGMAST

>WP_269198945.1:60-120 hypothetical protein [Delftia acidovorans]

SWWQRWGSDVVHTGLDVVGLIPGVGEIADGANALIYLAEGDKVNAALSAAAMIPGAGMAAT

>WP_237736816.1:60-120 hypothetical protein [Delftia acidovorans]

SWWQRWGSDVVHTGLDVVGLIPGVGEIADGANALIYLAEGDKVNAALSAAAMIPGAGMAAT

>WP_199140333.1:37-97 hypothetical protein, partial [Delftia sp. ASV31]

SWWQRWGSDVVHTGLDVVGLIPGVGEIADGANALIYLAEGDKVNAALSAAAMIPGAGMAAT

>WP_279213078.1:60-120 hypothetical protein [Delftia acidovorans]

SWWQRWDRDVVHTGLDVVGLIPGVGEIADGANALIYLAEGDKVNAALSAAAMIPGAGMAAT

>WP_279213085.1:60-120 hypothetical protein [Delftia acidovorans]

SWWQRWDRDVVHTGLDVVGLIPGVGEIADGANALIYLAEGDKVNAALSAAAMIPGAGMAAT

>WP_224986429.1:60-120 hypothetical protein [Delftia acidovorans]

SWWQHWGSDVVHTGLDVMGLVPGVGEIADGPNALIYLAEGDKVNAALSAAAMIPGAGMAAT

>WP_175525996.1:66-125 hypothetical protein [Paracidovorax konjaci]

WWASWGSDVLHTGLDVVGLVPGFGEVADGANALIYLAEGDKVNAAISAAAMIPGLGAGAT

>WP_252980587.1:60-112 hypothetical protein [Delftia acidovorans]

SWWQRWGSDVVHTGLDVVGLIPGVGEIADGANALIYLAEGDKVNAALSAAAMI

>WP_298624525.1:52-111 polymorphic toxin type 15 domain-containing protein [uncultured Zoogloea sp.]

WWNRWGSDALHTTLDVVGLIPGVGEVADGANALIYLAEGDKVSAALSAAAMVPFGGMAAT

>WP_225981646.1:32-92 hypothetical protein [Paracidovorax avenae]

SWWESWGSDVVHVGLDAVGLIPGLGEVADGANALIYLAEGDTTNAAISAAAMIPGLGAGAT

>WP_231502176.1:24-84 hypothetical protein [Paracidovorax avenae]

NWWESWGSDVVHVGLDAVGLIPGLGEVADGANALIYLAEGDTTNAAISAAAMIPGLGAGAT

>WP_341645986.1:33-92 hypothetical protein [Thauera sp. SDU_THAU2]

WWGRWGSAVAHGVLDVVGLIPVVGEVADGANALIYLAEGDTVNAALSAAAMIPGAGMAAT

>WP_272548440.1:66-125 hypothetical protein [Acidovorax sp. NCPPB 3576]

WWTSWGSNVLHTGLDAVGLVPGFGEVADGTNALIYLAEGDKVNAAISAVAMIPGLGAGAT

>WP_341645981.1:33-92 hypothetical protein [Thauera sp. SDU_THAU2]

WWGRWGSAVAHGVLDVVGLIPVVGEVADGANALIYLAEGDTVNAALSAAAMIPGAGMAAT

>MCB1915650.1:51-110 hypothetical protein [Rhodocyclaceae bacterium]

WWGSWGSDLLHTGLDVVGLIPVVGEVADGANALIYLAEGDKVNAALSAASMLPVGGQAAT

>WP_157659189.1:62-121 polymorphic toxin type 15 domain-containing protein [Thauera butanivorans]

WWGRWGSAVTHGVLDVVGLIPVVGEIADGANALIYLAEGDTVNAALSAAAMVPGLGMAAT

>WP_233626401.1:1-51 MULTISPECIES: hypothetical protein [unclassified Delftia]

MHTGLDVVGLIPGVGEIADGANALIYLAEGDKVNAALSAAAMIPAAGMAAT

>MBK7663616.1:60-119 hypothetical protein [Sterolibacteriaceae bacterium]

WWQRWGSDAVRTGLDIVGLIPVVGEVADGANAMIYLAEGDKVNAASIAASMLPLGGQAAK

>MBK6975199.1:60-119 hypothetical protein [Candidatus Methylophosphatis roskildensis]

WWQRWGSDAVRTGLDIVGLIPVVGEVADGANAMIYLAEGDKVNAASIAASMLPLGGQAAK

**3. Alignment sequence (99-159aa)**

>Aave_2130.1:99-159 hypothetical protein [Paracidovorax citrulli]

GDKVNAAISAAAMIPGAGMAATGAKYGKKAAGAAAEAIGKKTAREAEEALVKREAKEAEEA

>WP_208942746.1:99-159 hypothetical protein [Paracidovorax avenae]

GDKVNAAISAAAMIPGAGMAATGAKYGKKVAGAAAEAIGKKTAREAEEALVKREVKEAEEA

>WP_082091343.1:99-159 hypothetical protein [Paracidovorax citrulli]

GDKVNAAISAAAMIPGAGMAATGAKYGKKAAGAAAEAIGKKTAREAEEALAKREAKEAEEA

>WP_208935568.1:99-159 hypothetical protein [Paracidovorax avenae]

GDKVNAAISAAAMIPGAGMAATGAKYGKKVAGAAAEAIGKKTAREAEEALVKREVKEAEEA

>WP_081483853.1:99-159 MULTISPECIES: hypothetical protein [Paracidovorax]

GDKVNAAISAAAMIPGAGMAATGAKYGKKAAGAAAEAIGKKTAREAEEALAKREAKEAEEA

>WP_245711377.1:99-159 hypothetical protein [Paracidovorax valerianellae]

GDKVNAAISAAAMIPGAGMAATGAKYGKKAVGAAAEAVGKKTAREAEEALAKREAKEAEEA

>WP_284429989.1:99-159 hypothetical protein [Acidovorax sp. SUPP950]

GDKVNAAISAAAMIPGAGMAATGAKYGKKAVGAAAEAVGKKTAREAEEALAKREAKEAEEA

>WP_284408693.1:99-148 hypothetical protein [Acidovorax sp. SUPP2539]

GDKVNAAISAAAMIPGAGMASTGAKYGKKAADAAVEAVGKKTAREAEEAL

>WP_274340481.1:99-148 hypothetical protein [Xanthomonas campestris]

GDKVNAAISAAAMIPGAGMASTGAKYGKKAADAAVEAVGKKTAREAEEAL

>WP_228316160.1:99-148 hypothetical protein [Xanthomonas sp. NCPPB 1067]

GDKVNAAISAAAMIPGAGMASTGAKYGKKAADAAVEAVGKKTAREAEEAL

>WP_224986429.1:99-147 hypothetical protein [Delftia acidovorans]

GDKVNAALSAAAMIPGAGMAATGSKYGKKAAAAATEAVGKKSGREAAEA

>WP_157659189.1:100-151 polymorphic toxin type 15 domain-containing protein [Thauera butanivorans]

GDTVNAALSAAAMVPGLGMAATGAKVGKRAVGAAVEGTGKAVGKETAQQASQ

>WP_231502176.1:63-115 hypothetical protein [Paracidovorax avenae]

GDTTNAAISAAAMIPGLGAGATAAKYGKKVATAAGEAVGKAAGRETAEAAAKQ

>WP_225981646.1:71-123 hypothetical protein [Paracidovorax avenae]

GDTTNAAISAAAMIPGLGAGATAAKYGKKVATAAGEAVGKAAGRETAEAAAKQ

>WP_341645981.1:71-126 hypothetical protein [Thauera sp. SDU_THAU2]

GDTVNAALSAAAMIPGAGMAATGAKYAKKAGTAVEGAIKGGRETAEHAGKKEAGEA

>WP_341645986.1:71-126 hypothetical protein [Thauera sp. SDU_THAU2]

GDTVNAALSAAAMIPGAGMAATGAKYAKKAGTAVEGAIKGGRETAEHAGKKEAGEA

>WP_175525996.1:104-143 hypothetical protein [Paracidovorax konjaci]

GDKVNAAISAAAMIPGLGAGATATKYGKKAAEMGAEALGK

>WP_272548440.1:104-143 hypothetical protein [Acidovorax sp. NCPPB 3576]

GDKVNAAISAVAMIPGLGAGATATKYGKQVAEAGAEALGK

>WP_254662146.1:7-66 hypothetical protein [Delftia sp. JD2]

GDKVNAALSAAAMIPGTGMAATGAKYGKKAAAAATEAVGKKAGREAAEAGLQKSAKEAEE

>MBN1853268.1:36-95 hypothetical protein [Pirellulales bacterium]

GDTLNAGISLAGCIPGAGQAATGARLGAKVLKEGAEQVIKKGTKEVAEQAIKKGTKEVTE

>WP_233626401.1:30-56 MULTISPECIES: hypothetical protein [unclassified Delftia]

GDKVNAALSAAAMIPAAGMAATGVKYG

>WP_020464764.1:194-250 PAAR-like domain-containing protein [Sorangium cellulosum]

GDYLNAAISAAGMIPFGGAAATGGRLAAKAGKAVAEAVGKEGAQEAAQQVGKQGAEQ

>KYG08217.1:194-250 hypothetical protein BE21_24965 [Sorangium cellulosum]

GDYLNAAISAAGMIPFGGAAATGGRLAAKAGKAVAEAVGKEGAQEAAQQVGKQGAEQ

>KYF50367.1:194-250 hypothetical protein BE04_33405 [Sorangium cellulosum]

GDYLNAAISAAGMIPFGGAAATGGRLAAKAGKAVAEAVGKEGAQEAAQQVGKQGAEQ

>WP_013802343.1:99-155 hypothetical protein [Delftia sp. Cs1-4]

GDKVNAALSAAAMIPGAGMAATGAKYGKKAAAAATEAVGKKIGREASEEAAGKAAKE

>WP_325278201.1:163-198 hypothetical protein [Tahibacter sp.]

GDYVNAALDAAAMWPAGGQAATAAKYGKKGVGAAIE

>WP_237736819.1:99-147 hypothetical protein [Delftia acidovorans]

GDKVNAALSAAAMIPGAGMAATGAKYGKKAAAAATEAVGKKAGREAAEA

>WP_310635572.1:99-147 hypothetical protein [Delftia acidovorans]

GDKVNAALSAAAMIPGAGMAATGAKYGKKAAAAATEAVGKKAGREAAEA

>WP_012205696.1:99-147 MULTISPECIES: hypothetical protein [Delftia]

GDKVNAALSAAAMIPGAGMAATGAKYGKKAAAAATEAVGKKAGREAAEA

>MCP4532358.1:99-147 hypothetical protein [Delftia sp.]

GDKVNAALSAAAMIPGAGMAATGAKYGKKAAAAATEAVGKKAGREAAEA

>WP_279213085.1:99-147 hypothetical protein [Delftia acidovorans]

GDKVNAALSAAAMIPGAGMAATGAKYGKKAAAAATEAVGKKAGREAAEA

>WP_269198945.1:99-147 hypothetical protein [Delftia acidovorans]

GDKVNAALSAAAMIPGAGMAATGAKYGKKAAAAATEAVGKKAGREAAEA

>WP_267168658.1:2459-2510 SpvB/TcaC N-terminal domain-containing protein [Sulfitobacter sp. F26169L]

GDYVDAGLSFAAMIPFAGWFATAGKFGKKAVNAADLAGDVGRNVTKYGDEAL

>WP_325281365.1:163-221 hypothetical protein [Tahibacter sp.]

GDYVSAAMDLAAMIPTGGQAATAAKYGRKVGNEVVEQAAKKTDDAAEVVAKQSDEAAEA

>WP_232245786.1:92-121 hypothetical protein [Delftia acidovorans]

GDKVNAALSEAAMVPGAGMAVTGVKLQKTS

>MCB9126839.1:151-210 WXG100 family type VII secretion target [Ardenticatenales bacterium]

GDYLNAGLSAAAMIPFAGWGATGAKFGIRAGRELLEEGAERVVREGVEEGAERLGREGAE

>NOQ26942.1:1652-1681 PKD domain-containing protein [Bacteroidales bacterium]

GDKINAGLSTAAMIPLAGMSATVLKYGNKT

>MCB0077369.1:151-210 WXG100 family type VII secretion target [Anaerolineales bacterium]

GDYLNAGLSAAAMIPFAGWGATGAKFGIRAGRELLEEGAERVVREGVEEGAERLGREGAE

**4. Alignment sequence (160-332aa)**

>Aava_2130.1:160-332 hypothetical protein [Paracidovorax citrulli]

AAKKAEGNGGGKDKAKPKKHKDCGKKVPYNDKKSLKGSGLEKDHTPSGAALELAAQNKIDELIANGARITEKQQKAILNS

VRNNAPTIAIPPDIHALGDTWRAKNKPHVIEKDAANLKDAVARNTKTISEAMKNKDHGCKEAYDKAAEELRNMDWEKYID

EAIQVGTKAKVKK

>WP_208942746.1:160-332 hypothetical protein [Paracidovorax avenae]

AAKKAEGNGGGKDKAKPKKHKDCGKKVPYNDKKSLKGSGLEKDHTPSGAALELAAQNKIDELIANGARITEKQQKAILNS

VRNNAPTIAIPPDIHALGDTWRAKNKPHVIEKDAANLKDAVARNTKTISEAMRNKDHGCKEAYDKAAEELRNMDWDKYID

DAIQVGTKAKVKK

>WP_208935568.1:160-332 hypothetical protein [Paracidovorax avenae]

AAKKAEGNGGGKDKAKPKKHKDCGKKVPYNDKKSLKGSGLEKDHTPSGAALELAAQNKIDELIANGARITEKQQKAILNS

VRNNAPTIAIPPDIHALGDTWRAKNKPHVIEKDAANLKDAVARNTKKISEAMRNKDHGCKEAYDKAAEELRNMDWDKYID

DAIQAGTKAKVKK

>WP_274340481.1:166-332 hypothetical protein [Xanthomonas campestris]

GNGGGKDTGKPKRHKDCGKKVPYNDRKSLKGSGLEKDHTPSGAALELAAQNKIDELVASGARITEDQQKAIVNSVRNNAP

TIAVPPDIHAQGDTWKHKNTPANIERDAGNLNDAVKRNTNAISKAMEGKDHGCKEAYDKAAEELRNMDWNKFIDGAIEAG

TKSKVKK

>WP_284408693.1:166-329 hypothetical protein [Acidovorax sp. SUPP2539]

GNAGGKDTGKPKRHKDCGKKVPYNDRKSLKGSGLEKDHTPSGAALEHAAELKILELRESGMRINEIQAKKILNAVRNQAP

TIAIPPDIHSDGDTWRHKNTPEKIKKDASDLKGAVKRNTEKISKAMEGKDHGCKDAYDKAAEELRNMDWDKYIQDAIDSA

IKAK

>WP_228316160.1:166-329 hypothetical protein [Xanthomonas sp. NCPPB 1067]

GNAGGKDTGKPKRHKDCGKKVPYNDRKSLKGSGLEKDHTPSGAALEHAAELKILELRESGMRINEIQAKKILNAVRNQAP

TIAIPPDIHSDGDTWRHKNTPEKIKKDASDLKGAVKRNTEKISKAMEGKDHGCKDAYDKAAEELRNMDWDKYIQDAIDNA

IKAK

>WP_013802343.1:173-326 hypothetical protein [Delftia sp. Cs1-4]

PKRHKDCGKKVPYNDKKSLKGSGLEKDHTPSGAALEKAAENKINELRDSGVKISDAQASEIRKSVRNNAPTIAVPPDIHA

EGQTWRYKNTPERISQDAGNLNEAVKRNTDAISKAMENKDHGCKDAYDKAAQELRNMDWDQYIQDAITNGTKAK

>WP_016447005.1:173-326 MULTISPECIES: hypothetical protein [Delftia]

PKRHKDCGKKVPYNDKKSLKGSGLEKDHTPSGAALEKAAENKINELRDSGVKISDAQASEIRKSVRNNAPTIAVPPDIHA

EGQTWRYKNTPERISQDAGNLNEAVKRNTDAISKAMENKDHGCKDAYDKAAQELRNMDWDQYIQDAITNGTKAK

>WP_279213078.1:173-326 hypothetical protein [Delftia acidovorans]

PKRHKDCGKKVPYNDKKSLKGSGLEKDHTPSGAALEKAAENKINELRDSGVKISDAQASEIRKSVRNNAPTIAVPPDIHA

EGQTWRYKNTPERISQDAGNLNEAVKRNTDAISKAMENKDHGCKDAYDKAAQELRNMDWDQYIQDAITNGTKAK

>WP_199140333.1:150-303 hypothetical protein, partial [Delftia sp. ASV31]

PKRHKDCGKKVPYNDKKSLKGSGLEKDHTPSGAALEKAAENKINELRDSGVKISDAQASEIRKSVRNNAPTIAVPPDIHA

EGQTWRYKNTPERISQDAGNLNEAVKRNTDAISKAMENKDHGCKDAYDKAAQELRNMDWDQYIQDAITNGTKAK

>WP_349335710.1:173-326 hypothetical protein [Delftia sp. DS1230]

PKRHKDCGKKVPYNDKKSLKGSGLEKDHTPSGAALEKAAENKINELRDSGVKISDAQASEIRKSVRNNAPTIAVPPDIHA

EGQTWRYKNTPERISQDAGNLNEAVKRNTDAISKAMENKDHGCKDAYDKAAQELRNMNWDQYIQDAITNGTKAK

>WP_279495373.1:173-326 hypothetical protein [Delftia tsuruhatensis]

PKRHKDCGKKVPYNDKKSLKGSGLEKDHTPSGAALEKAAESKINELRDSGVKISDAQASEIRKSVRNNAPTIAVPPDIHA

EGQTWRYKNTPERISQDAGNLNEAVKRNTDAISKAMENKDHGCKDAYDKAAQELRNMDWDQYIQDAITNGTKAK

>WP_092740276.1:166-322 MULTISPECIES: hypothetical protein [Pseudomonadota]

GNGGGKDTGKPKRHKDCGKKVPYNDRKAVKGSGLEKDHTPSGAALERAAENKIQDMRDSGVFIDDAKAKKIKNAVRNNAP

TIAIPPDIHAEGDTWRHKNNQTKIDADAKDLKGAVKRNTEKISKAMEGKDHGCKDAYDIAAQELRDIDWDKYIEDAV

>WP_081483853.1:182-329 MULTISPECIES: hypothetical protein [Paracidovorax]

CGKKVPYNDKKSLKGSGLEKDHTPSGAALEQAADKKIKELRNNGIDIDEDQAKKIRNAVRNQAPTIAIPPDIHAEGDTWR

HKNTPEKIRKDASDLKDAVKRNTEKISKAMEGKDHGCKEAYDKAAEELRNMDWDKYIQDAIDNVTKAQ

>WP_284429989.1:181-328 hypothetical protein [Acidovorax sp. SUPP950]

DCGKKVPYNDKKSLKGSGLEKDHTPSGAALERAAERKIKDLREGGMVIDDAQAQKIRNAVRNQAPTIAIPPDIHAEGDTW

RHKNSPETVKKDAANLKDAVRRNTEKISKAMEGKDHGCKEAYDKAAEELRNMDWDKYIQDAIDNVVKA

>WP_082091343.1:182-329 hypothetical protein [Paracidovorax citrulli]

CGKKVPYNDKKSLKGSGLEKDHTPSGAALEKAADMKIEELRESGMVIDDAQAKKIRNAVRNQAPTIAIPPDIHSEGDTWR

HKNTPDKVNKDASNLKDAVKRNTEKISKAMEGKDHGCKEAYDKAAEELRNMDWDKYIQDAIDNVVKAQ

>WP_249343487.1:37-177 MULTISPECIES: hypothetical protein [Delftia]

NDKKSLKGSGLEKEHPPSGASLEKSAENKINELRDSVVKISDAQASEIRKSVRNNAPTIAVPPDIHAEGQNWRYKNTPGR

ISQDTENLNEAVKRIQMPSPMIMENEDHGCKNTYDKAAQELRNLDCDQHIKNAITNVTKAK

>WP_279495364.1:22-162 hypothetical protein [Delftia tsuruhatensis]

NDKKSLKGSGLEKEHPPSGASLEKSAENKINELRDSVVKISDAQASEIRKSVRNNAPTIAVPPDIHAEGQNWRYKNTPGR

ISQDTENLNEAVKRIQMPSPMIMENEDHGCKNTYDKAAQELRNLDCDQHIKNAITNVTKAK

>MBV5299542.1:706-852 hypothetical protein [Rhodoferax sp.]

HKDCGKVVIYKSKDVQHTGLEKDHTPSGGALEIATMEKLQPYFENKSLKSSQAAEIVRFVKNNMPTIAIPPDVHKEGRTY

KGKNTLKQRTDDAQDLNEAAKRDIKAIQESMKSKKHGCSKAYAIAAKEVLAFDFEAYIDETIVSFTK

>WP_306698362.1:78-216 hypothetical protein [Treponema endosymbiont of Eucomonympha sp.]

HELCGKRSTYNKAPKKLGELNADHVPSGGALKQAAKDLLVEKGIWDSLSEKQQKSVLNRVYNNAPTITVPEDIHKEGRTY

GSKNKPLIQGDSKGLKDAFKRDTEAIRKAMEGKDHGCLEEYMRSVEELKDFDFDKYVED

>WP_012205696.1:179-317 MULTISPECIES: hypothetical protein [Delftia]

HELCGKRSTYNKAPKKLGELNADHVPSGGALKQAAKDLLVEKGIWDSLSEKQQKSVLNRVYNNAPTITVPEDIHKEGRTY

GSKNKPLIQGDSKGLKDAFKRDTEAIRKAMEGKDHGCLEEYMRSVEELKDFDFDKYVED

>WP_197943477.1:179-317 hypothetical protein [Delftia acidovorans]

HELCGKRSTYNKAPKKLGELNADHVPSGGALKQAAKDLLVEKGIWDSLSEKQQKSVLNRVYNNAPTITVPEDIHKEGRTY

GSKNKPLIQGDSKGLKDAFKRDTEAIRKAMEGKDHGCLEEYMRSVEELKDFDFDKYVED

>WP_252980586.1:66-199 hypothetical protein [Delftia acidovorans]

CGKRSTYNKAPKKLGELNADHVPSGGALKQAAKDLLVEKGIWDSLSEKQQKSVLNRVYNNAPTITVPEDIHKEGRTYGSK

NKPLIQGDSKGLKDAFKRDTEAIRKAMEGKDHGCLEEYMQSVEELKDFDFDKYV

>WP_310635572.1:182-317 hypothetical protein [Delftia acidovorans]

CGKRSTYNKAPKKLGELNADHVPSGGALKQAAKDLLVEKGIWDSLSEKQQKSVLNRVYNNAPTITVPEDIHKEGRTYGSK

NKPLIQGDSKGLKDAFKRDTEAIRKAMEGKDHGCLEEYMQSVEELKDFDFDKYVED

>WP_082848130.1:182-315 MULTISPECIES: hypothetical protein [Delftia]

CGKRSTYNKAPKKLGELNADHVPSGGALKQAAKDLLVEKGIWDSLSEKQQKSVLNRVYNNAPTITVPEDIHKEGRTYGSK

NKPLIQGDSKGLKDAFKRDTEAIRKAMEGKDHGCLEEYMQSVEELKDFDFDKYV

>WP_325278201.1:246-397 hypothetical protein [Tahibacter sp.]

GGKVKKQKPHKDCGKFGRYKNMPKQKGVINADHVPSGAALKKAFQEKLEEMGIWDDLSKTQRESVLNHLYREAPTIVVPE

DVHKEGRTYGGKNTGKQSTKDAGNLRKAVKDDTDAIQRSMDTKDHGCSEAYRKAAEEMRNFDFDGLFDEIIR

>WP_178952853.1:210-373 hypothetical protein [Pseudomonas reactans]

VKRAQRDGPEKNKVRRQRPDPRCGKVSTYRKAPKEKGLLNADHIPSGAALKMYAERRLKEQGIWSSLDSQEQRKILNRVY

DNAPTITIPEDVHKEGRTYGNRNKPLFREDGKSKESLSAAFERDSKQIQKSMDAKDHGCTEAFAKAVEELRGYDYDKFID

SIIK

>HAF2404054.1:202-340 hypothetical protein [Salmonella enterica]

CGNKEKYTNNYKEKKVMNADHAPSGAALKKAAENKLKEMGIWNKLSAEERKSILNKVYNDAPTISIPEDIHKKGRTYGGK

NKSTQSSMDAKDLNGAFKKDTDLIQELMDASEKGCSEAYKKSVDELSKMDWNKYINDTV

>WP_086077371.1:210-374 hypothetical protein [Bordetella genomosp. 13]

KKDEDDGGKSQGKKKPDCGRIGVYKNKKDFDNTDTNWDHVPSGRALEQAAENKLKAMPGQGNKSMWSSLTAAQRKKVLNA

ARNEAYTINIPADVHKNSSLTWGSRNKPRYGPDAGNLREAMMRDMDALEEAMKNSDHPCRKQYSAARRAMQRIDPDKHLQ

DIINI

>WP_066127947.1:218-382 hypothetical protein [Bordetella ansorpii]

KKDEEDGGKSEGKKKPDCGQVGVYKNKKDFDNTDTNWDHVPSGRALEQAAENKLKAMRGQGKKSMWDSLTAAQRKKVLNA

ARNEAYTINIPADVHKNSSLTWGSRNKPRYGPDAGNLREAMLRDMDALEEAMKNSDHPCRKQYSAARRAMQRIDPDKHLQ

DIINI

>WP_127960406.1:346-484 hypothetical protein [Serratia microhaemolytica]

QTKTHPDCGKVSKYYKAPKKLGKLNADHVPSGAALKEATKNKLKELDIDLLDSELERVLDSVYRNAETITIPEDIHKEGR

TYGNKNKGLYKGDSKDLKGAFKKDTASIQKVMDTKEHGCSEAYAKAVEQLSKIDYDEFI

>WP_237736819.1:179-266 hypothetical protein [Delftia acidovorans]

HRDCGKRSTYNKAPKKIGELNAAHVPSGGALKQAAKDLLVENGIWDSLSEKQQKSVLNRVHNNAPTITVPEDIHKEGRTY

GSKNKPLI

>WP_195795437.1:156-319 hypothetical protein [Roseateles sp. DAIF2]

KDEGADGGKVKPKPKPECGQRGPYKDRDDHDNKGFNWDHVPSKAALLAKAEEIKGDLLSAAEKTAIIEGAPTIAIPEDLH

RKHSETYGGRQNQTVDGQKRILNDAGNLQKAAKENTDNILKHVDEFDPGCRGAYEEAAKAFSAITNEEWDKWLDQTMKTA

RKKK

>MCP4532358.1:179-273 hypothetical protein [Delftia sp.]

HELCGKRSTYNKAPKKLGELNADHVPSGGALKQAAKDLLVEKGIWDSLSEKQQKSVLNRVYNNAPTITVPEDIHKEGRTY

GSKNKPLIQGDSKGL

>WP_307594588.1:248-383 hypothetical protein [Variovorax paradoxus]

CGQQGPYKDRNNHDNAGMNWDHVPSQAALLKAARQTKGAALTPAEIKAVVDNAPTIAIPAQLHQKHSETYGGRQHQSVDG

VKRPARDASDLQRAAKENTDKILDAIDKYDPGCKGAYRDAARKITDMTNDDWKKWL

>WP_155736590.1:250-385 hypothetical protein [Variovorax paradoxus]

CGQQGPYKDRNNHDNAGMNWDHVPSQAALLKAARQTKGAALTPAEIKAVVDNAPTIAIPAQLHQKHSETYGGRQHQSVDG

VKRPARDASDLQRAAKENTDKILDAIDKYDPGCKGAYRDAARKITDMTNDDWKKWL

>WP_155419781.1:223-385 hypothetical protein [Variovorax paradoxus]

GKDAETGGGGSKNNDTQVKKKVKPKPKCGQQGPYKDRNNHDNAGMNWDHVPSQAALLKAARQTKGAALTPGEITAIVDNA

PTIAIPTQLHQKHSETYGGRQHQTVDGMKRPARDASDLQRAAKENTDKILDAIDEYDPGCKGSYRNAAKKITDMTNDDWK

KWL

>WP_224986431.1:7-82 hypothetical protein [Delftia acidovorans]

KAAKSVLNRVYNNAPTITAPEDIHKECRTYGSKNKPFIQGYSKGMKDAFKRNTEAIRKAMEGKDHGYLEEYMQSVE

>WP_067271579.1:170-308 hypothetical protein [Mitsuaria sp. 7]

KPKCGQTGPYKDRDSHDNAGFNWDHVPSKAALLKRAEELAGDVLSKDQIKAIIENAPTIAIPDKLHQKHSETYGGRQNQI

VDDERRIKRDSGNLQRAAKENTDALLKHVDDFDPGCKGAYSEAAKAFSAITNDDWDKWL

>WP_093200219.1:258-398 hypothetical protein [Variovorax sp. YR750]

CGQKGPYKDRSKHDNEGMNWDHIPSQKALLERAAEIKGAPLTKAEKAAIVDNAPTIATPTELHRDHSESFGGRQHQKIDG

VRRPTRDAQDLQRAAKENTEKILQEIDKYDPGCKGKYREAAEEITKMTHDQWTKWLKQAMK

>WP_175891890.1:1380-1502 RHS repeat-associated core domain-containing protein [Burkholderia cepacia]

ERDHVPSGAAQKKHAETMLRNLGIWDNLGPKEQKNVLDHVYNDAKSVTIPKPPHVGGRTWGHHNTDSQIALDSRDLNLAF

SKDVEAIQKQMAHLPPECQRAYTRAVFEMRNHDAYNHIESSIR

>WP_301839668.1:797-919 RHS repeat-associated core domain-containing protein [Burkholderia cepacia]

ERDHVPSGAAQKKHAETMLRNLGIWDNLGPKEQKNVLDHVYNDAKSVTIPKPPHVGGRTWGHHNTDSQIALDSRDLNLAF

SKDVEAIQKQMAHLPPECQRAYTRAVFEMRNHDAYNHIESSIR

>MDX3775808.1:66-161 hypothetical protein [Chromatiaceae bacterium AAb-1]

KREQVGDGLEHDHIPSFAALKLAEENKLGRRLTPDEERVLYNNAVAVEVPRDIHQQSRTYGGRNTKLQIEQDAIDLCGAI

CRDTNVLRQNLLNKGY

>KVU52750.1:1348-1470 hypothetical protein WK70_28760 [Burkholderia cepacia]

ERDHVPSGAAQKKHAETMLRNLGIWDNLEPKEQKNVLDHVYNDAKSVTIPKPPHVGGRTWGHHNTDSQIALDSRDLNLAF

SKDVEAMQKQMAHLPPECQRAYTRAVFEMRNHDAYNHIESSIR

>WP_207761306.1:285-398 VENN motif pre-toxin domain-containing protein, partial [Lelliottia amnigena]

DEIQAVKPLDVGSYRELKDRSVVGDGLEHDHIPSFAAIRQAKENELGRKLTPAEEKTLYNNATTIEVPKDVHQAGPTYGG

KNTPSQVKQDAINLCGAECRDTDALRKNMLDRGY

>WP_217997176.1:200-294 VENN motif pre-toxin domain-containing protein, partial [Lelliottia amnigena]

RSVVGDGLEHDHIPSFAAIRQAKENELGRKLTPAEEKTLYNNATTIEVPKDVHQAGPTYGGKNTPSQVKQDAINLCGAEC

RDTDALRKNMLDRGY

>WP_336994181.1:175-288 VENN motif pre-toxin domain-containing protein, partial [Lelliottia amnigena]

DEIQAVKPLDVGSYRELKDRSVVGDGLEHDHIPSFAAIRQAKENELGRKLTPAEEKTLYNNATTIEVPKDVHQAGPTYGG

KNTPSQVKQDAINLCGAECRDTDALRKNMLDRGY

>WP_301825669.1:1380-1502 RHS repeat-associated core domain-containing protein [Burkholderia cepacia]

ERDHVPSGAAQKKHAETMLRNLGIWDNLDPKEQKNVLDHVYNDAKSVTIPKPPHVGGRTWGHHNTDSQIALDSRDLNLAF

SKDVEAMQKQMAHLPPECQRAYTRAVFEMRNHDAYNHIESSIR
