# Supplementary material for: A broad-spectrum anti-fungal effector dictates bacterial-fungal interkingdom interactions
Source: PLoS Pathog. 2025 Oct 27;21(10):e1013598. doi: 10.1371/journal.ppat.1013598 (PMC12574953; doi:10.1371/journal.ppat.1013598)
Supplement: S2 Data — (DOCX) [file ppat.1013598.s015.docx]

>NoLS

MATKRPRPPEAQTRNARVKPN

>WP_208942746.1

MATKRPRPPEAQTRNARVKPN

>WP_274340481.1

MASKRPRPPEAETRNARVKPN

>WP_284408693.1

MASKRPRPPEAETRNARVKPN

>WP_228316160.1

MASKRPRPPEAETRNARVKPN

>WP_092740276.1

MASKRPRPPEAETRNARVKPN

>WP_284429989.1

MAPKRPRPPEAETRNARIKPN

>WP_013802343.1

MATRKKQLPAAQSQTAQVKPN

>WP_279495373.1

MATRKKQLPAAQSQTAQVKPN

>WP_349335710.1

MATRKKQLPAAQSQTAQVKPN

>WP_279213078.1

MATRKKQLPAAQSQTAQVKPN
