# Supplementary material for: A broad-spectrum anti-fungal effector dictates bacterial-fungal interkingdom interactions
Source: PLoS Pathog. 2025 Oct 27;21(10):e1013598. doi: 10.1371/journal.ppat.1013598 (PMC12574953; doi:10.1371/journal.ppat.1013598)
Supplement: S3 Data — (DOCX) [file ppat.1013598.s016.docx]

>WP_011795249.1 hypothetical protein [Paracidovorax citrulli]

MATKRPRPPEAQTRNARVKPNDYPSPGPIGNTTGIVVPDTAPNPHGNSAPPIKPTEPEKSWWSRWGSDVVHTGLDVVGLI

PGVGEIADGANALIYLAEGDKVNAAISAAAMIPGAGMAATGAKYGKKAAGAAAEAIGKKTAREAEEALVKREAKEAEEAA

AKKAEGNGGGKDKAKPKKHKDCGKKVPYNDKKSLKGSGLEKDHTPSGAALELAAQNKIDELIANGARITEKQQKAILNSV

RNNAPTIAIPPDIHALGDTWRAKNKPHVIEKDAANLKDAVARNTKTISEAMKNKDHGCKEAYDKAAEELRNMDWEKYIDE

AIQVGTKAKVKK

>WP_208942746.1 hypothetical protein [Paracidovorax avenae]

MATKRPRPPEAQTRNARVKPNDYPSPGPIGNTTGIVVPDTAPNPHGNSAPPIKPTEPEKSWWSRWGSDVVHTGLDIVGLI

PGVGEVADGANALIYLAEGDKVNAAISAAAMIPGAGMAATGAKYGKKVAGAAAEAIGKKTAREAEEALVKREVKEAEEAA

AKKAEGNGGGKDKAKPKKHKDCGKKVPYNDKKSLKGSGLEKDHTPSGAALELAAQNKIDELIANGARITEKQQKAILNSV

RNNAPTIAIPPDIHALGDTWRAKNKPHVIEKDAANLKDAVARNTKTISEAMRNKDHGCKEAYDKAAEELRNMDWDKYIDD

AIQVGTKAKVKK

>WP_274340481.1 hypothetical protein [Xanthomonas campestris]

MASKRPRPPEAETRNARVKPNNYPSPGPIGNTTGIVVPDTAPNPHGNSAPPIKPTEPEKSWWSRWGSDVVHTGLDVVGLI

PGAGEIADGANALIYLAEGDKVNAAISAAAMIPGAGMASTGAKYGKKAADAAVEAVGKKTAREAEEALAKREAKEAEEAA

AKKAEGNGGGKDTGKPKRHKDCGKKVPYNDRKSLKGSGLEKDHTPSGAALELAAQNKIDELVASGARITEDQQKAIVNSV

RNNAPTIAVPPDIHAQGDTWKHKNTPANIERDAGNLNDAVKRNTNAISKAMEGKDHGCKEAYDKAAEELRNMDWNKFIDG

AIEAGTKSKVKK

>WP_284408693.1 hypothetical protein [Acidovorax sp. SUPP2539]

MASKRPRPPEAETRNARVKPNNYPSPGPIGNTTGIVVPDTAPNPHGNSAPPIKPTEPEKSWWSRWGSDVVHTGLDVVGLI

PGAGEIADGANALIYLAEGDKVNAAISAAAMIPGAGMASTGAKYGKKAADAAVEAVGKKTAREAEEALAKREAKEAEEAA

AKKAEGNAGGKDTGKPKRHKDCGKKVPYNDRKSLKGSGLEKDHTPSGAALEHAAELKILELRESGMRINEIQAKKILNAV

RNQAPTIAIPPDIHSDGDTWRHKNTPEKIKKDASDLKGAVKRNTEKISKAMEGKDHGCKDAYDKAAEELRNMDWDKYIQD

AIDSAIKAK

>WP_228316160.1 hypothetical protein [Xanthomonas sp. NCPPB 1067]

MASKRPRPPEAETRNARVKPNNYPSPGPIGNTTGIVVPDTAPNPHGNSAPPIKPTEPEKSWWSRWGSDVVHTGLDVVGLI

PGAGEIADGANALIYLAEGDKVNAAISAAAMIPGAGMASTGAKYGKKAADAAVEAVGKKTAREAEEALAKREAKEAEEAA

AKKAEGNAGGKDTGKPKRHKDCGKKVPYNDRKSLKGSGLEKDHTPSGAALEHAAELKILELRESGMRINEIQAKKILNAV

RNQAPTIAIPPDIHSDGDTWRHKNTPEKIKKDASDLKGAVKRNTEKISKAMEGKDHGCKDAYDKAAEELRNMDWDKYIQD

AIDNAIKAK

>WP_092740276.1 MULTISPECIES: hypothetical protein [Pseudomonadota]

MASKRPRPPEAETRNARVKPNNYPSPGPIGNTTGIVVPDTAPNPHGNSAPPIKPTEPEKSWWSRWGSDVVHTGLDVVGLI

PGAGEIADGANALIYLAEGDKVNAAISAAAMIPGAGMASTGAKYGKKAADAAVEAVGKKTAREAEEALAKREAKEAEEAA

AKKAEGNGGGKDTGKPKRHKDCGKKVPYNDRKAVKGSGLEKDHTPSGAALERAAENKIQDMRDSGVFIDDAKAKKIKNAV

RNNAPTIAIPPDIHAEGDTWRHKNNQTKIDADAKDLKGAVKRNTEKISKAMEGKDHGCKDAYDIAAQELRDIDWDKYIED

AVNNVIKGK

>WP_284429989.1 hypothetical protein [Acidovorax sp. SUPP950]

MAPKRPRPPEAETRNARIKPNDYPAPGPIGNTTGIVVPDTAPNPHGNSAPPIKPTETEKSWWSRWGSDVVHTGLDVIGLI

PGAGEIADGANALIYLAEGDKVNAAISAAAMIPGAGMAATGAKYGKKAVGAAAEAVGKKTAREAEEALAKREAKEAEEAA

AKKAEGNGGGKDKGKPKKHKDCGKKVPYNDKKSLKGSGLEKDHTPSGAALERAAERKIKDLREGGMVIDDAQAQKIRNAV

RNQAPTIAIPPDIHAEGDTWRHKNSPETVKKDAANLKDAVRRNTEKISKAMEGKDHGCKEAYDKAAEELRNMDWDKYIQD

AIDNVVKATS

>WP_013802343.1 hypothetical protein [Delftia sp. Cs1-4]

MATRKKQLPAAQSQTAQVKPNAYPSPGPIGNTSGLVIPDTAPNLHGATAPAIKPVAQEKSWWQRWGSDVVHTGLDVVGLI

PGVGEIADGANALIYLAEGDKVNAALSAAAMIPGAGMAATGAKYGKKAAAAATEAVGKKIGREASEEAAGKAAKETGAKK

ADGKGGGKDKGKPKRHKDCGKKVPYNDKKSLKGSGLEKDHTPSGAALEKAAENKINELRDSGVKISDAQASEIRKSVRNN

APTIAVPPDIHAEGQTWRYKNTPERISQDAGNLNEAVKRNTDAISKAMENKDHGCKDAYDKAAQELRNMDWDQYIQDAIT

NGTKAKK

>WP_279495373.1 hypothetical protein [Delftia tsuruhatensis]

MATRKKQLPAAQSQTAQVKPNAYPSPGPIGNTSGLVIPDTAPNLHGATAPAIKPVAQEKSWWQRWGSDVVHTGLDVVGLI

PGVGEIADGANALIYLAEGDKVNAALSAAAMIPGAGMAATGAKYGKKAAAAATEAAGKKIGREASEEAAGKAAKETGAKK

ADGKGGGKDKGKPKRHKDCGKKVPYNDKKSLKGSGLEKDHTPSGAALEKAAESKINELRDSGVKISDAQASEIRKSVRNN

APTIAVPPDIHAEGQTWRYKNTPERISQDAGNLNEAVKRNTDAISKAMENKDHGCKDAYDKAAQELRNMDWDQYIQDAIT

NGTKAKK

>WP_349335710.1 hypothetical protein [Delftia sp. DS1230]

MATRKKQLPAAQSQTAQVKPNAYPSPGPIGNTSGLVIPDTAPNLHGATAPAIKPVAQEKSWWQRWGSDVVHTGLDVVGLI

PGVGEIADGANALIYLAEGDKVNAALSAAAMIPGAGMAATGAKYGKKAAAAATEAAGKKIGREASEEAAGKAAKETGAKK

ADGKGGGKDKGKPKRHKDCGKKVPYNDKKSLKGSGLEKDHTPSGAALEKAAENKINELRDSGVKISDAQASEIRKSVRNN

APTIAVPPDIHAEGQTWRYKNTPERISQDAGNLNEAVKRNTDAISKAMENKDHGCKDAYDKAAQELRNMNWDQYIQDAIT

NGTKAKK

>WP_279213078.1 hypothetical protein [Delftia acidovorans]

MATRKKQLPAAQSQTAQVKPNAYPSPGPIGNTSGLVIPDTAPNLHGATAPAIKPVAQEKSWWQRWDRDVVHTGLDVVGLI

PGVGEIADGANALIYLAEGDKVNAALSAAAMIPGAGMAATGAKYGKKAAAAATEAAGKKIGREASEEAAGKAAKETGAKK

ADGKGGGKDKGKPKRHKDCGKKVPYNDKKSLKGSGLEKDHTPSGAALEKAAENKINELRDSGVKISDAQASEIRKSVRNN

APTIAVPPDIHAEGQTWRYKNTPERISQDAGNLNEAVKRNTDAISKAMENKDHGCKDAYDKAAQELRNMDWDQYIQDAIT

NGTKAKK

>WP_199140333.1 hypothetical protein, partial [Delftia sp. ASV31]

PSPGPIGNTSGLVIPDTAPNLHGATAPAIKPVAQEKSWWQRWGSDVVHTGLDVVGLIPGVGEIADGANALIYLAEGDKVN

AALSAAAMIPGAGMAATGAKYGKKAAAAATEAAGKKIGREASEEAAGKAAKETGAKKADGKGGGKDKGKPKRHKDCGKKV

PYNDKKSLKGSGLEKDHTPSGAALEKAAENKINELRDSGVKISDAQASEIRKSVRNNAPTIAVPPDIHAEGQTWRYKNTP

ERISQDAGNLNEAVKRNTDAISKAMENKDHGCKDAYDKAAQELRNMDWDQYIQDAITNGTKAKK

>WP_197943477.1 hypothetical protein [Delftia acidovorans]

MATRKKQLPAAQSQTAQVKPNAYPSPGPIGNTSGLVIPDTAPNLHGATAPAIKPVAQEKSWWQRWGSDVVHTGLDVVGLI

PGVGEIADGANALIYLAEGDKVNAALSAAAMIPGAGMAATGAKYGKKAAAAATEAVGKKAGREAAEAGSQKAAKEAEEAA

AKKADGQGGGKDKGKPKKHELCGKRSTYNKAPKKLGELNADHVPSGGALKQAAKDLLVEKGIWDSLSEKQQKSVLNRVYN

NAPTITVPEDIHKEGRTYGSKNKPLIQGDSKGLKDAFKRDTEAIRKAMEGKDHGCLEEYMRSVEELKDFDFDKYVEDMAL

SHKAVKPKLPST

>WP_310635572.1 hypothetical protein [Delftia acidovorans]

MATRKKQLPAAQSQTAQVKPNAYPSPGPIGNTSGLVIPDTAPNLHGATAPAIKPVAQEKSWWQRWGSDVVHTGLDVVGLI

PGVGEIADGANALIYLAEGDKVNAALSAAAMIPGAGMAATGAKYGKKAAAAATEAVGKKAGREAAEAGSQKAAKEAEEAA

AKKADGQGGGKDKGKPKKHKDCGKRSTYNKAPKKLGELNADHVPSGGALKQAAKDLLVEKGIWDSLSEKQQKSVLNRVYN

NAPTITVPEDIHKEGRTYGSKNKPLIQGDSKGLKDAFKRDTEAIRKAMEGKDHGCLEEYMQSVEELKDFDFDKYVEDMAL

SHKAVKPKLPST

>WP_237736819.1 hypothetical protein [Delftia acidovorans]

MATRKKQLPAAQSQTAQVKPNAYPSPGPIGNTSGLVIPDTAPNLHGATAPAIKPVAQEKSWWQRWGSDVVHTGLDVVGLI

PGVGEIADGANALIYLAEGDKVNAALSAAAMIPGAGMAATGAKYGKKAAAAATEAVGKKAGREAAEAGSQKAAKEAEEAA

AKKADGQGGGKDKGKPKKHRDCGKRSTYNKAPKKIGELNAAHVPSGGALKQAAKDLLVENGIWDSLSEKQQKSVLNRVHN

NAPTITVPEDIHKEGRTYGSKNKPLIQGARKD

>WP_245711377.1 hypothetical protein [Paracidovorax valerianellae]

MASKRPRPPQADTRNARVKPNDYPSPGPIGNTTGIVVPDTAPNPHGNSAPPIKPTEPEKSWWSRWGSDVVHTGLDVVGLI

PGAGEIADGANALIYLAEGDKVNAAISAAAMIPGAGMAATGAKYGKKAVGAAAEAVGKKTAREAEEALAKREAKEAEEAA

AKKAEGNGGGKDKGNPRCVLRPYSPDTCKAEGRTGHHVVPDRVFRTGPRGSPHPFGITEAEGLVICVDGANVSRSKEHGK

IHAIYDPMERAAGLAGNPPGTAKLGVLEAAGALSVGKITGCNPILLEAQLRAFHQLKGLNVDTVVRADPSGKIPIDFSKI

GPGKTTQGGPLR

>WP_325278201.1 hypothetical protein [Tahibacter sp.]

MSFQPGWYSTPSLGPNSFQYFKTPNLVTAQGYFDGPNQILQWGVEQYPNGQPGQWTADMQALQKADPGDWWDNPKTGRVK

PNNQRKPGPIANTQGLIVPQTSPNLSGSSSAPTPKAPEGQEEKGWWKSWGSDVTHGVLDVIGLVPVVGEVANGVGALVYV

AEGDYVNAALDAAAMWPAGGQAATAAKYGKKGVGAAIEQGEKKLAREAEEAAREKLAREARERAEKEAREKAEKEAAEKK

AKEAEGGKVKKQKPHKDCGKFGRYKNMPKQKGVINADHVPSGAALKKAFQEKLEEMGIWDDLSKTQRESVLNHLYREAPT

IVVPEDVHKEGRTYGGKNTGKQSTKDAGNLRKAVKDDTDAIQRSMDTKDHGCSEAYRKAAEEMRNFDFDGLFDEIIRDNK

YIKKITGS

>WP_224986429.1 hypothetical protein [Delftia acidovorans]

MATRKKQLPAAQSQTAQVKPNAYPSPGPIGNTSGLVIPDTAPNLHGATAPAIKPVAQEKSWWQHWGSDVVHTGLDVMGLV

PGVGEIADGPNALIYLAEGDKVNAALSAAAMIPGAGMAATGSKYGKKAAAAATEAVGKKSGREAAEAGSQKAAKEAEEAA

AKKADGQGGGKDKGKPKKHRDCGKRSTYNKHLKNLKN

>WP_341645986.1 hypothetical protein [Thauera sp. SDU_THAU2]

MGNTQGIIVPDTGPNPHGNSAPPIQATAKETGWWGRWGSAVAHGVLDVVGLIPVVGEVADGANALIYLAEGDTVNAALSA

AAMIPGAGMAATGAKYAKKAGTAVEGAIKGGRETAEHAGKKEAGEAAAERGTKKAQGSGGGKDKGKPKEKLKCGQRGSYG

DLKKQSGDGRFDRDHIPSKAALLERARQLNDGEKLTPAQTKAITDWGDAIAIPREAHQQYSPTYGGRNNPAADAKDLASA

AKRDVDTILEHIDEYDADGGCKKAYKQASKKITDMTNADYARELEKLLK

>WP_157659189.1 polymorphic toxin type 15 domain-containing protein [Thauera butanivorans]

MDELEEAMSGKRPTVSAQVKPNAQPTPGPMGNTQGIIVPDTGSNPHGNSAPPIQATAKETGWWGRWGSAVTHGVLDVVGL

IPVVGEIADGANALIYLAEGDTVNAALSAAAMVPGLGMAATGAKVGKRAVGAAVEGTGKAVGKETAQQASQQGAKKTQGS

GGGKDRARGRRQRVKCFCPQDRAKGGRDEYDRQLKHQQDGINAMSVDEYLAQRGGFTGTNPCTGQSVAKAPGRDLSVTRK

AKNQRQKEQTQRYSDQFRDQGLGRSDAKRMGSAKAKRERDQTNPLHNQDMVAGGRDVIGDNGVLSDADFGFSDTNQHIGS

QWRGDRINSMDAEACRRKQAGEGNEKMNVELRACGKREARAAGCKPSPRK

>WP_175525996.1 hypothetical protein [Paracidovorax konjaci]

MATRKRGPDDDPSDAAQPRAARIKPNSWSSPGPIGNTTGLVVPETAPNPHFASAPAIKPTETEKGWWASWGSDVLHTGLD

VVGLVPGFGEVADGANALIYLAEGDKVNAAISAAAMIPGLGAGATATKYGKKAAEMGAEALGKTAGREAAEAAGQKTARE

AEEAAAKKSTDNGNPQGGNSKGNPRCILRPYKPDTCKAEGRTGHHVVPDRVFRTGARGSSHPFGVSEDDGLVICVDGANL

NSSKEHGKIHKIYDQAERALGKAGKPPGTAPLGLLEATGALAVGKVTGCNPLLLEAQLRAYHELKGLGPGTIVRADPSGK

LPIDFSNIGVGNTTPNLPRK

>WP_272548440.1 hypothetical protein [Acidovorax sp. NCPPB 3576]

MATRKRGPDDDPSDAAQTPAARIKPNNWSSPGPIGNTTGLVVPETAPNPHFDSAPPIKPTEPEKGWWTSWGSNVLHTGLD

AVGLVPGFGEVADGTNALIYLAEGDKVNAAISAVAMIPGLGAGATATKYGKQVAEAGAEALGKTAGREAAEAAGQKAAKE

AEERAAREAEEAAAKKTTDKGNPEGGKVDGSRQFKGKCGEWLAKMDLFDAGYTEIMEVQNKSGHGIDVMARNPNGDIKVL

EVKTTDGTTAPSLKGDQAKLGGEGFTNSRLGRAANGRGHYKNSPDAIANGAKGLEWLLEAQELGKKVDYEKYDVFIDDPE

KGCIKKKAITNPWDIVEKKKKKK

>MFA9950003.1 hypothetical protein [Rhodocyclaceae bacterium Wk13]

MATRPRIPADLPPVTRRAGVHPNHYTRPGPLGNTQGKIVPDTRPNPGGESAPPIRPTEKEKSWWQRWGSELIHTGLDIIG

LIPVAGELADGANALIYLAEGDTVNAAISAAAMVPGAGMAATGAKIGKKAAGAVAEGAAKKAEREAAEELAEKAQKEAAK

KTEKELAERKAKSNDGGKDKGDGSICKSLAAAVFAKVPEVAKRFLDLFEDKLNLFSLTVKPDGSKGSPHPSLPKGSGTWH

GHLQQIEQKQRSLRDSIQEYDVAQCVQPKIPKSVRDLAWLPIPSKSGTAPNYPFSNFPK

>WP_178952853.1 hypothetical protein [Pseudomonas reactans]

MAEPINLAPVRVHAISVTVQPPPNSIVGPAEMLLQQGENGGVQIDVFWRDAMGNEVHESKEMQEKEYEALRASYLHSEQA

SLPPFSSFKSGNTIGRVVGETAPAAVPASQANPPIEAETEEEGITGEQILDGIQLGLDVVGLIPVVGEVADIASGVISLF

RGDYVGAGLSMLSAIPFVGYAGTAGKVTRYGTKMAEASAKAGKETAEKGVKRAQRDGPEKNKVRRQRPDPRCGKVSTYRK

APKEKGLLNADHIPSGAALKMYAERRLKEQGIWSSLDSQEQRKILNRVYDNAPTITIPEDVHKEGRTYGNRNKPLFREDG

KSKESLSAAFERDSKQIQKSMDAKDHGCTEAFAKAVEELRGYDYDKFIDSIIKTSKYVD

>WP_066127947.1 hypothetical protein [Bordetella ansorpii]

MAVEVDVGGPMAARVAPNGNNLMLYQRADGVSLNIWSPDNQTRLASGTISYDEYAKLRKDNPDLNLPDWQPPKIRQPGTN

ANGNTVGRVTGQSGGSGKPAAASNPPVKPETEGEGITSEQVLDGLQLGLDAVGLIPGVGEFADLANAGISALRGDFIGAG

LSLAAAIPFAGWGATTAKVGRKGMHAVEESAEAAAKRGSKEAPPPKEHPKDPKEQPKKKDEEDGGKSEGKKKPDCGQVGV

YKNKKDFDNTDTNWDHVPSGRALEQAAENKLKAMRGQGKKSMWDSLTAAQRKKVLNAARNEAYTINIPADVHKNSSLTWG

SRNKPRYGPDAGNLREAMLRDMDALEEAMKNSDHPCRKQYSAARRAMQRIDPDKHLQDIINITIRGL

>WP_325281365.1 hypothetical protein [Tahibacter sp.]

MSFQPGWYSTPSLGPNSFQYFQTPNLVTAQGYFDGSNQILQWGTEQFPNGQPGQWTPDMQALEKADAGDWWDNPKTGRVK

PNNQRKPGPIANTQGIIVPQTSPNLSGSTSAPTPKAPEGQEEKGWWKSWGSAVTHGVLDVVGMIPVVGEPANLIGAGIYA

LEGDYVSAAMDLAAMIPTGGQAATAAKYGRKVGNEVVEQAAKKTDDAAEVVAKQSDEAAEAAAKQSDEAAAAAGKNNSGG

KVKGKKKLDCGQMGKYGELQKFDAESAGMERDHIPPQAALSERAFQLANKQGLALSAAERKALAERVGRHALTISIPKDI

HSEGLTNSNPNNKNAAGDLQRIAREESRQHRENMRRSDKHHKCLGALQKATGKIGKITNQQYDKFLQNVIDDFVSGKRTT

NPWKGIL

>WP_279495364.1 hypothetical protein [Delftia tsuruhatensis]

MGREANEKAAGKAAKEKSAKNNDKKSLKGSGLEKEHPPSGASLEKSAENKINELRDSVVKISDAQASEIRKSVRNNAPTI

AVPPDIHAEGQNWRYKNTPGRISQDTENLNEAVKRIQMPSPMIMENEDHGCKNTYDKAAQELRNLDCDQHIKNAITNVTK

AKE

>MBK9083956.1 hypothetical protein [Sterolibacteriaceae bacterium]

MLIGDNAGAIRLGRVKPNNRPSPGPLGNTQGPILPETSPNASGAGKSAPPPRPPKAETGWWQRWGSDAVRTGLDIVGLIP

VVGEVADGANAMIYLAEGDKVNAASIAASMLPLGGQAAKWGKNGVDAAQAARTAGKAGVEIAATSGREAAEAVTSKLGNE

VAETAGGQARKSADNAASGGHVKKSPEELAAKMPRRSRSRKTLGGN

>WP_086077371.1 hypothetical protein [Bordetella genomosp. 13]

MALEVDIGGSMAARVAPNGNNLTLFQRADGVRLNVWSPDNQTELASGMLSYDEYAKLRKDNPELNLPDWQPPKVRQPGTN

ANGNTVGRVVGQAGGSGKPAAQSNPPVEPESDNEGVTGEKVLDGLQLGLDAVGLIPGVGEFADLANAGVSALRGDFVGAG

LSLAAAIPFVGWGATTAKVGRKGLHAVETTAEAAAKKGAKETPPPKERPKKDEDDGGKSQGKKKPDCGRIGVYKNKKDFD

NTDTNWDHVPSGRALEQAAENKLKAMPGQGNKSMWSSLTAAQRKKVLNAARNEAYTINIPADVHKNSSLTWGSRNKPRYG

PDAGNLREAMMRDMDALEEAMKNSDHPCRKQYSAARRAMQRIDPDKHLQDIINITIRGF

>WP_155736590.1 hypothetical protein [Variovorax paradoxus]

MVVTLNVGRGKIARALPNGNTLNVQPNADTVNVQEWIGQDGLGERISDRYLSYDEYDEYRRRNPDLDLPEYHPPKIRKPG

TNANGNTVGAVVGETPSAKVQAAQANPPVQAETPKEEGWWGSWGSSVVHTVLDVGGAIPVVGIFSDGLNAGIYAAEGDYA

NAAISGVSAAANLIPGGGAAAKAGKLAYAGGKMALKEAEKEVAEGLAKKTARELEEAAAKKTGKEAQTGGGGSKNNDTQV

KKKVKPKPKCGQQGPYKDRNNHDNAGMNWDHVPSQAALLKAARQTKGAALTPAEIKAVVDNAPTIAIPAQLHQKHSETYG

GRQHQSVDGVKRPARDASDLQRAAKENTDKILDAIDKYDPGCKGAYRDAARKITDMTNDDWKKWLKKTMKSASED

>WP_307594588.1 hypothetical protein [Variovorax paradoxus]

MTLNVGRGKIARALPNGNTLNVQPNADTVNVQEWIGQDGLGERISDRYLSYDEYDEYRRRNPDLDLPEYHPPKIRKPGTN

ANGNTVGAVVGETPSAKVQAAQANPPVQAETPKEEGWWGSWGSSVVHTVLDVGGAIPVVGIFSDGLNAGIYAAEGDYANA

AISGVSAAANLIPGGGAAAKAGKLAYAGGKMALKEAEKEVAEGLAKKTARELEEAAAKKTGKEAQTGGGGSKNNDTQVKK

KVKPKPKCGQQGPYKDRNNHDNAGMNWDHVPSQAALLKAARQTKGAALTPAEIKAVVDNAPTIAIPAQLHQKHSETYGGR

QHQSVDGVKRPARDASDLQRAAKENTDKILDAIDKYDPGCKGAYRDAARKITDMTNDDWKKWLKKTMKSASED

>WP_306698362.1 hypothetical protein [Treponema endosymbiont of Eucomonympha sp.]

MLLANDIGAPEGQLIAQRHLSDRFRGAGTQRSEAVGKKAGREAAEAGSQKATKEAEEAAAKKADGQGGGKDKGKPKKHEL

CGKRSTYNKAPKKLGELNADHVPSGGALKQAAKDLLVEKGIWDSLSEKQQKSVLNRVYNNAPTITVPEDIHKEGRTYGSK

NKPLIQGDSKGLKDAFKRDTEAIRKAMEGKDHGCLEEYMRSVEELKDFDFDKYVEDMALSHKAVKPKLPST

>WP_124961750.1 hypothetical protein [Variovorax beijingensis]

MVVTLNVGRGKIARALPNGNTLNVQPNADTVNVQEWIGQDGLGERISDSYLSYDEYDEYRRQNPDLDLPEYHPPKIRKPG

TNANGNTLGAVVGETPSAKVQAAQANPPVQAETPKEEGWWSSWGSSVVHTVLDVGGAIPVVGIFSDGLNAGIYAAEGDMA

NAAISGVSAAANLIPGGGAATKAGKLAYRGGKMALEKAEKELAEKLLKERLEKEAAERLAKEKLEKEAARKAEAGGGGGS

KKKDSQVKKKVKPKPKCGQKGPYKDRSKHDNEGMNWDHIPSQKALLERAAEIKGTPLTKAEKAAIVDNAPTIATPTALHQ

DHSESFGGRQHQKIDGVRRPTRDSQDLQRAAKENTEKILKEIDKYDPGCKGKYREAAEEITKMTHDQWTKWLKQAMKSAR

K

>MBV5299542.1 hypothetical protein [Rhodoferax sp.]

MSYAEPARENAALESVTYPDDRTREFFYDEAGKVIALVEPSGASYEIERDERGAIATIIDPLGRDIALVSDDAGALQGIV

FPDGSQEAFLFDADEQTAVRVHRNGSETHYKLDAQERIAQIVSGPEGATGFGYDAQGGLSFVEQNGQRISFKTEGDNTTE

EHGPDGKVNYEYDGDGRLTALNNPFGDRLQYAYDQDGRLANITLWDGRSLHIESNDEDLIARFQFPGGVVVHQHYGPALR

LSRREVQTADGSVQQVSYSYDDCARFTGDAEAGPQGHQRQISYDEDDHVVREVVNGHNQDYAFDVKGNMVQAGELALAVG

PMDEPLRFGDQSISYDPRGNMLLLPGAQGPLVCKYRHDGMLTECQGASGLVTFKYDALGRRVEKRSQNQIWRFGWTGQQL

LWEEYKAAEYATPVRRDYLFLPGTMQPLGFREQGRCYWLMTDARGAVDLALDDQGQVVWKARYDSFGRVELEVNKVRQPW

RMQGQYADDETGLYYNFARYYSPHLRTYLSLDPRWIELEASHYSYCRNDPWNRADPFGGLAPLVAIGVAGLVGAVVGGVT

AAVTGGDPLAGAVEGAVAGAVAGAGAAVAVVAGASAAVVLAAGVVASGVGAFVGQISEQAHKGDQFCISCALKGAAVAAA

TDLALLGLGKVPGVKQLVRSVGKKLVKLAKPLKTLVKKKLKDIKSVWNAARKPKKRSRRKLVQNPHKDCGKVVIYKSKDV

QHTGLEKDHTPSGGALEIATMEKLQPYFENKSLKSSQAAEIVRFVKNNMPTIAIPPDVHKEGRTYKGKNTLKQRTDDAQD

LNEAAKRDIKAIQESMKSKKHGCSKAYAIAAKEVLAFDFEAYIDETIVSFTKKST

>WP_185910697.1 hypothetical protein [Xanthomonas translucens]

MGVIETIPLRQSANAIGGKQTIMLQTGPNGGVIADIYGTDFQGNNLHQSYGELQEEKYNELSAFMLRKERATLPPFSHFK

TGNTLGRVVGESGASPVPATKANPPVKDETEEEGTFWAKNGEAILDGTQVALDVAGLIPVVGDVADVASGLVSLARGDYV

GAGLSFASAIPFAGWAASGAKAARRAMKSEKAAAKATKELTEEGAEKEAKDLAEKEAKDLAEERAKEAGEAAPGKKVKQK

KPLECGQKGPYGKLQEFDAESVGLERDHIPPQAALLQRALQIARSKEIDLDAGERRALKDRIGRHAFTVSVPKEIHGEGL

TNSNQNNKLGASDLQKTAHDEAHQHRENMRRSDKHHKCLGALQKATGEIGKITNKQYDQFLDKVLDDFVSGKRTTDPWKN

IL

>WP_228112003.1 GH-E family nuclease [Zoogloea sp. 1C4]

MSGKGKVKPNNQAAPGPIGNTQGLIVPQTQPNASGPGNSAPPPKPTPKEEGWWNRWGSDALHTTLDVVGLIPGVGEVADG

ANALVYLAEGDKVSAALSAAAMLPIGGQAATAAKMAKKGAQALEKQATKKAAEEAVEHSVKEAAEKAAKEKLEREAAEKA

KKEAQAAASGGRVEKISPRDDPRYRRGKFRKGKREKVWEDAKDSDGKVRDPKTGREMKENEPWDMGHKPGYEHRKHQESA

SRRDIDRKKFLDEYNDTTKYRPELPSSNRSHAGEIRTNDYFGD

>NLU13954.1 RHS repeat protein [Gammaproteobacteria bacterium]

MAKMPSGSVKPNHYGSAGSHANTTGMITPQATMGDATVVPPKPQPETQSWWGRWGDTIHIALDVVGMIPVIGEVADGANA

LLYLAEGDHVNAALSAAAMLPIGGQAATAAKAAKRGAEAAVATGKAAKTTAEAGVKAGREAGEAVAQAAGKKGTNGGTVS

GKGKDAKGNGQADKSKPNCDLTKPTGSKPVNAVQGSKVLAGSEDLDFYLPSPLPFVWQRTYVSSNALSGMLGQGWMTPLC

VQLESLDDETVYTDTEGRRFSFPVLAVGEEFYSRFEQLLLRRNLRHEYELIDAAGLHYVFARPFDRVEHSFALNRRQYTP

QATQRVGSPEDGRLLYLNGFFDRSHNCWAIRRTDDGLATEVIGSSAIGLKLDYLHLEGIGPRLAGVKRFWGDPAQPEQLL

PLVEYRYSPAGDLIEVLDDNAQSCRRFVWRNHMMVEHSQPGGVVARYEWNLHQPSGKVLRSYLSTGEEWTFAYQQNRTQV

TNQEGHVQWFEFDDEQELTGLIDPLGGVRRFDLDYFGHVRRDLQPGGVSTQYDYDERGQITRVTHADGHSLQLSWCSRWH

ELLSITDELGRVTRYAYDEQGRLTKITHADGNQTRLERNAQGLVTRMVDALGKTRTYEYNAQGLLTQQTDCSGHATQWHH

DHWGNITQMKDAQGQITRFDYQRINRASRLTQVQWPDGSRERFAYDNLGRLVGHQDALERVTRYQLDASGLPLEQENALG

DRVRYQYDSQGRLIELQNENRARYHFSWDALGRLVAEQGFDGRRQHYRYDAAGYLVEALDGVGSEERSANTPETHTQPIR

TRFQRDVLGRLLGKYSIKSQPGQRPLIQRDRYQYDAAGQLITARNAHARVVLHHDAMGNLLEEQLYQRGRGLSQLRHEYD

ELGNRLRTHLPDGRQLDTLRYGSGHVFAMELDGASLCDMERDSLHREISRSQGALRSRYQWDAMGRLLASRTERQQQSLV

GEPSSQGQQIARSYQYDAAGQLTAISDTRKGLTRYGYDELGRLLSAAHPFAVNEVFAFDPAHNLISQQQAEHNQKKRQTG

NRWSEEEWQAYVKANAHRADFDPWLTPEQGENDPRFWGEARPNRLSTWQDHRYQYDDFGNCTEKISGSANNSTQQSYEWD

AEHRLSKVWIERTHNGKTTREGWGYDYDPFGRRLAKYPLDAEANKARDTDEKAKALNQSRPWKSPQATYYGWDGHQLISE

QTGNQYQLYIYEPDSFVPLALVSSEINAKNQPTDELVEALKDSPLQWQQLQHQYPEHWVQVLNQQQKYRQKAGVV

>WP_092383255.1 hypothetical protein [Halopseudomonas salegens]

MSITLAPITVRAYRPVALPNGNAVIVDPRESSVLLETYSPQNPDTMLMSEALNYEQYAQMRAENPDIAFPELVKPVPADQ

QGQDSTQAQQGRIQPNNYPQPGPIGNTTGIIVPKTSAAPAGAPGNPSGGAETEAESGWWKSWGSAVTHGVLDVVGLVPVL

GIPADLTNAAIYAAEREYVDAGLSAAAAIPFFGWGATAVKRGRQVGNAVDATRAAQTGTEAAVTGGRQGAEAVGNASARN

TGSPSSTGGRVDGNTPPNNRDGQANRPCAC

>WP_195795437.1 hypothetical protein [Roseateles sp. DAIF2]

MGSYNTTGVLQKPATGAGKAEDANAGVKVETIEISKLDALQMLLDVVGLIPGLGAPADILNGLISAARGDWLGAGLSLLG

VVPIAGEAATAAKIAKNADRYTAGVRKVADEILPHLPEGVQRKLRDAIEKAEAKIDELAGREPKPKPKPDEAPKAKDEGA

DGGKVKPKPKPECGQRGPYKDRDDHDNKGFNWDHVPSKAALLAKAEEIKGDLLSAAEKTAIIEGAPTIAIPEDLHRKHSE

TYGGRQNQTVDGQKRILNDAGNLQKAAKENTDNILKHVDEFDPGCRGAYEEAAKAFSAITNEEWDKWLDQTMKTARKKK

>WP_021003711.1 hypothetical protein [Variovorax paradoxus]

MTVNLDIRPAFIRQVGGGGRLVGYNYADSVSMLYFPSDLGPAEKTWHMSHEQYEELRQAQPALELPELKPRKPGTNANGN

TLGSVVGETPSTAGKAKQANPEVKPETPKEEGWWSSWGSSVVHTVLDVGGAIPVVGIFSDGINAGIYAAEGDMANAAISG

VSAAANLIPGGGAATKAGKLAYRGGKMALEKAEKELAEKLLKERLEKEAAERLAKEKLEKEAAQKAEVGGGGGSKKKDSQ

VKKGKKPKCGGRATYGKQAGDFLGNEMERDHIPNVASLKDMAEKMLLEDGIDLTPAQRDSLFGKVGKNGQREGGKIYTHA

QTVAVPKEIHRASPTTGNHENYKDSKDLAGAARRDTKNHRDAIEKSDNHKDCAKALEEASKVIDKVSNDQYKKFLNSLID

DALEGKRTTNPWKGIL

>WP_337313173.1 MULTISPECIES: hypothetical protein [unclassified Variovorax]

MTVTIDIDPAFSRPVGGGGRLVGYNNADSVSMLYFPSDLGPAEKTWHMSHEQYEELRQAQPTLELPELKPRKPGTNANGN

TLGSVVGETPSTAGKATQANPEVKPETPKEEGWWGSWGSSVVHTVLDVGGAIPVVGIFSDGINAGIYAAEGDMVNAAISG

ASAAANLIPGGGAATKAGKLAYKGGKMALEKAEKELAEKLLKEQLEKEAAERLAKEKLEKEAAQKAEAGGGGGSKKNDSQ

VKKGKKPKCGGRATYGKQAGDFLGNEMERDHIPNVASLKDMAEKMLREDGIDLTPAQRDSLFGKVGKNGQREGGKIYTHA

QTVAVPKEIHRASPTTGNHENYKDSKDLAGAARRDTRNHRDAIEKSDNHKDCAKALEEASEVIDKISNDQYKKFLNNLID

DALEGKRSTNPWKGIL

>WP_093200219.1 hypothetical protein [Variovorax sp. YR750]

MTELTTSATFVPTGDGGSIGIAFIPADVPKPVVIMRYPTYPPQGEPTSFIQLSQEEYEKLQRDTPNVKLPDFKPRKPGTN

ANGNTLGPAVGDTPFTQVPAKQANPEVKPETPKEESWWSSWGSSITHTVLDVGGVIPVVGIFADGANAAIYAAEGDYVNA

AISGASAAANLVPGGGIAAKAGKVAIKGALKGGERVGIKQAEKLLAERIAKEKLEKEAAEAAAKKAEKEAAAAKAESGGG

SKNKDTQVKKKVKPKPKCGQKGPYKDRSKHDNEGMNWDHIPSQKALLERAAEIKGAPLTKAEKAAIVDNAPTIATPTELH

RDHSESFGGRQHQKIDGVRRPTRDAQDLQRAAKENTEKILQEIDKYDPGCKGKYREAAEEITKMTHDQWTKWLKQAMKSA

RK

>WP_367070301.1 hypothetical protein [Pseudomonas asiatica]

MSEQPIQLDTLVVRVREIPLRKIRGGPDRMFVQTDGTGKVLVDFADGVDVWGKALELNEAEYEAVRQEAFRMELADLDGI

PTYTELYPSSRSGGAATTPNGNTLGRVNGETPASGKAANDANTEVASETPAEGVTGEEILDGVQLGLDIVGLIPVVGEIA

DIANAGISLARGDYAGAALSLVSAIPFAGYVGTAGKVGRHAAKATEEVSGKATKEVVEKRARKEVSEKAERKSQAVVAGG

GKKERAPGGQSKAKKPLDCGQKGTYGQLSSFDAAGVRMERDHIPPKAALVARALELAKKSRQRLTKSQIDALKGKIERHA

QAVSVPVEIHREGITNSNPENKKYSGDLQGIAKDESRNHRENMRRSDKNHRCLGALKKATGEIGKITNKQYDDFLEKLIK

DSVRGSRTTNPWKGIL

>WP_125947317.1 MULTISPECIES: hypothetical protein [unclassified Variovorax]

MTELTTSATFVPTGDGGSIGIAFIPADVPKPVVIMRYPTYPPQGEPTSFIQLSQEEYEKLQRDTPNTKLPDFKPRKPGTN

ANGNTLGPAVGDTPSTQVPAKQANPEVKPETPKEGSWWSSWGSSITHTVLDVGGVIPVVGIFADGANAAIYAAEGDYVNA

AISGASAAANLVPGGGIAAKAGKIAIKGALKGGERVGIKQAEKLLAERIAKEKLEKEAAEAAAKKAEKEAAAAKAESGGG

SKNKDTQVKKKVKPKPKCGQKGPYKDRLKHDNEGMNWDHIPSQKALLERAAEIKGAPLTKAEKTAIVDSAPTIATPTELH

RDHSESFGGRQHQKIDGVRRPTRDAQDLQRAAKENTEKILQEIDKYDPGCKGKYREAAEEITKMTHDQWTKWLKQAMKSA

RK

>WP_023533559.1 hypothetical protein [Pseudomonas putida]

MNKQPVQVDTLVVQVREIPLRKLPNGPERMFVQTAGTDRVLVDFADGVKAWGKALELDEAGYEALRQEAFRMELADLDDI

PTYTELYPLWNYGGTTTTPNGNTLGRVNGETPASAQAANDANPEVESETPSEGVTGNEILDGIQLGLDIVGLIPVVGELA

DVANAGISLARGDYAGAALSLLSAIPLAGYAGSAGKVVWHATKATVETTGKAAREVAEARVRKEAKNATGAEKKEIAPGG

RTKAKKPLDCGQKGSYGQLNKFDASSGEMERDHIPPKAALLQKAVDLYEKSGRTLDPDELKTLARKIERHGQAVSIPKDI

HRNGITHSNPNNKKYSGDLQAVAREESRNHRENMRRDDKNHRCMAALQKATGELGKITNKQYDDFLRKLIKDTLSGKQTR

DPWKGII

>SEF30414.1 hypothetical protein SAMN03159371_04791 [Variovorax sp. NFACC28]

MTELTTSATFVPTGDGGSIGIAFIPADVPKPVVIMRYPTYPPQGEPTSFIQLSQEEYEKLQRDTPNTKLPDFKPRKPGTN

ANGNTLGPAVGDTPSTQVPAKKANPEVKPETPKEESWWSSWGSSITHTVLDVGGVIPVVGIFADGANAAIYAAEGDYVNA

AISGASAAANLVPGGGIAAKAGKVAIKGALKGGERVGIKQAEKLLAERIAKEKLEKEAAEAAAKKAEKEAAAAKAESGGG

SKKKDTQVKKKVKPKPKCGQKGPYKDRLKHDNEGMNWDHIPSQKALLERAAEIKGAPLTKAEKTAIVDSAPTIATPTELH

RDHSESFGGRQHQKIDGVRRPTRDAQDLQRAAKENTEKILQEIDKYDPGCKGKYREAAEEITKMTHDQWTKWLKQAMKSA

RK

>WP_301508654.1 hypothetical protein [Variovorax sp. CAN15]

MTVELNINPAFTRQVGGGARLVGYNYADSVSLLYFPSDVGPAEKTWHMSHEQYEELRQAKPTLELPELKPRKPGTNANGN

TLGPVVGETPSTQAPAKQANPEVKPETPKEESWWSNWGSSITHTVLDVGGVIPVVGIFADGANAAIYAAEGDYVNAAISG

ASAAANLVPGGGIAAKAGKIAVKGALKGGERVGIKQAEKLLAERIAKEKLEKEAAEAAAKKAAEEAAAKKAAEKKAGAEA

ESGGGGSKKKDTEVKNKGKKPKCGGRATYGKQVGDFLGNEMERDHIPNAASLKDMAEKMLAEDGIELTPSQRDSLFGKVG

KSGQREGGKIYTHAQTIAVPKEIHRASPTTGNHDNYKDSKDLAGAARRDTKNHRDAVAKSDNHKDCGKALEEASKVIDKI

SNDQYRKFLDKLIDDALKGKRTTNPWKGIL

>WP_044250022.1 PAAR-like domain-containing protein [Chondromyces apiculatus]

MADNEGTRKSGNFWVVSIQDDVCLTPVGDESIPIPYRIRARLTDSVSDATTVQMKAFDTSTMASRIERVEGDEEGTDGGI

NSGVNLGYCRPITHSTTVRAEGSYVIYRSSHYWMNCDGPDGLGNTFGEVVFLPDGVEPGVGPLVDLDAMREEAEKSWWDD

ASGWVHAGLDVAGFIPGVGEIADGLNAAIYLAEGDYANAAISAAGMIPFAGAAATGGRLAARAGKAALGTAAEKGTQRIT

RETAEEVAARTTREGTEEAAQQGAKQTDGVEVNGGSGGKGDANAGKGDGPDKPGDSSCSSKKDCTAEGHPVDVATGEVVD

EATDLSLPGVIPLVIERRYAASRRDHRDAALGPGWSFSLEQWIEEREASLALRDGEGRWIHFDKVEAGGASFHRRERLEL

RVGGDGTYRVHDPEKRLTRVFEGTGPKALLSAIEDASGNAIRLHHAGGVLARVVDTTGREVVCTWEGGRLTRLDAWVEGQ

VAQSVAYRYAKAGTLAAVVDAVGGEEAYEYDGSDRMVLARLKNGVCFQYAYDDESGRCVKTWGPDGLHEVALYADLGAGT

TLAESEEPKVYTWNDAGLVTREETTTGEVLAERVYDADLFVVEERNGAGDATRFERDARGHLLRVVDAAGNATAWRYEGD

RPVARTGPDGLETTYGHDAQGNLVEVRFPTGVTYALSHDARGRLSAIAGPEGPVVQYEYDRQHNVIAETDARGARTAFTY

DGLGRPTAMTDALGRTTRLTYDRLGRPVELQQPDGSTTRQTYDAQGNVASVTDALGQVTRMEYAGTGVLTRVTQPDGGCW

SFRYTSKERLREIENPLGEVYAFTYDAAGRLVTERTFDGRVLRYGYTKAGHLARIDYPDGSFRAFNHDRLGNIILEHSSD

GMATYPRDALGRMLAAVVEEGERKVVTMFERDAFGRIVTERQGDKAVRFAYDARGRRTKRVLPNGATTHYAHDALDALVG

VEHDGYRLDIERDVLGRERWRGSVGGPVSIQSQYDAMDRLIEQQVAASAPGTEIAKSVITRAWSYDALGRVTQIQDGRWG

ATTYRYDRIDQLVEAQRGSLREVFAYDAGGSLRKMLAGLEAEVGGAAWEVAPGNLLEQTERAKYTYDARGRRVVKLALGS

RDEGAEAAATAYTWDCRDRLREVRQPDGTRVRFSYDAFGRRVRKEVVPDGAETAARVVDFVWDGDELAADLERARGVRVF

VHVPGTFVPLLQSEQGQVFAVVNDHLGMPKELVGPDGRVAWSAAHSAWGRVVEVWRDPAAQQKQAVESPFRLLGQYADEE

TGLCYTRFRYFDPEVGRWCSPDPLGYAGGDNLSGFDGAPSFKVDPLGLQSDCPSDGNGSQKKARNRPPDPLPEAEGRPHT

IIEKPGPEGQYTTHNGDGTFKQYRGSGKPHYDIPRPNVKENKLNTTPDGKAIPGRPTVRPAKPDEIPGGG

>HAF2404054.1 hypothetical protein [Salmonella enterica]

MGTGNTTGKIVGQPPVPEKTAEEANPVVSAETKEEKGFFASLFDLNTEFRGRWLAVSALHHGQYESFRATAESFGDKALA

GFDGVYNSADVQFASVMVSTMGVGRSGGAPGAGSKSSAAGGRFRGTGFGMQTPALAGAGGMPYGQGIQILNAERRAAESA

VKKVEKTTGSAVKKAEKEVVKDKPNVGGKSKVKKKQKPHKKCGNKEKYTNNYKEKKVMNADHAPSGAALKKAAENKLKEM

GIWNKLSAEERKSILNKVYNDAPTISIPEDIHKKGRTYGGKNKSTQSSMDAKDLNGAFKKDTDLIQELMDASEKGCSEAY

KKSVDELSKMDWNKYINDTVTSHKNVAQFIRSSKI

>WP_068639418.1 hypothetical protein [Thauera butanivorans]

MSGTTVFAHAPVALPNGTAVAIDALESGVLLETFDPQTPQATLLSETLSYEQYAQLRADNPGIAYPELSKPDPDSQGGGG

GKEAAQQGRIHPNGYPQPGPVANTNGIIVPKTETAAQGAGSNPSGGAETEAEQGWWKSWGSGVLHGALDIAGLLPGLGEI

ADLANAAIYAAEGDYANAGISAAAAIPFAGWGATAAKAGKRVGDAVDASRTAKAGAEAGAKAGRQGAEAAGNAAAKRADG

TPSGGGRVDGNGNANPNNGKADKPCAC

>WP_163124282.1 hypothetical protein [Burkholderia cenocepacia]

MSDGALTLTGEIRKGFRSLPNGNTLGFWSQQNGVVMQEWQGRDGTGDLLTQETYDFDRYAQYRADHPDLKLPDFSPPKSG

NTIGKVVGEPSVTLPTITVTANPPPKPETPAEGGFWGAVGGWVHGGLDAAGLIPGLGAVPDLLNAGIYAVEGDYVNAGIS

AVAAIPVVGDAALAGKYAAKGGKLALKESEKLAAKEAEKLAAKEAEEKAAKEAAEKTSKKTEKEAAKEGEDGAKSEGKDN

LKCGDSGTYGDLLKQSGGNKFDRDHVPSKAALKQTAADLIDEMGIVLTDAQRKTLFGTKTSPGLIAKEGEASAIDKLDHS

GVSRTYGNRNSETQIADDAKDLQKAAGKDTKEIMDNATQWGEKCREKYEKWAEKILKKTHEEYRNDLLKLIKQVISKGK

>WP_067271579.1 hypothetical protein [Mitsuaria sp. 7]

MGSYNTTGVLQKPAASAGKAEDANAAVKVETMEISKLDALQMLLDVVGLIPGLGAPADILNGLISVARGDWIGAGLSLIG

VVPIAGEAAVAGKIAKNADRYAAGVRKVADEILPHLPEGVQRKLRDAIEKAEAKIDELAGKEAKPKAKPDEAPKAKDEGD

DGFKGKPKPKPKCGQTGPYKDRDSHDNAGFNWDHVPSKAALLKRAEELAGDVLSKDQIKAIIENAPTIAIPDKLHQKHSE

TYGGRQNQIVDDERRIKRDSGNLQRAAKENTDALLKHVDDFDPGCKGAYSEAAKAFSAITNDDWDKWLKKTMKAAKKK

>WP_373374227.1 hypothetical protein [Burkholderia ubonensis]

MSGAGNTLGRVVAADELPAIEVTAANPAVKAETAAEKGVWGSISGWVHGGLDAVGLIPGLGAIPDLLNAGIYAAEGDYVN

AGISAVAAIPVVGDAALAGKYAAKGGRLLAKEGERLAVKEGEKVAAKETEKVAAKGEKEAVEETEKQGAKEAEHEGGAGS

EPSKGQERNKVQKKKKMKCGEYGRYGKLKNKTGENKFDRDHIPSKAALKERAKFLNDGQDLSSAQKKAIDDWGEAIAIPR

RAHIDVSPTYGQTTAEAAKDAKNLAGAARRDVEAMLGKIEEYDADGGCKTAYKKAAARIMRMDNQAFDDALIKILEKVQ

>WP_321818260.1 MULTISPECIES: PAAR-like domain-containing protein [unclassified Paraburkholderia]

MASRLGARKDSAFKAVSTAPSINKTPVGSSTPPLPYPVTEDLSGSLGTVPNVRFNGDPAYVLNQSTQPACKGDAAGSCNG

VKSGTVSGEVKPVEGSSTVRITGKPVIREGDPCTLNGGNCPGIYVTQSAPGASIEGGKPSASCNPPVKPETPEEEGWWGT

ASPWVHGALGVASFVPGLSLVTGAVDAGIYAAEGDMVEAGLSAASMIPGGKVVTTAGKLAKGAVSLAKGAHAAEEAAKAA

RLAKDAEDAAKAAKLAREAEEAAKLKKSEEEAARLKNAEGDANTGTKDRDGKKVKGKKKLKCGEYGKYGDLKKKTGDGKF

DRDHIPSKAALKERAESMLDEGEKLSPGQRKAIEDWGDSIAIPRQAHIDVSPTYGAKNIGLAPQDADNLAGAARRDVESM

LSKIDEYDADGGCKKAYQKAAKRVLRMTNRDFDKALLEIIQKVK

>WP_127960406.1 hypothetical protein [Serratia microhaemolytica]

MVVRIEITRGYDTDTFCQEFGLANLQDAERARANIDELFAQKEGLQESHRDWFTDVADRTVAKFKSGEYPLAQSNTGTGN

TIGKVVGSPPTATGQAEDANPATLPETKEEKGWWEAAKDWGAERWQEVKAAAGDPVQGGIGAAKGTWNMVPDLGELMAKG

GLYQSAAELEQTAALYSLFGQEGMAQQTREAAQLTRDAVDAVSFDAIRLEMSNPAQAGGDVIATTVSLATGIGGLAKSGG

KAIAKGVTGAASHAGKTVASETVEVVAKRASNIERASTEAVETTAARAGSKAGDNVAEATGNNTAKNADNAVEKNAKPVE

EKKHTSDNADAPKKPDEPPPGAKVKQTKTHPDCGKVSKYYKAPKKLGKLNADHVPSGAALKEATKNKLKELDIDLLDSEL

ERVLDSVYRNAETITIPEDIHKEGRTYGNKNKGLYKGDSKDLKGAFKKDTASIQKVMDTKEHGCSEAYAKAVEQLSKIDY

DEFILNTLRRNKYTKGKGGLAQ

>WP_102612742.1 PAAR-like domain-containing protein [Trinickia soli]

MASQLGARKNAKFKAVSTAPSINKTPVGGSTPPLPYPVTEDLSSSVGIVPNVRFNGDPAYVLSQSTQPSCIGDAAGSCKG

VKSGTVSGEVKPVRGSSTVRISGKPVIREGDPCTLNGGNCPGIYVTQPAPSGSIAGGQPSASTNPPVKPETPKEEGWWGK

ASPWVHGALGLASFVPGLGIVTGAADAGIYAAEGDMVDAGLSAASMIPGGKIVTTAGKLVKGAAGLAREAHAAEEAAKLA

REAEAAAKAAKLEKEAEEAAKLKKAEEEASHAKKADEEATDAGKGEDGAKVKGKKKLKCGEYGKYGDLKKKTGDGKFDRD

HVPSKAALKERAESLLDEGEKLSSAQKKAIEDWGDSIAIPRRAHIDVSPTYGAKNLKLGPQDAEDLAGAARRDVEAMLKQ

IDEYDADGGCKKAYQKAAKRVLRMTNRDFDKALLEILKKVK

>WP_093212834.1 MULTISPECIES: hypothetical protein [unclassified Variovorax]

MAFSEGWYATPSQGARSFQYFKTPELVTAQGYFSGNDQILQWGSEQFPQGQPGHWTPDLKPLQKADSGDWWDNPETDKKP

KPSLDPRAGGTNANGNTVGPVVGETPSTQGSAKQANPEAQPETPKEESWWGSWGSSITHTVLDVGGVIPVVGIFSDGANA

AIYAAEGDYVNAAISGASAAANLVPGGGIAAKAGKIAIKGALKGGERVGIKQAEKLLAERLLKEKLEKEAAEAAAKKTEE

AAAKKAAEKKAGAETEAGGGGSKKKDTEVKNKGKKPKCGGRATYGKQAGDFLENEMERDHIPNVASLKAKAEKMLLDDGI

DLAPSQRDALFGKVAKNGQREGGKIYTHAQTIAVPKDVHRASPTTGNRDNHKDSKNLAEAARRDTRNHRDAIKKSDNHKD

CAKALEEASEVIDKISNEQYEKFLNGLIDDALKGSRTTNPWKGIL

>WP_093133453.1 hypothetical protein [Variovorax sp. OK605]

MAFSEGWYATPSQGARSFQYFKTPELVTAQGYFSGNDQILQWGSEQFPQGQPGHWTPDLKPLQKADSGDWWDNPETDKKP

KPSLDPRAGGTNANGNTVGPVVGETPSTQGSAKQANPEAQPETPKEESWWGSWGSAITHTVLDVGGVIPVVGIFSDGANA

AIYAAEGDYVNAAISGASAAANLVPGGGIAAKAGKIAIKGALKGGERVGIKQAEKLLAERLLKEKLEKEAAEAAAKKTEE

AAAKKAAEKKAGAETEAGGGGSKKKDTEVKNKGKKPKCGGRATYGKQAGDFLENEMERDHIPNVASLKAKAEKMLRDDGI

DLTPSQRDALFGKVAKNGQREGGKIYTHAQTIAVPKDVHRASPTTGNRDNHKDSKNLAEAARRDTRNHRDAIKKSDNHKD

CAKALEEASEVIDKISNEQYEKFLNGLIDDALKGSRTTNPWKGIL
